# Resequencing of Microbial Isolates: A Lab Module to Introduce Novices to Command-Line Bioinformatics

**DOI:** 10.3389/fmicb.2021.578859

**Published:** 2021-03-16

**Authors:** Katherine Lynn Petrie, Rujia Xie

**Affiliations:** ^1^Division of Biological Sciences, University of California, San Diego, La Jolla, CA, United States; ^2^Earth-Life Science Institute, Tokyo Institute of Technology, Tokyo, Japan

**Keywords:** CURE, bioinformatics, bioinformatics tutorial, resequencing, breseq, next-generation sequencing

## Abstract

Familiarity with genome-scale data and the bioinformatic skills to analyze it have become essential for understanding and advancing modern biology and human health, yet many undergraduate biology majors are never exposed to hands-on bioinformatics. This paper presents a module that introduces students to applied bioinformatic analysis within the context of a research-based microbiology lab course. One of the most commonly used genomic analyses in biology is resequencing: determining the sequence of DNA bases in a derived strain of some organism, and comparing it to the known ancestral genome of that organism to better understand the phenotypic differences between them. Many existing CUREs — Course Based Undergraduate Research Experiences — evolve or select new strains of bacteria and compare them phenotypically to ancestral strains. This paper covers standardized strategies and procedures, accessible to undergraduates, for preparing and analyzing microbial whole-genome resequencing data to examine the genotypic differences between such strains. Wet-lab protocols and computational tutorials are provided, along with additional guidelines for educators, providing instructors without a next-generation sequencing or bioinformatics background the necessary information to incorporate whole-genome sequencing and command-line analysis into their class. This module introduces novice students to running software at the command-line, giving them exposure and familiarity with the types of tools that make up the vast majority of open-source scientific software used in contemporary biology. Completion of the module improves student attitudes toward computing, which may make them more likely to pursue further bioinformatics study.

## Introduction

### The Need for Bioinformatics in the Undergraduate Biology Curriculum

Bioinformatics is increasingly an important part of research in any biological discipline (Barone et al., 2017), and there is widespread agreement that bioinformatics should be incorporated into the undergraduate biology curriculum (Pevzner and Shamir, 2009; Wilson Sayres et al., 2018). However, barriers to this exist at both the instructor and student level. Instructors report lack of training as the primary barrier to shifting their curricula (Williams et al., 2017), while research has suggested that student anxiety about computing and lack of confidence in their capabilities may act as a barrier to learning computing (Doyle et al., 2005).

The paper presents a way to introduce complete novices to bioinformatics as part of a module in an undergraduate biology laboratory course. This module is not as extensive as a full bioinformatics class, but could be part of an effort to incorporate bioinformatics throughout the curriculum, to reach students who wouldn’t otherwise complete any bioinformatics or computer science coursework. The goal of this module is to get undergraduate students engaged with bioinformatics in the context of a broader course, where they can connect the analysis of their data to something tangible they are exploring in another context.

How does this module address a gap in bioinformatics education? The vast majority of bioinformatics software used by researchers to analyze next-generation sequencing data is open-source and run at the command-line. This means that users interact with the software by typing commands into a text-based window (called a terminal), rather than through a point-and-click graphical user interface (GUI). Excellent short workshops to teach this type of command-line bioinformatics to researchers exist (Wilson, 2014; Teal et al., 2015; ANGUS, 2019), but they are primarily aimed at graduate students and researchers beyond the undergraduate level. There are several well-known efforts to introduce undergraduate students to bioinformatics including the Genomic Education Partnership (Elgin et al., 2017) and SEA-PHAGES (Hanauer et al., 2017). These efforts create genuine research opportunities for undergraduate students in classrooms around the world to contribute to scientific understanding and even earn authorship on scientific publications (Leung et al., 2015). However, they focus primarily on aspects of bioinformatics that do not require command-line skills. Students in these programs typically start with an assembled genome sequence that has already been processed from raw data, and they generally use GUI-based software or websites to finish and annotate the sequence (Genomics Education Partnership, 2020; SEA PHAGES, 2020). While finishing and annotation are certainly important components of genome bioinformatics, there is still a need for instruction focused on the command-line skills to needed to work with raw sequence data.

Working at the command-line can be difficult and intimidating for novices, so several GUI-based platforms that simulate command-line bioinformatics pipelines have been developed (Hilgert et al., 2014; Batut et al., 2018). While these can be used to perform real analysis and introduce the underlying concepts, alone, GUI-based platforms cannot fully prepare students to handle working with bioinformatics data the way it is done by most researchers. There are curriculum modules in the literature that focus on quantitative analysis of sequencing data using statistics-focused computing languages like R (i.e., Peterson et al., 2015; Kruchten, 2020). This module complements those modules by focusing on the data processing and analysis steps that would need to be run before (or in lieu of) that type of quantitative statistical analysis. This module aims to a fill a need in bioinformatics curricula by showing students how command-line software tools are used to go from raw sequencing data to interpretable outputs.

### What Types of Courses Could Use This Module to Bring Bioinformatics Into the Classroom?

What types of courses would be a good fit for this module? An undergraduate microbiology lab class that includes, or is thinking of including, a CURE would be ideal. CUREs, or Course-Based Undergraduate Research Experiences, incorporate genuine open-ended research of potential relevance to the scientific community (Auchincloss et al., 2014). They have been lauded as a way to answer calls to incorporate more of the skills used in science into the undergraduate curriculum (American Association for the Advancement of Science [AAAS], 2011), and they contribute to making science more inclusive (Bangera and Brownell, 2017). There are many CUREs that have been developed for microbiology labs which select or evolve a novel variant of a known microbe (overviewed in the methods, below). This module would allow students to sequence the genome of that variant and compare it to an ancestor genome that has already been sequenced, an approach called resequencing. The paper combines a guide for the wet-lab preparation of microbial DNA for next-generation resequencing with a guide to the dry-lab analysis of the resulting data.

This module would be ideal in a microbiology lab, or molecular biology lab which uses microbes as a model system. Why are microbes the ideal organism for this module? Although the costs of next-generation sequencing continue to drop, it is still prohibitively expensive and computationally time-consuming to sequence and analyze most eukaryotic genomes. Microbes, on the other hand, have genomes which are generally short enough to facilitate multiplexing – combining multiple samples together so that data for an entire class of student-generated variants can be analyzed on a single sequencing run. Microbial genome datasets are also small enough that analysis of them they can be completed in reasonable time-frames with desktop or laptop computers; they do not require high-performance computing clusters or supercomputer access.

### Organization and Goals of This Guide

The methods section contains background information and guidelines for setting up and teaching a resequencing module. The first part of the methods describes how to get from a derived microbial isolate to DNA ready for next-generation sequencing. The second part of the methods introduces the bioinformatics skills needed to computationally analyze next-generation sequencing data.

Neither the preparation of DNA for next-generation sequencing, nor the computational analysis of genomic data are novel methods, however, this article attempts to bring all of the relevant information together in one place in an accessible, easy-to-use format. We have provided a detailed lab manual with bioinformatics tutorials, lecture slides, and lecture notes in the Supplementary Materials. Instructors can use the module as-is, or they can use it as a starting point to be adapted to their own particular purposes. Although specific details of sequencing methodology and software may change over time, this article covers several universal considerations that should guide any instructor thinking of incorporating a resequencing and bioinformatics module into their class.

This article is intended as a guide to help course designers and instructors who do not have prior next-generation sequencing or bioinformatics experience bring a resequencing module into their own course. This approach has been vetted in the classroom over several quarters of a microbiology lab course by an instructional team consisting of a lead instructor (the author), two additional instructors, multiple graduate instructional assistants, and laboratory support staff. We show that this module can improve student attitudes toward computing, which could make students more likely to engage and persist in further opportunities to use bioinformatics.

## Methods for Implementing Resequencing Module as Part of a Course

**FIGURE 1:**
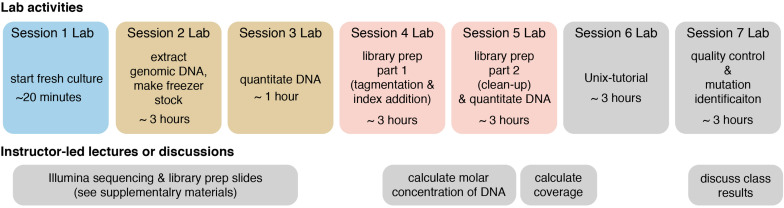
Overview of each lab session and suggested lessons for downtime or in between lab session. Lab sessions are broken up into 3 h (or less) blocks, though adjacent sessions of identical color could be combined into a single session if timing allows. The lower track shows suggested instructor-led lectures or discussions. Two sets of slides, covering Illumina sequencing and Illumina library preparation, are provided in the Supplementary Materials, and they include active-learning questions for students to attempt in-class and for peer-instruction (these can be administered through digital or informal polling methods).

**FIGURE 2:**
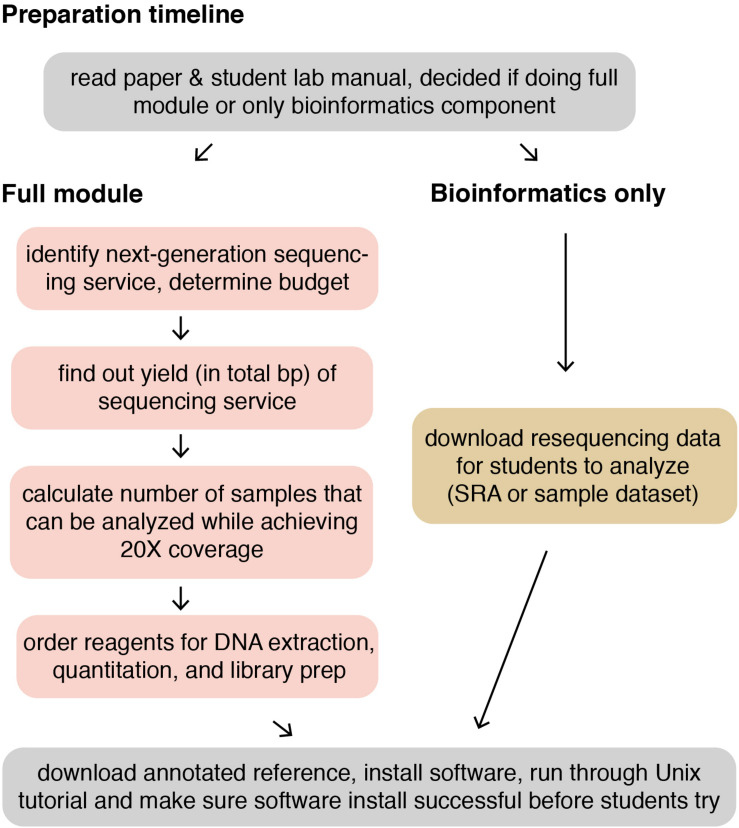
Suggested preparation timeline for instructors.

The following methods provide a general guide for instructors, covering key considerations and pitfalls to avoid for each step of the module. Detailed, step-by-step instructions for students, including protocols adapted from kit manufacturers’ instructions, as well as bioinformatics tutorials, are provided in the Supplementary Materials. For instructor testing, or for use in a course that is only incorporating the bioinformatics portion of the course (see section “Dry Lab Methods: Analyzing Genomic Re-sequencing Data”), a sample dataset has been provided in the Supplementary Materials. For courses incorporating the wet lab methods, the methods assume that each student, or group of students, has isolated a unique microbe of interest that they will characterize. Figure 1 shows an overview of the individual lab sessions, along with suggested lessons for down time or for lectures between labs. Figure 2 shows a suggested preparation timeline for instructors planning to add all or part of this module to a class.

### Wet Lab Methods: Preparing DNA for Genome Sequencing

One can imagine many possible experiments students could run to generate or isolate novel derivatives of a microbial strain. One popular example is the isolation of antibiotic resistant microbes in laboratory selection experiments. Another is culturing the fast-evolving *Pseudomonas fluorescens* SBW25 strain in static microcosms to study the evolution of biofilm-forming phenotypes (this is, in fact, what we did in our implementation of the class). Details on how to set up those experiments for the classroom are provided elsewhere (Green et al., 2011; Spiers, 2014; Johnson and Lark, 2018; Van den Bergh et al., 2018), so they will not be included here. Once a strain of interest has been generated, the following steps provide an overview for educators of how to prepare DNA from that strain for sequencing.

Generally, it is important that students use good sterile technique, especially when they are working with the bacterial strain itself. Once cultures are grown and DNA is extracted, there are not as many opportunities for exponential amplification of contaminants, but students should still practice clean laboratory technique to avoid cross contamination and prevent the introduction of DNases (Students should wear gloves at all times, always use fresh micropipettor tips for every step of a procedure, and take care not to touch the inner caps or rims of microfuge tubes).

For preparation steps which use kits, the instructional team found it useful to pre-aliquot reagents per pair of students or per group (providing a slightly higher volume than required) to speed up time in the classroom and avoid cross-contamination. The adapted protocols provided in the manual are aimed at students; for any kits utilized, we recommend instructors also read the kit manuals provided by the manufacturer; they contain important details, like storage conditions, not provided here.

#### Initial Considerations – How Many Samples Can You Sequence?

The biggest consideration for introducing a resequencing module into a laboratory class is how many individual isolates to analyze. One of the benefits of a CURE is that students have the opportunity to make a genuine scientific contribution. The more isolates there are, the more there is to potentially learn, and students working with their own unique microbe may have a greater sense of ownership over their project. However, these goals must be balanced by cost, time, and the amount of data required per strain to get meaningful results. In our implementation, the cost, from start to finish, was approximately $100 per isolate sequenced, though this may vary according to the specific kits and sequencing service used (many institutions have a core facility that provides next-generation sequencing services, there are also several commercial vendors). In terms of time, several of the processing steps are fairly work intensive, so we recommend students work in groups of 2–4 students (with one isolate per group) so they can assist one another.

The amount of data required per strain is a key consideration in determining how many variants can be sequenced. Most sequencing services require users to buy an entire sequencing “run,” where all the samples loaded onto the machine are submitted by a single user. In the author’s implementation of the module, every pair of students works with their own unique isolate, and 24 isolates were pooled together into an Illumina sequencing run. Illumina machines produce the largest share of contemporary sequence data. It would certainly be possible to carry out resequencing projects with third generation sequencing technologies, like Oxford Nanopore’s MinION sequencing or Pacific Bioscience’s SMRT sequencing, but a detailed guide for those technologies is beyond the scope of this article. For an excellent review of all sequencing technologies, see Slatko et al. (2018). For our course, samples were sequenced on an Illumina MiSeq instrument.

Why is the MiSeq an appropriate instrument for the job? The answer requires a basic understanding of how Illumina sequencing works (Bentley et al., 2008), briefly reviewed here. To prepare DNA for sequencing, genomes are randomly fragmented into smaller pieces and Illumina-specific adaptor oligos are attached. The ∼600 bp-long fragments are then loaded onto a flow cell coated with a lawn of oligos complementary to the adaptors. Fragments are loaded at low concentration so they are well-isolated from one another when they anneal, and then they are clonally amplified in a 2-dimensional PCR-like process to produce DNA clusters. Each cluster contains enough template to make visualization of base-specific dyes possible in a subsequent sequencing-by-synthesis step. All clusters are visualized simultaneously, with images taken after each base is added. The resulting stack of images is then converted into a digital sequence, called a “read,” corresponding to each individual cluster. The determining factor for how much data a particular instrument can put out is how many individual clusters can be visualized in a single run on that instrument. The MiSeq instrument can generate ∼22 million reads per run (Illumina, 2018).

How do we use that information to figure out how many samples to run? First, we need more information about the read-length — how many bp of the DNA strands in each cluster are actually sequenced. Usually, the entire length is not read; with the most recent reagent kits, 75 or 300 bases are read from each end (Illumina, 2018). This results in a pair of reads for each DNA fragment. Once we know the read-length and the number of reads, it is possible to estimate the coverage — how many times, on average, each position in the genome will be represented in the data. Coverage is calculated as the total number of bases sequenced/the genome size of the organism. For resequencing, 20-fold (or “20X”) coverage is a safe bet, as coverage is not always uniformly distributed across the genome, and extra coverage can help distinguish rare sequencing errors from genuine variants. In our implementation of the class, we sequence 24 samples of *P. fluorescens* SBW25 in a single, 75 bp paired-end run; this corresponds to ∼20-fold coverage. [(22,000,000 reads × 150 bp per read)/6,722,539 bp in SBW25 genome] = 491-fold coverage/24 samples = 20-fold coverage).

#### Genomic DNA Extraction – The Fresher, the Better

Students should begin with a clonal isolate of their strain of interest — ideally in the form of a well-isolated colony on an agar plate. For laboratory evolution and selection experiments, the starting, or ancestral, strain should also be processed as described here, even if a reference is available (Strains may accumulate mutations as they are propagated and stored in labs over time, and knowing the precise sequence of what you are starting with is crucial for interpreting mutations. The sample dataset illustrates this point; the SBW25 strain we used to initiate evolution — the ancestor — is slightly different than the public reference available in GenBank).

While it is possible to isolate genomic DNA directly from a colony, it is easier to achieve the high yield and quality of DNA necessary for next-generation sequencing by first preparing a fresh saturated culture (∼10^9^ cfu/mL, typically from overnight incubation). If lab sessions are scheduled so that students will not be able to come in on contiguous days, inoculated cultures should be held at 4°C and only transferred to an incubator for growth the afternoon or evening before students will return. Over-incubation or prolonged storage in the stationary phase can lead to the accumulation of GASP mutations (Finkel, 2006), which could make interpretation of sequencing results difficult. LB (lysogeny broth) media is recommended for this overnight growth as LB has been widely used to amplify bacteria without downstream issues in next-generation sequencing. Any formulation should work, though we have most recently used LB-Miller (Miller, 1972).

For the actual genomic DNA extraction itself, there are many different commercial kits available. A column-based kit is recommended; in student hands they were both easier and higher-yield than those which rely on phase-separation. Many genomic extraction kits have an “elution buffer” designed for the final step of eluting or resuspending genomic DNA; these should not be used, as some elution buffers, especially those that contain EDTA, can interfere with downstream steps. Instead, MilliQ or molecular grade water should be used for elution. We have had success with the QIAGEN DNeasy Ultra Clean Microbial Kit (Cat No. 12224).

#### Genomic DNA Quantitation – OD_260_ Is a No-Go

For next-generation sequencing, it is important to measure the concentration of DNA as precisely as possible. To that end, it is recommended that fluorescence-based quantitation methods be used (as opposed to UV absorbance-based methods). In our implementation of the class (as described in the Supplementary Materials), we used a Qubit fluorimeter and Qubit ds DNA HS assay kit according to the manufacturers’ instructions, but many other dyes/reagent kits are available, and they can be used on any instrument with the appropriate excitation wavelength and emission detection spectrum. One important consideration is sensitivity. The amount of genomic DNA required for library preparation, using the approach described in the next step, is 1 – 500 ng (in 2–30 μL). Given the elution volume, this translates to a minimum DNA concentration of ∼33 pg/μL, however, students have had the most success with DNA concentrations at or above 16.7 ng/μL (concentrated samples can always be diluted). It may be useful to have a backup sample of DNA available for students who do not extract the minimum amount.

#### DNA Library Preparation – Your Students Can Handle It!

There are many library preparation protocols and kits for Illumina sequencing. Regardless of the specific approach used, all protocols break the genomic DNA into smaller fragments and attach oligonucleotide adaptor sequences to the ends of each fragment. This collection of prepared fragments is called a sequencing library. The adaptor sequences help each fragment bind to the flow cell and generate clusters, and they are complementary to primers used in sequencing by synthesis. Library prep can also include a step that adds a unique oligonucleotide — called an index — to every fragment in the sample. This acts as a barcode or a tag, so that when multiple samples are pooled and sequenced together, the output can be computationally sorted by the unique index sequences.

Library preparation is the most difficult part of next-generation sequencing, and even experienced scientists in research labs sometimes elect to outsource library prep as an additional fee paid to the sequencing service provider. However, this is (often prohibitively) costly. The kit used in the our implementation of this module (Illumina DNA Prep, 20018705, from Illumina) was chosen primarily because of its ease of use. Fragmentation and adaptor ligation are carried out in a single step (cleverly called “tagmentation”), and the bead-based purification is somewhat self-normalizing, in that the beads can only bind a certain maximum amount of DNA; so as long as they are saturated, different students should get fairly similar yields.

The Supplementary Materials contains detailed directions adapted from the kit’s manual. Normally, library prep kits are designed to allow a single researcher to process up to 96 samples at once, using multichannel micropipettors and 96-well plates. Here, they have been rewritten to allow students (or groups of students) to process their samples individually, with standard micropipettors. Below are a few key pointers:

Amount of input DNA: the kit is designed to accommodate 1–500 ng of DNA, added in anywhere from 2–30 μL of liquid volume. As mentioned before, students should add the maximum amount of DNA possible. However, students new to the laboratory may have difficulty calculating what volume to add, so it is useful to have instructors or assistants check student’s calculations before proceeding (see the Illumina Library Prep slide deck in the Supplementary Materials for examples of this calculation). If less than 50 ng was added, students will need extra amplification steps, so they should pay attention to the note in step 21 of the session four protocol.

Magnetic bead-based purification: most kits rely on ferrous microbeads that bind the DNA. When tubes are placed in magnetic racks, the beads are immobilized while solutions are exchanged. Magnetic stands typically use strong, rare-earth magnetics to speed up the separation process. We have had success with eight-well magnetic stands shared among groups with four students each. We use stands which orient the magnet on the side of the tube (rather than at the bottom or in a ring), as this allows students to rest the pipette tip against the opposite wall without disrupting the beads. The particular stands we use are not currently available from the supplier, but there are many different commercial sources and DIY plans for constructing your own (Oberacker et al., 2019).

Index addition: Typically, samples will get two unique indexes: one for each end of the fragments. We have successfully used the Nextera CD indexes (Illumina 20018708). Indices are typically supplied in trays or in a limited set of tubes, which are difficult to share with students. We have students bring samples to the instructor or an instructional assistant to receive their unique indices one sample at a time. This prevents cross contamination and allows the instructor to record which samples get which indices, which is useful if students misplace this information. To attach the index oligos, the number of PCR cycles needed varies depending on the amount of DNA originally used as input; it is important to make sure students use the correct number.

Stopping points: the complete library preparation process is fairly time consuming, however, it can be broken into two lab sessions, with the DNA stored at 4°C after the index addition and amplification step. We have stored DNA at this stopping point for up to 5 days with no problems, however, there are no other recommended stopping points during library preparation.

#### Figuring Out the Molar Concentration

Although the Illumina DNA Prep kit is designed to normalize the yield of library DNA, when carried out by many different student groups, we tend to see a fairly wide range in the library yields. So the library DNA should be quantified using a fluorescent-dye based method, as described above. Additionally, it is typically recommended that the average fragment size of the library be measured with either a TapeStation (Agilent) or BioAnalyzer (Agilent) instrument. This is because the tagmentation may not always produce fragments of the exact same size. However, if you do not have access to one of these instruments, it is acceptable to use the average expected fragment size, which for the Illumina DNA Prep kit is 600 bp. The average fragment length is used to calculate the molar concentration of DNA, using an average atomic mass of 660 g/mol for one basepair. The molar concentration will be used in the next step.

#### DNA Pooling for Sample Submission – It’s All About Balance

To take advantage of small microbial genome sizes and maximize data yield, samples are multiplexed: pooled together and run on the same machine. To make sure that each sample is equally represented in the sequencing data, it is important that an equal number of DNA fragments are added from each sample. This will require students to dilute their DNA to a universal concentration before their sample is added to the pool (Alternatively, different volumes of each sample can be added to achieve the same final concentration). The sequencing service provider will specify the required total concentration of DNA in the pool; it is typically at least 10 nM. Because some students may have lower than expected amounts of DNA, we recommend instructors be the ones to collect the final concentration of each library from students and calculate how the DNA should be pooled. It may be necessary to add a little less of some high-concentration libraries to “make room” for low-concentration libraries, and some very low concentration libraries may have to be dropped altogether, if they fall too far below the threshold required by the sequencing center. Once a pooling scheme has been established, we recommend that students bring their samples to the instructor or an assistant to be added to the pool one-at-a time. This prevents cross contamination and lets the instructor “check off” each sample as it is added. If you are exceptionally lucky, your sequencing service provider may offer to pool your samples for you, but if they do, you should verify whether they will account for different sample concentrations, or else the data may be dominated by the highest-concentration libraries.

### Dry Lab Methods: Analyzing Genomic Re-sequencing Data

It may take several weeks to get data back from your sequencing service provider, so it is important to start the wet lab portion of this module early in the course. Most sequencing providers will demultiplex the data for you (separate it into individual files according to the unique index barcodes for each sample). Typically, the size of sequencing data files is large enough that sharing via email or an LMS may be problematic, so the service provider may provide an ftp link that could be shared with students, or data can be distributed from a central source using USB storage devices.

To provide a universal computing environment for all students, ideally analysis would be carried out in a computer lab with software preinstalled. While it is possible to set up virtual machines that can be downloaded or accessed through cloud computing so that students can use their own devices, instructions on how to do so are beyond the scope of this article. A Unix-based operating system (OS) is required, as most open-source bioinformatics software cannot be run directly on PCs. This means that you can use Unix OS, Linux OS, or Mac OS. In principle, you can use a simulated Unix environment on a PC through the use of an interface like Cygwin^1^, though this will be more challenging. If you plan to have students use their own machines, we recommend setting aside at least an entire lab session to help students configure them, and we recommend skipping the “Quality Control” section below.

#### Working at the Command-Line – A Guide for the Complete Beginner

The Supplementary Materials include a brief tutorial that introduces students to the basic commands used to navigate through directories (folders) in Unix-based terminals. It is essential that students try it out on their own (rather than follow along with the projector while an instructor demonstrates), as engaging with the activity and seeing for themselves what actually happens is key to understanding some essential rules about working in a command-line terminal.

Many instructors or teaching assistants may be apprehensive about teaching bioinformatics if they are themselves new to working at the command-line. However, trying the tutorial ahead of time and seeing what common mistakes occur is sufficient preparation for most of the problems students might encounter. The vast majority of errors stem from typos or from commands that try to use a file not located in the current working directory. A quick check of the command that students entered and a look at the file contents of their current location reveals most problems. We have provided a troubleshooting guide expanding on this and other common problems in the Supplementary Materials. More complicated issues can usually be solved by reading the error messages, and occasionally using a search engine to find out what they mean. Additional information can be found through discussion forums focused on computing (Stack Overflow, 2020), bioinformatics in general (Biostars, 2020), next-generation sequencing (SEQanswers, 2020), and on the support pages for individual software tools. Even experienced bioinformatics researchers have to troubleshoot software, so it is good to adopt a collaborative outlook to helping students solve problems, encouraging them to be resourceful and not get discouraged if things don’t work out the first time.

#### Installing Software – Use a Package Manager if Possible!

Software installation is probably the most difficult part of next-generation sequence analysis. Many open source software programs are not self-contained; they require other, previously developed software programs to function. This is the nature of high-throughput sequencing analysis – newer, more specialized programs build on earlier algorithms and data processing tools. The software tools required by a particular program are called dependencies, and up until about a few years ago, there was little else to do but install each dependency – and the dependencies of that dependency – one at a time. One would have to hope that all of the versions of each piece of software were compatible with one another, and if not, just keep troubleshooting until it all worked. Software developers and users refer to this problem as “dependency hell,” and it is particularly vexing in bioinformatics, since software tools have been developed by independent research teams over nearly a decade and a half of next-generation sequencing history.

So, how do we make this easy and accessible? Fortunately, tools called package managers have been developed to make software installation easier. With a single command, they can install the desired software program and all of its dependencies automatically, and package managers can be used to create “environments” – workspaces for individual projects with defined collections of software. In recent years, the bioinformatics community has assembled bioconda, a collection of packages (software, dependencies, and directions that tell the computer how to install them) for over 7,000 bioinformatics software tools (Grüning et al., 2018). The packages in bioconda can be installed with the popular package manager, conda^2^ or miniconda, a lightweight version of conda.

All of the bioinformatics software used in this module (fastqc, fastx_toolkit, and breseq) can be installed through bioconda using the conda or miniconda package manager. To use bioconda, carefully follow all the directions in the “Getting Started” section of the bioconda user documents found at^3^ (For additional information, see the notes accompanying the sample dataset). It is also possible to install the software without a package manager, by following the individual installation instructions for each individual software tool (see citations below for links to the user support). Finally, a streamlined version of this module can be completed with just breseq, to minimize the software requirements, though the breseq installation instructions must be followed carefully to make sure all dependencies are also installed. If possible, we recommend that you work with your institution’s technology support staff to facilitate software installation, especially if you will be installing software in a computer lab (most institution-managed computers do not grant regular users permission to install software by default). Note: if students will be running this module on PCs (i.e., via Cygwin), it may be easiest to skip the quality control steps and install breseq directly according to the instructions in the breseq user documentation.

#### Examining Data – An Introduction to the FASTQ Format

Illumina sequencing outputs data files in the FASTQ format. FASTQ files contain information on all of the “reads” corresponding to that sample. A “read” is the information derived from an individual genome fragment, and contains the sequence of bases, as well as a quality score for each base call.

The quality score, Q, estimates the probability that the base is incorrect (P, probability of error), according to the formula *Q* = −10 log_10_ (P) (Ewing and Green, 1998; Cock et al., 2010). This conversion takes potentially long character strings (i.e., a high-quality base call like *P* = 0.0001, or 99.99% accuracy) and reduces them to one or two digits (i.e., *Q* = 40). To compress the quality score even further, in the FASTQ file, Q is reported as single ASCIII keyboard character (ASCII characters are numbered, for example, the letter “I” is ASCII character 73). To get Q, you subtract 33 from the ASCII value, however, older Illumina data (only a concern if you are using previously collected data from several years ago) had an offset of 64 (Cock et al., 2010). The tutorial leads students through an exploration of the FASTQ format and how to interpret the “two-layer” code of compressed quality scores.

#### Cleaning Up Data – Optional Here, but Good Practice for Students

All sequencers occasionally produce low quality base-calls, and in many bioinformatics applications, it is important to filter these out. Here, the tutorial guides students in the use of two software tools, FastQC (FastQC, 2015), which produces statistics on the quality score distribution of a FASTQ file, and the FASTX-Toolkit (Hannon, 2010), which can be used to remove reads where a specified proportion of the bases fall below a specified quality score. For this module, this filter is not strictly necessary, as the next step of analysis actually takes quality score into account, so it can be safely skipped if time is a limiting factor. However, it is good bioinformatics practice to examine the quality of the data, and removing low-quality reads can make subsequent steps of the analysis run faster.

The FASTX-Toolkit filter can only be run on one file at a time. This means students with paired-end sequencing data must run it twice, once on the forward reads file and once on the reverse reads file. Because the different files may have a different number of reads passing the filter, the filtered files may be different sizes. This is not a problem for breseq, as it treats the forward and reverse reads as if they were two independent lists of single-end data. However, we would be remiss not to mention that other bioinformatics software make use of pair linkage information (the fact that forward and reverse reads are ∼600 bp apart) to guide analysis, and in other applications, it is critical that every read in the forward file has its corresponding pair in the reverse file. If you are considering other analyses, you may need to use a filter designed to work with paired-end data, such as sickle (Joshi and Fass, 2011).

#### Running Breseq to Identify Mutations – The Software That Does It All!

In order to identify mutations in the sequenced strains, the reads need to be compared to an existing reference sequence (of the ancestor or a closely related strain). First, the reads are mapped to the reference (each read is scanned against the genome to see where it belongs), and then each position is examined to see if the majority of the reads there have the same base at that position as the reference does. There is a huge variety of software tools capable of performing these steps (alignment and variant identification), but this module uses a tool called breseq (short for bacterial resequencing) (Deatherage and Barrick, 2014). A detailed guide to all of breseq’s capabilities is available elsewhere (Deatherage and Barrick, 2014); here, we cover a few important pointers.

Breseq is ideal for students new to bioinformatics, as it outputs results as easy-to-navigate html files that can be opened in a web-browser. A key feature of breseq is that it can report not just the genomic position and identity of any mutations, but also whether a mutation is synonymous or non-synonymous as well as the name of the gene it is in or near. To get this information, you must use an annotated reference in a gff3 or GenBank (.gbk) format, which includes the location and name of genes. Annotated references for many microbial species can be obtained from the NCBI. For our classes, we provide the reference file to students to ensure that they are all using the same one. To find a reference sequence at NCBI^4^, restrict the search to the “Genomes” database, and type your species of interest in the search bar. This will display the landing page for your species, and you can click a link to browse all available genomes for the species. Locate your strain (or a close relative), click the link in the “strain” or “organism name” column, and you’ll be taken to its genome assembly and annotation report. From there, you can click “download genome annotation in GenBank format.” You can also get to the GenBank record by clicking on the RefSeq ID, though to ensure that you download the full file, you will have to set the “customize view” to show all features before you download it with the “Send to” link.

Running breseq will take a considerable amount of time, as aligning millions of short reads to a genome that is millions of bp long is not a trivial task. The time required depends on the genome size, the amount of data, and the computer itself. We have found that on a typical desktop or laptop computer, it takes 10–20 min for breseq to analyze an ∼7 million bp genome with ∼20-fold coverage.

## Results

### Implementation of Module

This module was incorporated into a Microbiology Laboratory course taken primarily by juniors and seniors. It has since been taught by three different instructors (including the author) to ∼450 students (in person). In Spring 2020, the class was held remotely for ∼100 students, and we implemented only the bioinformatics analysis, relying on data generated by previous classes. Instructors wishing to run only the bioinformatics portion of the module can use the sample dataset provided (see Supplementary Materials), or browse publicly available resequencing data in the NCBI’s Sequence Read Archive (Sequence Read Archive Submissions Staff, 2011).

In every offering of the course, variants have been successfully analyzed. Identifying mutations is only the first step of the analysis; the bigger challenge for students lies in interpreting them. Students have to predict which mutations are responsible for the observed phenotype of their variant, and which mutations are neutral, acquired by random chance. This requires students to dive into the literature to learn more about the genes or regulatory regions where they find mutations. Students can also see if similar mutations have been previously observed. In our implementation of the class, we have students write up their findings in a lab report, though other forms of assessment are possible.

### Assessment of Bioinformatics Module’s Impact on Student Attitudes Toward Computing

For a subset of classes in which this module was offered, we carried out a focused assessment of the bioinformatics portion of the module. Before and after the computing module, we administered a validated instrument, the Computing Attitudes Survey (CAS), designed to measure student attitudes toward learning the practices and skills of computing (Dorn and Tew, 2015). Why focus on student attitudes? After a brief introduction like the one in this module, students will likely need further practice to really master bioinformatics content. However, if the module can positively impact their attitude toward computing, they may be more likely to persist in future opportunities to learn and use bioinformatics.

The CAS is a 26-item Likert scale that assesses the beliefs people have about the process of computing and learning computational skills (Dorn and Tew, 2015). Within the scale, items are divided into subscales, called factors, that relate to different components of student attitude. The scale includes three factors connected to problem solving: belief that concepts and ideas can transfer to new problems, attitude toward problem solving strategies, and adoption of a growth mindset (the idea that skills and understanding are not fixed and can be improved with practice). Another factor relates to belief in the real-world relevance of computer science, and the final factor assess personal interest in and enjoyment of computer science (see Table 1 for a detailed description of the five factors and sample items). Each item in the CAS has a “correct,” or expert-like rating, based on the consensus opinion (agreement or disagreement) for each item when administered to a group of computing faculty as described in Dorn and Tew, 2015. Students are scored based on their level of agreement with the expert consensus, providing a measure of how students may shift from holding more novice-like attitudes toward more expert-like attitudes (Dorn and Tew, 2015).

**TABLE 1:** The five components of student attitude toward computing (For a complete list of the items in each factor, see Dorn and Tew, 2015).

	Factor	Description	Sample item from CAS (expert consensus)
1	Problem Solving – Transfer	Ability to see/apply connection between concepts and ideas to solve problems	Errors generated by computers are random, and when they happen there’s not much I can do to understand why (disagree)
2	Problem Solving – Strategies	Attitude toward problem-solving strategies in computer science	When I solve a computer science problem, I break it into smaller parts and solve them one at a time (agree)
3	Problem Solving – Growth Mindset	Belief in ability to improve skill or understanding with practice	If I get stuck on a computer science problem, there is no chance I’ll figure it out on my own (disagree)
4	Real-World Connections	Belief in real-world relevance of computer science discipline	Tools and techniques from computer science can be useful in the study of other disciplines (e.g., biology, art, business) (agree)
5	Personal Interest and Enjoyment	Personal interest, motivation, and engagement with computer science	I am interested in learning more about computer science (agree)

We administered the CAS immediately before and immediately after the bioinformatics (“dry-lab”) portion of the module, so that each student has a pre- and a post- score. We also asked students to describe their previous experience in computing. Students’ demographic factors, including applicant type, first-generation student status, and gender, were added and student responses were deidentified. All responses were collected with approval of the UC San Diego Institutional Review Board. Only students who completed both the pre- and post-survey once and on time were included. Students who did not respond to more than five items were removed. Students who did not correctly respond to the control statement (“We use this statement to discard the surveys of people who are not reading the questions. Please select “Agree” for this question to preserve your answers) were removed, and this item was not used in subsequent analysis.

Student responses were scored according to the method described by the survey’s developer (Dorn and Tew, 2015). For each item, students selected “Strongly Disagree,” “Disagree,” “Neutral,” “Agree,” or “Strongly Agree.” The responses were first collapsed into a 3-point scale by replacing “strongly agree” by “agree” and replacing “strongly disagree” by “disagree,” then scored based on their agreement with expert opinion. Each item receive a “1” if the student agreed with the expert opinion, and a “0” if their response was “Neutral” or they disagreed with expert opinion. The score for each student was calculated as the average of their responses to all items (to get an overall score) or only those from the relevant subscale for each factor. A score of 1 represents student agreement with expert opinion on all items, and a score of 0 represents disagreement with expert opinion on all items.

**FIGURE 3:**
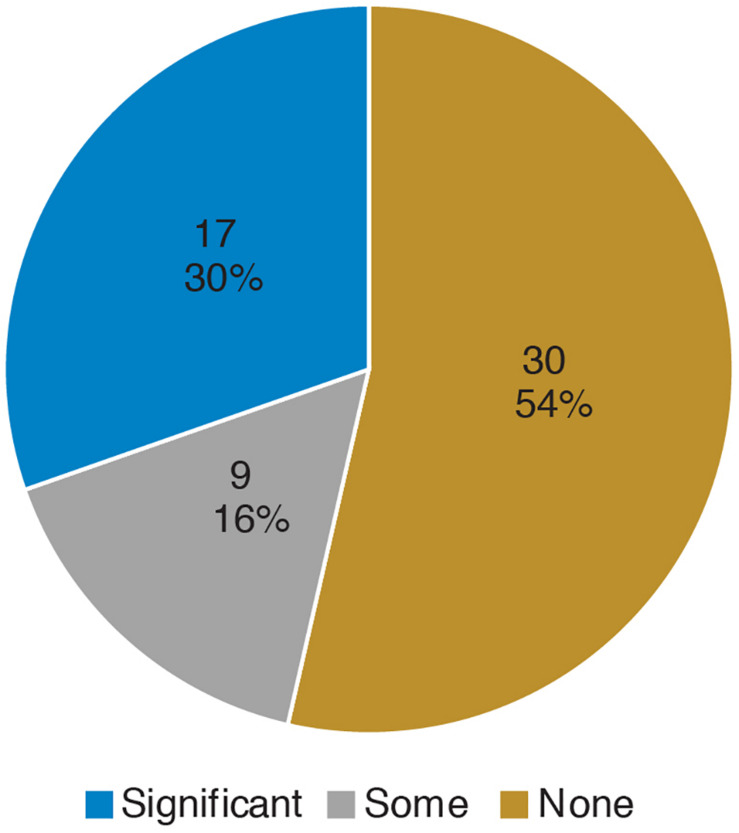
Prior computing experience of students. In the pre-survey, students were given free-response space to respond to the following prompt: “*Please describe any experience you have in computer science. Write “none” if you don’t have any experience. Some examples of computer science are: using a computer to analyze data, taking a CSE course, programming, writing software, writing computer scripts, analyzing data in R, debugging, running computer programs via command-line interfaces (where you type directions to your computer instead of using a mouse to point and click).*” Student responses were coded according to the following scheme: “None” if the student wrote none or indicated no experience, “Some” if the student described MATLAB or limited use of command-line (i.e., in personal time), and “Significant” if the student mentioned a bioinformatics or computer science course, or if they mentioned “knowing” a computer language. The number and percentage of students in each category are shown.

**FIGURE 4:**
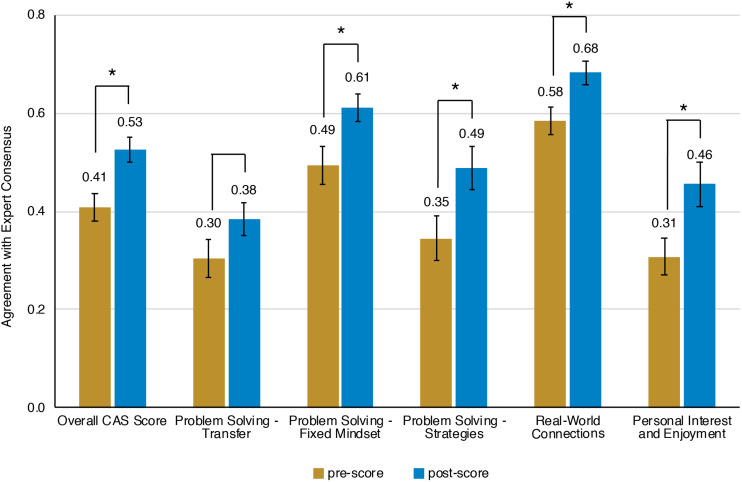
Scores on Computing Attitude Survey before and after completing bioinformatics module. Mean pre- and post- scores are shown. Bars indicate standard error. Significant differences between pre- and post- scores (Wilcoxon Signed Rank, *n* = 56) are indicated with a star.

Prior to the bioinformatics module, over half the students surveyed did not have any experience with computing (Figure 3). When looking at all students, there was a significant improvement in overall computing attitude scores after completion of the bioinformatics module (Figure 4 and Table 2, Wilcoxon signed rank test, *p* < 0.001, *n* = 56), suggesting that even this short module can improve student attitudes toward computing. Looking at all items in the survey, students went from an average of 41% agreement with expert opinion to 53% agreement with expert opinion. A significant improvement was seen in four out of the five factors, with “Problem Solving – Transfer” as the only factor with no significant improvement. Shifts in the overall CAS score were greatest for the students with no prior computing experience, though students at all levels of experience showed a gain (Table 3).

**TABLE 2:** Pre- and post- CAS scores.

Measure	Pre-score		Post-score		Shift	Wilcoxon signed-rank	
	Mean	SD	Mean	SD		*W*	*p*
Overall CAS Score	0.409	0.212	0.526	0.183	0.117	195	<0.001*
Problem Solving – Transfer	0.304	0.289	0.384	0.257	0.080	254	0.026
Problem Solving – Growth Mindset	0.493	0.288	0.612	0.213	0.119	179	0.003*
Problem Solving – Strategies	0.345	0.339	0.488	0.332	0.143	106	<0.001*
Real-World Connections	0.585	0.220	0.683	0.181	0.098	106	0.007*
Personal interest and Enjoyment	0.308	0.274	0.455	0.341	0.147	150	0.002*

*The shift of each score was calculated as the post-score minus the pre-score. The average and standard deviation for all students are shown. To see if there was a significant difference between the pre and post scores, a Wilcoxon signed-rank (paired, non-parametric, *n* = 56) test was used. Significant differences are starred with ^∗^ (for the overall CAS score, α = 0.05, for the individual factors, a Bonferroni correction was applied; α = 0.01).*

**TABLE 3:** Gain in overall CAS score by prior level of computing experience.

Prior computing experience	*N*	Mean shift	SD
None	30	0.169	0.193
Some	9	0.0756	0.0581
Significant	17	0.0471	0.143

*The mean and standard deviation are shown.*

Computer science remains one of the STEM majors with the biggest gender gap [only about 20% of CS majors are female (Sax et al., 2016)]. Studies have attributed this gap to differences in attitudes (Dorn and Tew, 2015; Sax et al., 2016). Here, we sought to explore whether there were differences in computing attitudes between male and female students in the context of a biology course. We also explored whether there were differences between first-generation college students and students with at least 1 parent with a four-year degree, and between students who enrolled directly as new freshman and students who transferred from other (typically community) colleges. In contrast to previous studies, we did not see a significant difference in pre-scores on the CAS between any of the demographic groups (Table 4). We also did not see any significant differences between demographic groups in how much the CAS scores improved after the module (Table 5). Possible explanations for the difference between our observations and previous work are explored in the discussion.

**TABLE 4:** No significant difference in overall CAS pre-score between different demographic groups (Mann–Whitney *U*-Test, non-parametric, independent, *n* = 56).

					Mann–Whitney *U*	
		*N*	Pre-score	SD	*U*	*p*
Applicant type	New-Freshman	43	0.422	0.198	227	0.312
	Transfer	13	0.366	0.259		
First-Generation Status	Non-First-Generation	38	0.397	0.207	324	0.751
	First-Generation	18	0.436	0.227		
Binary Gender	Female	42	0.386	0.204	231	0.236
	Male	14	0.480	0.229		

**TABLE 5:** No significant difference in overall CAS score improvement between different demographic groups (Mann–Whitney *U*-Test, non-parametric, independent, *n* = 56).

					Mann–Whitney *U*	
		*N*	Mean shift	SD	*U*	*p*
Applicant type	New-Freshman	43	0.1060	0.156	239	0.431
	Transfer	13	0.1538	0.217		
First-Generation status	Non-First-generation	38	0.1147	0.133	318	0.679
	First-Generation	18	0.1222	0.238		
Binary gender	Female	42	0.1190	0.176	285	0.864
	Male	14	0.1114	0.163		

*The mean shift is the average difference in pre- and post-score.*

All statistics were computed in jamovi (The jamovi project, 2020).

## Discussion

This methods paper serves as a guide for instructors who are thinking of adding a next-generation resequencing project into their courses. We hope short modules like this can act as a bridge for novice students with no prior command-line experience. Though only a small amount of specific knowledge of particular software programs is covered, the familiarity with command-line that students develop, and the positive impact of the module on student attitudes toward computing, may serve as a bridge to future learning.

This type of exposure may be particularly important for students from populations that are underrepresented in computing fields. Studies have shown that key barriers for participation are student attitudes toward computing, including their confidence about their computing ability and their perception of belonging in computer science (Cheryan et al., 2009; Dorn and Tew, 2015; Sax et al., 2016). Completion of the bioinformatics portion of the module improved student attitudes, but there was no difference in the magnitude of this shift among the different demographic groups we analyzed, nor was there a difference in incoming attitudes as measured by the pre-score alone. There are two possible explanations for this.

First, the CAS, which focuses primarily on attitudes toward the practice of computing itself, may not capture attitudes about belonging and identity as someone who does computer science, and these factors may be the ones that better explain demographic differences in attitudes. In future implementations of the course we plan to include assessments that measure these other components of student attitude.

Second, it may be that negative attitudes are more strongly held in environments in which students are the minority group. In contrast to computer science, where there is a strong gender imbalance, biology majors typically have greater gender equity in their cohorts. At the institution where we collected data, only 17% of computer science majors were women; by comparison, 60% of biology majors were women (institutional research, 2017/2018 school year), and in the student responses we analyzed, 75% of the students were women. This may create a more welcoming environment for female students, and suggests that teaching computing in the context of biology may be a way to better reach underrepresented students.

In the future, we hope to assess the impact of this module on other student outcomes, including content knowledge and understanding in bioinformatics, as well as potential gains in other related areas, like microbiology and evolution. Additionally, we plan to explore how this module, or any introductory bioinformatics module, could be improved in ways that lead to an even greater shift in student attitudes toward computing. This module incorporates a tutorial to walk students through the mechanics of command-line work, but there could be other potential learning activities or self-reflections focused on students’ self-efficacy and capacity for growth that might improve outcomes, especially for populations underrepresented in STEM and bioinformatics.

## Data Availability Statement

The original contributions presented in the study are included in the Supplementary Material and via figshare, further inquiries can be directed to the corresponding author.

## Ethics Statement

The studies involving human participants were reviewed and approved by UC San Diego Institutional Review Board. Written informed consent for participation was not required for this study in accordance with the national legislation and the institutional requirements.

## Author Contributions

KP developed this course module, wrote instructional materials for it, collected assessment data and provided guidance on its analysis and wrote the manuscript. RX analyzed the assessment data and wrote the manuscript. Both authors contributed to the article and approved the submitted version.

## Conflict of Interest

The authors declare that the research was conducted in the absence of any commercial or financial relationships that could be construed as a potential conflict of interest.

## Footnotes

1 https://www.cygwin.com/

2 https://docs.conda.io/

3 https://bioconda.github.io/

4 https://www.ncbi.nlm.nih.gov

## Supplementary Material

There several supplementary files accompanying this manuscript, listed below.

1. Resequencing Manual. This manual for students contains step-by-step directions for both the wet and dry lab components of this module, a detailed list of materials, and background information.
2. Illumina Sequencing slides. Lecture slides with active learning activities that can be incorporated into lectures that prepare students for this laboratory module. All images in the slides were generated by the author.
3. Illumina Library Prep slides. Lecture slides with active learning activities that can be incorporated into lectures that prepare students for this laboratory module. All images in the slides were generated by the author.
4. Troubleshooting Guide.
5. Sample Dataset Overview & Expected Results.
6. Sample Dataset, available via figshare 10.6084/m9.figshare.13426889.
