# Supplementary material for: Resequencing of Microbial Isolates: A Lab Module to Introduce Novices to Command-Line Bioinformatics
Source: Front Microbiol. 2021 Mar 16;12:578859. doi: 10.3389/fmicb.2021.578859 (PMC8008064; doi:10.3389/fmicb.2021.578859)
Supplement: Supplementary file 1 [file Data_Sheet_1.pdf]

Preparing DNA for Genome Sequencing

Objective

How does the strain you've isolated compare other strains of the same species? What mutations make it different? Do those mutations explain any differences in phenotype that you see? Remember, mutations are random - where and when they occur is determined by chance, so it is very likely that your strain may have multiple mutations when you compare it to a reference, and your challenge will be to find out which ones explain the phenotypic differences.

In order to sequence the genome of your microbes, you will extract the genomic DNA from your bacteria, and then you will prepare it for next-generation Illumina sequencing. This process, called library preparation, is a foundational method in contemporary biology - researchers in almost all fields of biology, including microbiology, routinely rely on next generation sequencing to better understand, diagnose, or treat the organisms and cell populations they study.

Note: The reagent volumes indicated below are per strain that is being prepared. In the materials, the volume listed indicates the exact amount added in each step, however, if reagents are being pre-aliquoted per student or per group, a little bit of extra volume should be provided in the aliquots to allow for pipetting errors.

Session 1. Inoculate culture with your selected colonies (~20 minutes)

Here you will inoculate media with an isolate of your chosen strain to make a fresh culture. While it is sometimes possible to extract DNA directly from colonies, you can usually get a much higher yield if you amplify the cells by preparing a fresh culture first.

Materials

an agar plate containing a well-isolated colony of your microbe
LB or other appropriate media (~10 mL)
2 large (13 mm diameter) test tubes

Protocol

1. Label the test tubes. One should be a blank, and the other should indicate the colony or strain you have chosen. Be sure to record the details of your selection in your notebook.

2. Using sterile technique, aliquot 4 mL media to each tube, making sure to check that media is free of microbial growth beforehand. (If media has been pre-aliquoted, simply take 1 tube with 4 mL media).

3. With sterile technique, use your inoculating loop (be sure to EtOH sterilize handle, or use disposable inoculating loop) to transfer a small amount of cells from one colony to one of your tubes. Repeat for the other colony. Don't add anything cells to the 'blank' tube.

4. Instructors will store tubes at 4°C overnight then transfer to shaking incubator, at an appropriate temperature for the microbe being studied, approximately 12-16 hours before the next lab.

© Katherine Petrie, 2020 | Do not copy or distribute without permission

Session 2. Make freezer stock of your microbe isolate (optional, ~10 minutes)

In case the results end up showing that you discovered an interesting strain, you can make a freezer stock to preserve your microbe. You will add glycerol, a cryoprotectant, to your cells, which reduces the formation of ice that would kill the bacteria.

Materials

80% glycerol (sterile) (200 µL)
cryotube (sterile)

Protocol

1. Label the cryotube with your section initials, the microbe's full name from last time, and today's date. Vortex your overnight culture to thoroughly resuspend your microbe.

2. Using sterile technique, aliquot 200 µL of 80% glycerol to the cryotube.

3. Using sterile technique, add 800 µL of overnight culture to the cryotube, and pipette slowly up and down at least 5 times to thoroughly mix.

4. Tubes will be stored at -80°C to archive the clones until we get sequencing results.

© Katherine Petrie, 2020 | Do not copy or distribute without permission

Session 2. Extract genomic DNA from your microbe isolate (~ 3 hours)

Here you will use components from a commercial kit that is designed to extract DNA from cells. The kit we use is called the DNeasy UltraClean Microbial kit from a company called Qiagen. You will first concentrate the cells by centrifuging them (spinning the culture at high speed so the cells settle to the bottom of the media). You will then resuspend the cells in a small volume of liquid that contains detergent and little beads which, when shaken, will lyse the cells (break open their membranes). The remaining solution of burst cells will contain the genomic DNA you are interested in, but it will also contain RNA, proteins, and other molecules - dead cell guts of varying sizes, basically. You can centrifuge to spin down the big stuff, but you'll have to add a special solution to the supernatant to precipitate the soluble proteins and other smaller cell junk. Finally, you will combine the protein-free solution with a salty buffer that will help the DNA bind to a special silica membrane filter that is affixed in the bottom of a spin column. Once the DNA binds, you can wash it with an ethanol-based solution that will remove other contaminants, and finally, you can elute (release) the DNA from the silica membrane with water.

Important notes

safety point: Centrifugation! These guys spin really really really fast. Always make sure your tubes are properly balanced (have an instructor check the first time you use it) and that the lid is properly attached. Never try to open while still spinning, and learn where the off/cancel switches are so you can shut down the centrifuge if you hear any problems. The centrifuges we use are designed for microtubes - larger centrifuges have even more rules.

safety point: some of the kit solutions have ingredients that are dangerous to mix with other substances, especially bleach). Please follow all waste disposal guidelines as described by your instructor.

Materials

(all materials except MilliQ water and original culture are from QIAGEN DNeasy Ultra Clean Microbial Kit, 12224)
2 mL collection tube (2)
overnight culture of your isolate in LB
PowerBead Solution (300 µL)
PowerBead Tube (1)
Solution SL ("**S**DS **L**ysis solution") (50 µL)
Solution IRS ("**I**nhibitor **R**emoval **S**olution") (100 µL)
Solution SB ("**S**alty **B**inding solution)" (900 µL)
MB Spin Column (silica MemBrane column) (1)
Solution CB (300 µL)
MilliQ H2O (50 µL)

Protocol (Adapted from QIAGEN DNeasy Ultra Clean Microbial kit)

© Katherine Petrie, 2020 | Do not copy or distribute without permission

1. Vortex your **overnight culture** to make sure it is resuspended. Using sterile technique, transfer **1.8 mL** of **overnight culture** to a 2 mL collection tube. After this point, you do not need to work with the Bunsen burner on, but do try to avoid contaminating your sample.

2. Make a balance with the tube from the other members of your group (or put 1.8 mL H_2O in a 2 mL centrifuge tube) and centrifuge at **10,000 x g** for **30 seconds** to pellet the cells. Note: 10,000 x g (gravity) is NOT the same as 10,000 rpm. The conversion depends on the centrifugal force our tubes experience, which in turn, depends on the size of the rotor (spinny part) of our centrifuge. For the Eppendorf Mini Spin Plus 5453, 10,000 x g corresponds to 12,210 rpm. Round this to 12.2 x 1000 rpm when you are setting the centrifuge.

3. Completely remove the supernatant with a pipette tip, taking care not to disrupt the cells. Transfer the supernatant to a microfuge tube, and then dispose of it as instructed. The remainder of the waste generated should also be disposed of as instructed.

4. Resuspend the pellet in **300 µL** of **PowerBead** Solution, and gently vortex to mix.

5. **Transfer the resuspended cell solution** (all of it) to a **PowerBead Tube**.

6. Add **50 µL** of Solution **SL** ("SDS Lysis") to the PowerBead Tube, and cap it securely.

7. Secure PowerBead tubes horizontally in the vortex adaptor, located on the side bench. Wait for other groups so that the adaptor is full before starting. Vortex at **maximum speed** for **10 minutes.** This creates the combined chemical and mechanical conditions that bust open the cells.

8. Transfer the PowerBead Tubes to the centrifuge (make sure they fit and can spin freely), and spin at **10,000 x g** for **30 seconds**. This will send large cell debris (cell guts) to the bottom of the tube, while DNA remains in the supernatant.

9. **Transfer the supernatant** to a clean 2 mL Collection tube. You should expect 300-350 µL of supernatant. Take care to avoid the pellet.

10. To the new tube where you put the supernatant, add **100 µL** of solution **IRS** ("Inhibitor Removal Solution") and **vortex** for **5 seconds**. This solution contains a reagent that will precipitate protein and other non-DNA material, which could reduce DNA purity and inhibit downstream applications that use your DNA.

11. Incubate at **4°C** for **5 minutes.**

12. Centrifuge at **10,000 x g** for **1 minute**.

© Katherine Petrie, 2020 | Do not copy or distribute without permission

13. Taking care to avoid the pellet, **transfer the supernatant** to a clean 2 mL Collection Tube. You can expect about 450 µL of supernatant, but don't get greedy, it's better to take less than that than to contaminate your sample with protein and other cell crud.

14. Add **900 µL** Solution **SB** ("Salty Binding" solution) to the tube where you just put the supernatant, and **vortex** for **5 seconds**.

15. Load about **700 µL** of this mixture into an **MB Spin Column** (this should be supplied inserted into a collection tube already, but if it's not, insert it into one). Centrifuge at **10,000 x g** for **30 seconds.** Transfer the flow through to a 1.5 mL microfuge tube (discard of this waste as directed later). **Repeat in batches** to load the remaining supernatant onto the same MB Spin column, discarding (as directed) all of the flow through. Each sample will require 2-3 loads. Now, your DNA is stuck to the silica membrane in the Spin Column.

16. Making sure the collection tube is empty, add **300 µL** of solution **CB** ("Cleaning Buffer") to the spin column. Centrifuge at **10,000 x g** for **30 seconds.**

17. Remove the flow through from the collection tube (discard as directed), and centrifuge **again** at **10,000 x g** for **1 minute** This essential step makes sure that all residual cleaning buffer is removed from your DNA.

18. Place the MB Spin Column in a brand new 2 mL Collection Tube. Be careful not to get any of the CB liquid from the old tube on the membrane when you transfer the column.

19. Add **50 µL** of **milliQ H2O** (get a brand new bottle!) to the center of the white filter membrane, taking care not to actually touch it (you don't want to poke a hole - you should drop the solution in from about 2-3 cm above the filter: use your medium size pipettor, and steady the barrel with your other hand). Centrifuge at **10,000 x g** for **30 seconds.**

20. Discard the MB Spin Column, and cap the tube. Store DNA at -20°C.

© Katherine Petrie, 2020 | Do not copy or distribute without permission

Session 3. Quantitate genomic DNA with fluorescence (~ 1 hour)

To accurately measure the DNA concentration, we will use a fluorescent dye which specifically binds to double-stranded DNA. You will first calibrate the fluorescence detector, called a Qubit, by measuring how much fluorescence is emitted when you add dye to DNA of known concentrations. The machine will use these standards to estimate the concentration of your DNA when you measure its fluorescence.

Important notes

safety point: Qubit nanodrop HS reagent! This substance contains DMSO and is known to bind DNA, and is therefore potentially hazardous. Be certain you are using PPE and dispose of any used reagent as directed

Materials

genomic DNA sample that you will sequence
Qubit fluorimeter (ThermoFisher)
working stock of Qubit dsDNA HS reagent (diluted 1:200 in buffer per manufacture instructions, ThermoFisher, part of Q32851 or Q32854 Kit)
Qubit dsDNA HS Standard #1, part of Q32851 or Q32854 Kit
Qubit dsDNA HS Standard #2, part of Q32851 or Q32854 Kit
Qubit assay tubes (ThermoFisher Q32856 or Axygen PCR-050C tubes)
MilliQ H_2O

Protocol (Adapted from Qubit dsDNA HS Assay Kit Manual)

1. Aliquot 190 µL of working stock of Qubit dsDNA HS reagent to 3 assay tubes. (Labeled on the lid with ‘1’, ‘2’, or ‘test’). To the tube labeled ‘1’, add 10 µL of Qubit dsDNA HS standard #1. To the tube labeled ‘2’, add 10 µL of Qubit dsDNA HS standard #2. To the tube labeled test, add **8 µL H_2O**, then add **2 µL of your genomic DNA** to the same tube.

2. Mix by vortexing 2-3 seconds, then quickly centrifuge to collect all liquid at the bottom of the tube.

3. Allow tubes to incubate at room temperature at least 2 minutes. Do not hold the tubes in your hand, as the temperature will affect the measurement. Use the samples within 3 hours.

4. Take your sample a qubit fluorimeter. Select ‘dsDNA High Sensitivity’ as the assay time, then follow the directions to ‘Read standards’ to calibrate the device. Calibration once per lab session should be sufficient. Proceed to ‘Run samples’, select the sample volume you added (2 µL), select output units (ng/µL is easiest) and follow the directions in the screen to measure the concentration of the sample. Make sure lid is closed before taking all readings.

5. The instrument displays both the concentration of your original sample (top) and the concentration as diluted in the assay tube (bottom) If you get a message that your DNA concentration is too high or too low to read, set up a new assay tube with fresh working reagent

© Katherine Petrie, 2020 | Do not copy or distribute without permission

using a dilution or larger volume of DNA, as needed. The Qubit ds DNA HS assay kit is accurate for samples ranging from 10 pg/μL to 100 ng/μL.

6. Decide if you have enough DNA for sequencing.

© Katherine Petrie, 2020 | Do not copy or distribute without permission

Session 4. Prepare DNA library for Illumina sequencing (part 1) (~ 3 hours)

Now that you have genomic DNA from your microbe of interest, you are ready to prepare it for sequencing. Even the best sequencing technology isn't able to read an entire genome in one strand, so you will break your genomic DNA - which contains many copies of the genome, since you extracted it from many cells - into smaller, more workable fragments. On the ends of each of these fragments, you will attach special short sequences called adaptors, which will help with the sequencing process. The process of fragmenting and adding the adaptors is carried out in one step, called 'tagmentation.' You will also add index adaptors that give your sample a unique tag that will help us identify them after we pool all of the class samples together (an approach called multiplexing). The collection of tagged genomic fragments is called a DNA library, so microbiologists often refer to this process as 'library prep.' We'll be using a commercial library prep kit called "Illumina DNA Prep."

Important notes

> ***science point: magnetic purification beads!*** *There will be several steps in this protocol where you will use beads for purification. These beads bind your DNA, and, in combination with a magnet, can keep it in the tube while you change out the solutions needed for library prep. When working with the beads, pay attention to whether or not the protocols call for the beads to be mixed in solution, or bound to the magnet. When they are bound to the magnet, avoid bumping them with your pipette tip - you may have to crouch down to get a closer look at where your tip is inside the tube.*
>
> ***science point: mixing by pipetting!*** *Sometimes protocols will call for mixing by pipetting up and down rather than vortexing, as it is a gentler way to mix. Whenever you mix by pipetting,* ***do not go past the first stop****. Be very careful not to suck air into your micropipette tip. While you are pipetting, always, look at the liquid in your tip to make sure you are not close to sucking air into your tip or putting bubbles into the tube. This is especially important for the TWB used in task 3 (step 13), as it has a tendency to foam. With TWB,* ***do not go past the first stop*** *when you are dispensing this stuff, as that will put air bubbles into the solution*

Materials -

genomic DNA sample that you will sequence
0.2 mL PCR tubes (3)
MilliQ water
10 mM Tris-HCl, pH 7.5-8.5
magnetic stand for 0.2 mL tubes
index adaptors - get from instructor in step 19
thermocycler
The below reagents are all from the Illumina DNA Prep kit, 20018705)
BLT (Bead-Linked Transposons) (10 µL, measured when beads are well-suspended)
TB1 (Tagmentation Buffer 1) - (10 µL)
TSB (Tagmentation Stop Buffer) - (10 µL)
TWB (Tagmentation Wash Buffer) - (300 µL)
EPM (Enhanced PCR Mix) - (25 µL)

© Katherine Petrie, 2020 | Do not copy or distribute without permission

Protocol - Tagmentation (Adapted from Nextera DNA Flex Library Preparation kit, Illumina)

1. Check with your instructor to find out directions on how to use the thermocycler where you will be incubating your tagmentation.

2. Make sure that your thermocycler is ready to go with the following program:

   - heated lid on, reaction volume 50 µL

   - 55°C 15’

   - hold at 10°C (or wait until approaches room temperature if no cooling option)

3. **Determine how much of your DNA to use.** You can add **between 2 µL and 30 µL of volume**, but will have to use the DNA concentration to calculate the best volume to add. The **ideal amount of DNA is 500 ng**, but less is OK. Reminder: (ng/µL) * (µL) = ng. **Show your calculation to your IA before moving to the next step.** If you will need to dilute your sample so that you can add at least 2 µL, you should dilute it in 10 mM Tris-HCl, pH 7.5-8.5.

4. **Label the BLT tube** so that you know it is yours (but don’t add your DNA to this until step 6).

5. Vortex the BLT tube to **resuspend the beads**, (but start slow and work your way up so that the liquid doesn’t end up on the cap of the tube). **Add 10 µL of TB1** to the BLT tube, and vortex again to mix.

6. **Add your DNA (a volume between 2 µL and 30 µL that you calculated in step 3) to the BLT tube**. If less than 30 µL of DNA is added, add MilliQ water to reach a total volume of 30 µL added. Pipette slowly up and down at least 10 times to mix, using a micropipettor set at 30 µL for the mixing

7. Place the tube in the thermocycler and run the program.

Protocol - Tagmentation clean-up (Adapted from Nextera DNA Flex Library Preparation kit, Illumina)

8. When sample has cooled, take out of thermocycler and add **10 µL TSB** to the tagmentation reaction.

9. Use a pipettor set to at least 40 µL to slowly pipette up and down 10 times to mix.

10. Create a new thermocycler program and run it with your tube.

© Katherine Petrie, 2020 | Do not copy or distribute without permission

- heated lid on, reaction volume 60 µL

- 37°C 15’

- hold at 10°C (or wait until approaches room temperature if no cooling option)

11. When has cooled, take out of thermocycler and place the tube on magnetic stand and wait until the solution is clear (may take several minutes).

12. With the tube still on the magnetic stand, taking care not to disrupt the beads, use a pipettor to **remove and discard the supernatant**.

13. Wash **two times** as follows, using a **very slow** pipetting technique to minimize TWB foaming.

- remove the tube from the stand and add **100 µL TWB** directly onto the beads

- pipette very slowly to resuspend beads

- place the tube on magnetic stand and wait until the solution is clear (may take several minutes).

- leaving the tube on the magnetic stand, carefully remove and discard supernatant

14. After the second wash, remove the plate from the stand and add another **100 µL TWB**, and pipette slowly to resuspend.

15. Close the tube and place on the magnetic stand, and leave it there until the solution is clear and you reach step 17 below.

Protocol - Index addition and amplification (Adapted from Nextera DNA Flex Library Preparation kit, Illumina)

16. Open the **EPM tube** and add **25 µL of MilliQ H_2O**. Vortex to mix and centrifuge at 280 x g for 10 seconds, then set aside for now.

17. Going back to the tagmentation tube on the magnetic stand, **remove and discard the TWB supernatant** from the beads, using a 200 µL pipette tip. Lingering foam is ok.

18. Take the tube off of the magnetic stand and immediately add **40 µL** of the **diluted EPM solution** you prepared in step 16, and immediately pipette up and down at least 5 times to resuspend the beads.

© Katherine Petrie, 2020 | Do not copy or distribute without permission

19. Figure out which index adaptors have been assigned to you, and get them from the instructor or IA. Every sample will have a unique combination of indexes. You should add **10 µL (total) of your assigned indices.** To avoid cross contamination, only open one index at a time (add one, close that tube, then add the other, and close that tube).

20. Using a pipette set to 40 µL, slowly pipette up and down 10 times to mix.

21. Create and save the following PCR protocol below on the thermocycler, but don't run it yet

- heated lid on
- 68°C 3 min
- 90°C 3 min
- Cycle (98°C 45 sec, 62°C 30 sec, 68°C 1 min) x 5 cycles

  ****use 5 cycles if your input DNA was 50-500 ng, 6 cycles if your input DNA was 25-49 ng, 8 cycles if your input DNA was 10-24 ng, and 12 cycles if your input DNA was 1-9 ng****

- 68°C 1 min
- hold at 10°C (or wait until approaches room temperature if no cooling option)

22. Place your tube in the thermocycler and run the protocol.

23. Remove tube and centrifuge at 280 x g rpm for 1 minute.

24. Store at 4°C.

© Katherine Petrie, 2020 | Do not copy or distribute without permission

Session 5. Prepare DNA library for Illumina sequencing (part 2) (~1 hour)

Here, you'll complete the final step of library prep.

Materials - make sure to share the items is bold!

your tagmented, amplified DNA library
80% molecular grade EtOH (not from squeeze bottle) (1 mL per group of 4 students)
MilliQ water
1.5 mL microfuge tube (2 per group of 4 students)
The below reagents are all from the Illumina DNA Prep kit, 20018705)
45 µL SPB (Sample Purification Beads) - exactly 45 µL in 0.2 mL PCR tube (2 tubes per group of 4 students)
15µL SPB (Sample Purification Beads) - exactly 15 µL in 0.2 mL PCR tube (2 tubes per group of 4 students)
RSB (ReSuspension Buffer) (70 µl per group of 4 students)

Protocol - Amplification clean up (remove unused dNTPs, primers) (Adapted from Nextera DNA Flex Library Preparation kit, Illumina)

1. Place tube with amplified library on magnetic stand and wait until liquid is clear.

2. While the tube is still on the stand, transfer 45 µL of the liquid into new, empty, 0.2 mL PCR tube, taking care to avoid the beads. Set this new tube aside for now (do not place it on the magnetic stand), and discard the one that now only contains beads.

3. Vortex the **45 µL tube of SPB** to resuspend the beads, then add **40 µL MilliQ water**.

4. Vortex the diluted SPB thoroughly, then also pipette up and down at least 10 times to mix. **Pipette slowly**, as this solution is viscous. Transfer **all 85 µL** into the tube with your amplified library.

5. Pipette up and down 10 times to mix the diluted SPB with your DNA.

6. Incubate at room temperature 5 minutes

7. Place on magnetic stand and wait for solution to clear. In the meantime, vortex the **15 µL tube of SBP** to resuspend the beads.

8. While the tube with your DNA is still on the stand, transfer **all liquid from it** (~125 µL) to the **15 µL tube of SBP**, and pipette 10 times to mix. Discard the old tube.

9. Incubate at room temperature 5 minutes.

10. Place on magnetic stand until solution is clear.

© Katherine Petrie, 2020 | Do not copy or distribute without permission

11. With the tube still on the magnet, remove and discard supernatant, taking care not to disturb the beads.

12. Wash the beads two times as follows.

- with the tube on the magnet, add **200 µL** fresh **80% ethanol** without mixing.

- incubate on the stand 30 seconds.

- without disturbing beads, remove and discard supernatant

13. With the tube still on the stand, use 20 µL pipette tip to remove residual ethanol from tube.

14. Air dry the tube on the stand 5 minutes.

15. Remove from magnetic stand and add **32 µL RSB** to the beads.

16. Pipette to resuspend.

17. Incubate at room temperature 2 minutes.

18. Place on magnetic stand and wait until liquid is clear.

19. Transfer **30 µL supernatant** to a new 1.5 mL microfuge tube. leaving beads behind.

20. Store in -20 freezer up to 30 days.

© Katherine Petrie, 2020 | Do not copy or distribute without permission

Session 5. Quantitate genomic DNA with fluorescence (~1 hour)

Here, we'll quantitate how much tagged DNA we have. It is important to know the concentration of DNA in everyone's sample, so that we can pool equal amounts of DNA for multiplex sequencing.

Important notes

safety point: Qubit nanodrop HS reagent! This substance contains DMSO and is known to bind DNA. No data is known about its mutagenicity. Be certain you are using PPE and dispose of any used reagent as directed (NOT in the trash and NOT in biohazard waste)

Materials

prepared DNA library for your isolate
tube of 1X Qubit dsDNA HS reagent (exactly 190 µL per 0.5 mL qubit tube, tubes located under foil, 2 per group of 4 students, plus ~5 mL surplus per room for extra measurements)
MilliQ H20
1.5 mL microfuge tube for dilution, if needed.

Protocol

Follow the directions outlined in Session 3 to quantitate your DNA library.

(whenever ready) Prepare and submit aliquot of library for Illumina sequencing (~30 minutes, depending on class size)

Protocol

1. Follow the instructor directions to add your sample to the class pool.

© Katherine Petrie, 2020 | Do not copy or distribute without permission

Session 6. Unix Shell Tutorial (~ 3 hours)

Hardware Required

computer with Unix-based command line interface

Introduction

Most bioinformatics is carried out "at the command line". This means that instead of using a shiny GUI (graphical user interface) where we point and click and see things happen in living color, you work in a plain text-based window, and interact by typing in commands and reading text-based files. If you haven't done this before, it can be frustrating at first, as it's sensitive to typos and formatting, but once you get used to it, it will feel faster than working in a GUI. If you find it helps make the learning curve less painful to pretend you're Angelina Jolie or Johnny Lee Miller from the 1995 cult classic *Hackers*, then by all means, don't let us stop you.

The Shell

A 'shell' is a program that processes the commands you input, and returns output to the screen. On Macs, a program called Terminal runs the shell for you. Unix and Linux computers can also run shells, also called Terminals. If you have a PC, your best bet is to install Cygwin or some other emulator that will let your PC pretend it's a mac or a Unix machine.

Start terminal by finding and starting your terminal program. Terminal will launch with a blank window that you can type in.

This new window should have your user name and the word "–bash" written at the top. Bash stands for 'Bourne Again SHell', and is the command interpreter program for Unix and mac (which is based on Unix) operating systems. Bash has its own command language, also called bash, some features of which we'll go through below, and it can run programs in other computer languages, like python or R.

You can work in the shell in several modes:

> In interactive mode: you type a command, hit return, and see the output, then can type other commands to do stuff with the output (this is what we'll be using).
>
> By running scripts: scripts are simple programs. you write a series of commands in a short text file, save that text file as a 'script', then run the whole script with a single command
>
> With job control: many different scripts, sometimes from different users, can be running at the same time and sharing the computer's resources

It's important to remember that when you are working in the shell, you are accessing and creating documents that live on your hard drive just like you would if you were navigating through folders with your mouse. Files are arranged hierarchically in nested folders, and you can see this hierarchy indicated in a files' 'path' which describes its precise location. *Note: if you delete a file from the shell, it will disappear from your hard drive too, and there is no 'trash can' or control z for the shell, so be careful!*

© Katherine Petrie, 2020 | Do not copy or distribute without permission

Survival Unix (--bash shell commands)

The following should serve as a reference for the most commonly used bash commands and tricks. Move on to the next section for an introduction to using them. In the commands below, the command itself is in black text, and user-specific files and destinations you can input are in blue.

`pwd` print working directory- outputs the path to the current directory

`ls` list- lists the contents of the current directory and helpful to figure out where you are

`ls -l` list –long- list with the 'long' flag option, which give the size of the files and their permissions as well as some additional information

`ls -lh` list with the 'long human' option, which makes the file sizes a bit easier to read

`cd ..` change directory to one level up

`cd /path/to/directory` change directory to the one specified with the path

`cd ~/` change directory to the home directory (a user's top level directory)

`cat filename` opens named file

> ****A NOTE ON FILENAMES: commands that refer to 'plain' objects with filename or directory name only, will only work on files in the current directory. If you want to work on files located somewhere else, you have to tell the shell where they are by typing the full path to them*

`head -X filename` displays first X lines of named file. Without a flag, the default is 10 lines.

`tail -X filename` displays last X lines of named file. Without a flag, the default is 10 lines.

`mkdir name` make new, empty directory with name you input

`rm filename` remove the named file

`rm -r directoryname` remove recursively the named directory and all its contents

`mv filename /path/to/destination` moves named file to a specified destination

`cp filename /path/to/destination` copies named file and moves it to destination

© Katherine Petrie, 2020 | Do not copy or distribute without permission

The tutorial: Making folders and files, running programs in Unix.

In this very brief activity, you'll practice making folders and files, and running simple operations on them. Like above, the command itself is in black text, and user-specific files and destinations you can input are in blue. In order to get the most from this tutorial, it is essential that you work through it on your own, and try to figure out any errors that occur. It's totally OK to ask for help, but don't just follow along while someone else demonstrates - you really have to try it independently.

Start the shell/terminal, and open the preferences for it (typically under 'Terminal' at the top left of the screen). Click on profiles to set up your viewing options. You can choose whichever color scheme floats your boat, but you should check the option 'Blink cursor' (in the Text tab). Start a new terminal window to get these changes to go into effect.

Once you have a terminal window of your preferred color scheme with a blinking cursor open, find out what your current location is with 'list':

```
ls
```

Hit enter after typing the command, and you should see a listing of all the directories at your current location. Navigate ('change directory') to the desktop.

```
cd Desktop
```

You can probably just type "Desk" and then "tab-complete" (see below) to fill out the rest.

> ****KEY UNIX TIP: TAB COMPLETE –When you are specifying files or directories in your current working directory, you only have to type the first few letters, than you can hit 'tab' and it will autocomplete the rest of the name (if it dings or flashes, there is more than one object that starts with those letters, so add a few more characters to uniquely specify what you are looking for)*

Use 'list' (ls) again to see the contents of your desktop, then create a new folder with 'make directory.'

```
mkdir somecleverfoldernameyoupickout
```

> ****KEY UNIX TIP: FILE NAMING: Generally, naming a file something boring with no spaces or symbols or uppercase letters, like "bueller_bueller_bueller" is much better than naming it something like "Do YOOOOOOouuu speak whaAALE?" Spaces and symbols are hard for shells to interpret, and with lots of capital letters, it's easy to make mistakes.*

If you type ls again, you should see the new folder among the list of items. You should see this folder with your brilliantly chosen name show up graphically on your desktop too.

Back in terminal, make a second folder, also on the Desktop, called "california". "cd" into this California folder, and make a new folder called "san_diego". cd into the san_diego folder and make a folder called "so_cal". "cd" into the so_cal folder.

© Katherine Petrie, 2020 | Do not copy or distribute without permission

Whoops! We screwed that up. The san_diego folder should be nested INSIDE the so_cal folder, since San Diego is a city within Southern California. How do we fix it? Well, first let's try to move the "so_cal" folder into the california folder. To do this, we have to learn about paths. These are the 'addresses' for folders and files inside your computer. To see the path for our current location (the so_cal folder), you will use a command called 'print working directory'

```
pwd
```

After you hit enter, this should give you the full 'address' for the so_cal folder. You should see that it is in the san_diego folder, which is in the california folder, which is on the Desktop, which is in the your user folder....and so on. This complete path is called an "absolute path" because it contains the full address for the folder, starting from the 'top' level of the computer all the way to the name of the folder (or file). You can copy that absolute path if you'd like.

Now that we know the path for the folder, we will move it to california folder. Use the move command below. First I listed the generic command, then I listed the command with the 'source' filled in. Figure out how to fill in the destination (the california folder) and then mv the folder.

```
mv pathtosourcefolderorfile/ pathtodestinationfolder/
```

```
mv /Users/yourusername/Desktop/california/san_diego/so_cal
pathtodestinationfolder/
```

After hitting return, you should now have the so_cal folder located at the same level as the san_diego folder. Cd to the california folder and verify that this is true. Wait, how do we cd out of the san_diego folder?! You could use cd + the absolute path for where you'd like to go, but it's actually a bit easier to just type:

```
cd ..
```

The '..' is code for the 'next directory' up, which in this case is now the california folder (if our move was successful). Another address short cut is the tilde '~' which will take you to the top directory.

Finally, we can move the san_diego folder into the so_cal folder, where it belongs. Now that the folders are in the same directory as the one we are working in (the california folder), we don't have to use the full absolute paths. You can instead just type:

```
mv san_diego/ so_cal/
```

Check that everything worked, and cd into the san_diego folder.

Next, you will make a simple text folder with a little within-terminal program called nano. All of the commands we've been using so far (like mv) are really miniature little programs. Commands and programs in terminal often take the format of:

```
command -options file_to_act_on
```


© Katherine Petrie, 2020 | Do not copy or distribute without permission

Nano is a bit different, in that by just typing 'nano' we'll actually enter the program, and the terminal will change its appearance. Before we open nano, let's try finding out more about it.

`nano -man` or `nano -h` will open up the nano manual/help page, which shows us what different options are.

> ****KEY UNIX TIP: FLAGS Think of the hyphen when you specify command options as the 'flagpole' and the text after it as the option you are specifying, these options, called "flags" are used by many commands*

Go ahead and start nano, by typing nano and hitting return. The blank screen is a simple text editor. Try it out by typing a twitter-length sentence starting with "My favorite thing about working at the command line is......"

When you are done, let's save this file. Press control (that's what the ^ carrot at the bottom of the nano screen means on a mac) and the letter 'o' to write out, and enter a file name when prompted. (If you want to be neat about things, you could add a '.txt' extension to your file name. Nano should display 'wrote 1 line'. Press control + x to exit, and use list to see if your file is there.

Is our file small enough to tweet? Try using list with an option that will tell you the file size. One option is –l (for long). Try it!

```
ls -l
```

Well that gave us more information, and some numbers that look like they could be files sizes, but what the heck do they mean? Use the ls manual or google to try to find what each item means. (Hint: in some programs, the flag for the manual is not '-man' or '-h' but '--help'. For other programs, you have to type 'man' before the program name to bring up the manual. You will have to find out what works by trial and error).

You should have been able to figure out which of the numbers represent the number of bytes in your text file (A byte contains 8 bits, or binary units - the ones and zeros that are the raw data in computers). Normally, 1 byte = 1 character, but just to double check, let's try another command that will specifically count the number of characters.

```
wc -m nameofyourfile
```

'wc' is the 'word count' command, which, as you might guess, returns the number of words in a file. But with the '-m' flag, it will return the number of characters. Does it match the number of bytes?

Double check wc by taking another look at your tweet. Let's not open nano again, but try a simple command that will read text files to the screen (sometimes the screen is called 'standard out'.).

```
cat nameofyourfile
```


© Katherine Petrie, 2020 | Do not copy or distribute without permission

Did we get it right? Do the # of letters, spaces, and punctuation marks in your tweet equal what you got with word count? If your estimate is 1 character lower than what wc and ls told you, it could be due to an 'invisible' character, called a 'newline character.' These are created when you hit 'enter' or 'return' on your keyboard in the text editor. These symbols are not themselves displayed, but tell the computer to display what follows in a new line of text. If you are under 144 characters, you can tweet your response (But you don't have to).

> ****KEY UNIX TIP: HOW TO CANCEL: If you accidently open a huge file with a command like cat and just see characters flying down your screen, matrix style, or if you think a process you are running may be stuck (one clue is that your cursor stops blinking), you can press control+c to cancel the last command.*

So now you know how to navigate the folder hierarchy, make files, move things around, and find out some simple properties about your files. Could you have done all of this from the gui (=graphical user interface aka point and click)? Sure, but if you had hundreds of files to manipulate, or had to use software that doesn't exist with a GUI (aka, almost all bioinformatic software used by microbiologists), command line is your best bet.

> ****KEY UNIX TIP: RECALL PREVIOUS COMMANDS:* If you are going to be executing nearly the same command, with a small change, like if you are processing multiple files, instead of retyping, you can press up and down at the command prompt to scroll through your recent commands, then use the arrow keys and delete key to modify them.

© Katherine Petrie, 2020 | Do not copy or distribute without permission

Session 7. Inspect sequence files and filter them (~ 1 hour)

Hardware Required

computer with Unix-based command line interface

Software Required

FastQC (documentation available at http://www.bioinformatics.babraham.ac.uk/projects/fastqc/)
FastX-Toolkit (documentation available at: http://hannonlab.cshl.edu/fastx_toolkit)
breseq (requires bowtie2 and R) (documentation available at https://barricklab.org/twiki/pub/Lab/ToolsBacterialGenomeResequencing/documentation/)

Files Required

FASTQ files from high-throughput sequencing
annotated reference file in GenBank (.gbk) or gff3 format

Now let's actually take a look at your data. Open terminal and make a new directory for your data files. Make sure your FASTQ sequencing file(s) and a reference file are located in the directory.

Inspect your files

Start by inspecting your sequencing data and the FASTQ format. Use the 'head' command to look at the first few lines of one of your FASTQ files.

```
head -12 yourfile.fastq
```

What you will see is data in the FASTQ format, which consists of 4 lines per sequencing read, followed by 4 lines for the next sequencing read. (A 'read' corresponds to an individual DNA molecule in the original sample, and so each read represents the DNA from a different fragment).

- **Line 1** Starts with "@" and is the read name.

- **Line 2** The actual sequence of bases in the read.

- **Line 3** Starts with "+" and is the read name (again).

- **Line 4** is the quality string, where ASCII characters encode the quality score for each base. The quality score ranges from 0 to about 40; the higher the number, the greater the accuracy of the base call. To get the actual quality score, you need to figure out the value of the symbol, then subtract 33 (this is called 'sanger' scaling type, because it's the same scaling that people use with traditional sanger sequencing) or 64 (this is used for older sequencing data).

Answer the following in your notebook, using a table of ASCII symbols and values, which can easily be found on the internet.

© Katherine Petrie, 2020 | Do not copy or distribute without permission

What is the ascii symbol, quality score, and probability of error for the first base in the first read in your file?

Do you think your quality strings are Sanger scaled, or do they use the old, minus 64, format? (Hint: look up a table of ASCII printable characters online).

How big are your samples? Use the word count command below to answer the following. We'll be doing some processing and filtering that may remove some of the reads, so it's important to know what you started with.

```
wc -l yourfile.fastq
```

How many lines are in each file?

How many reads are in each file? (Hint, calculate this using what you know about the FASTQ format, and your answer from the previous question).

Remember, if you want to run a command similar to a previous command, you can use the up and down arrow keys to sort through your previous command, then just edit the command as needed before hitting enter.

Next let's take a closer look at the quality scores for your individual files. (On base space we could see that information for the whole run, but we want to know what your data looks like).

For this we'll use a program that actually has a GUI - so you can leave terminal aside for now. Locate the program "FastQC" on your computer, and open it. Then use File > Open it to open each of your data files.

Focus on the "per base sequence quality" How does the accuracy of these reads look? Is one direction better than the other?

Filter your files

Here you will filter your sequence files to remove low-quality reads. (It is ok if you do not get through this today).

We will clean up your files by keeping only those reads where at least 80% of the bases have a quality score of 20 or higher.

What accuracy does this cutoff correspond to?

The tool we will use is from the "FASTX-Toolkit" (from the Hannon lab), which is software you run from the command line. Make sure you are in the same directory where your ancestor and selected files are. Use the man page for the fastq_quality_filter command to figure out what flags you need, and what the missing numbers should be in the command below, then run it on each of your files (this may take

© Katherine Petrie, 2020 | Do not copy or distribute without permission

a few minutes). If you have paired-end sequence two files for each sample, you will have to run fastq_quality_filter twice, once on the forward reads file (R1) and once on the reverse reads file (R2).

```
fastq_quality_filter -flag ## -flag ## -i yourfile1.fastq -o
yourfile1_clean.fastq -Q33
```

Run fastQC on the new files. Do the quality scores look any better? If you are satisfied with your filtering, save an image of the per base sequence quality for each file, and record the following in your notebook.

How many sequences remain in each of your files?

Now your samples are ready for alignment to the reference, so we can look for mutations. We'll start that next time.

© Katherine Petrie, 2020 | Do not copy or distribute without permission

Session 7. Align sequence files to reference and identify mutations with breseq software (~2 hours)

The software you will use to carry out alignment and mutation calling is called breseq. (for Bacterial RE SEQuencing, because we are 're'sequencing our microbes and comparing them to a known reference sequence). Every time you run breseq, it will make a lot of files, so you will need to create a new directory to put those files in (but leave the actual .fastq files and reference file in the top directory).

```
mkdir cleverdirectoryname
```

Once you make a directory, you can run breseq. The basic command is below. The j flag specifies how many processor cores you want to use to work on the data. The more you use, the faster the analysis will run, but you can't use more than you have on your computer, so you will have to try to find out the number of cores you have or just leave out the entire -j option. The command below is for paired-end sequencing data (where each DNA fragment was read starting from both ends). If you have single-end data, you would only have one file to be analyzed. Be sure to replace the items in blue with the correct file and directory name.

```
breseq -j numberofprocessorcores -r reference.gbk -o cleverdirectoryname
filetobeanalyzed1.fastq filetobeanalyzed2.fastq
```

Breseq will take about 20 minutes to run, and if it is successful, the output files will be place in the directory you specified. To look at the results, navigate to that directory in finder (not in the terminal), enter the 'output' directory, and double click on the file called 'summary.html'. This will open a browser window with the results displayed in a graphical format.

Click on 'summary statistics' at the top of the screen, and take a look at the % of reads that mapped. This is the fraction of reads that the software was able to map onto the reference genome.

What is the percentage of unmapped reads for your sample?

What do you think the reads that didn't match are?

Click on 'coverage', to see how your reads are distributed throughout the genome.

Does it look like you have even coverage, or do you think there might be problem areas where there is data missing?

Next, click on 'mutation predictions' at the top of the screen. These are the mutations breseq has high confidence in - there is strong evidence that they really are mutations, and not just sequencing errors. In this screen, you should see a list of 'Predicted mutations' in green at the top, and you may see a list of 'Unassigned missing coverage evidence' in blue below this. You should focus primarily on the 'Predicted mutations' list - the unassigned missing coverage refers to regions where there wasn't enough coverage to confirm mutations.

© Katherine Petrie, 2020 | Do not copy or distribute without permission

For the 'predicted mutations', click on the blue initials next to each entry, and you'll be taken to an image of the read alignment (RA) or new junction (JC) evidence for that mutation. (RA usually refers to substitutions and short insertions or deletions, JC refers to bigger insertions and deletions. Check out the evidence for a few of your mutations, then record the following information from the main 'predicted mutations' screen:

position: the location of the mutation in the reference genome

mutation: the nature of the change

annotation: where the mutation is relative to genes, and if it's in a gene, what the effect on amino acid sequence is

gene name: the gene the mutation is in, or the genes the mutation is in between

gene function: a more useful description of what the genes actually are.

Once you've recorded that information, you can check out the "marginal mutations" link. These are mutations where breseq found some reads that were different from the reference, but didn't think the evidence was convincing enough to call a mutation. If you have marginal mutations, click on the asterix * next to the one with the highest frequency, and see if you figure out why breseq decided this isn't a real mutation.

If you want to save your files for next time, the 'output' directory should be sufficient to preserve the html results, but if you are worried, you can save the entire directory that contains output and the other files.

Finally, you should research your mutation. Compare your mutations to the ancestor. Did they arise in your experiment? Do you think they are responsible for the phenotype of your microbe? Have these mutations been seen in the literature before?

© Katherine Petrie, 2020 | Do not copy or distribute without permission
