# Supplementary material for: Resequencing of Microbial Isolates: A Lab Module to Introduce Novices to Command-Line Bioinformatics
Source: Front Microbiol. 2021 Mar 16;12:578859. doi: 10.3389/fmicb.2021.578859 (PMC8008064; doi:10.3389/fmicb.2021.578859)
Supplement: Supplementary file 2 [file Data_Sheet_2.PDF]

Microbial DNA library prep

By the end of today you will:

- be able to explain the key steps of Illumina sequencing

- be able to read Illumina sequencing output

Katherine Petrie

Microbial DNA library prep

By the end of today you will:

- be able to explain the key steps of Illumina sequencing

- be able to read Illumina sequencing output

Illumina sequencing

next generation sequencing

sequencing is the process of determining the order of the bases (A, C, G, T) in an organism's DNA

Illumina sequencing

next generation sequencing

sequencing is the process of determining the order of the bases (A, C, G, T) in an organism's DNA

Illumina sequencing is a next-generation sequencing technology that lets us sequence genomes very rapidly

Illumina sequencing

next generation sequencing

sequencing is the process of determining the order of the bases (A, C, G, T) in an organism's DNA

Illumina sequencing is a next-generation sequencing technology that lets us sequence genomes very rapidly

bacteria: 6,850,000 bp

human: 3,000,000,000 bp

↖

there is no tech that can sequence these all at once (yet)

Illumina sequencing

next generation sequencing

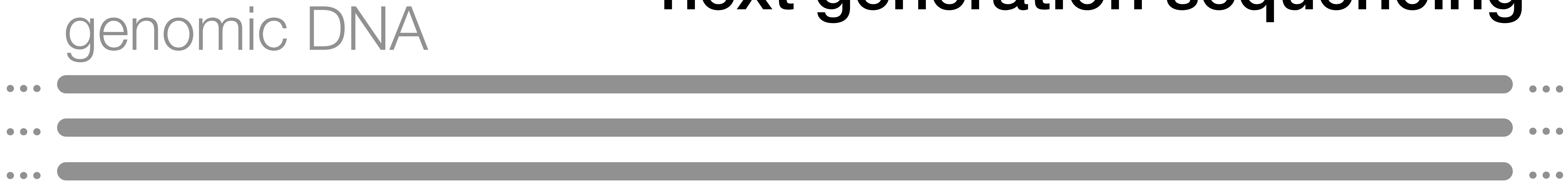

Illumina sequencing starts with many copies of genomic DNA, then goes through three steps: **fragmentation** into smaller pieces, **isolation and amplification** of each piece, and finally, **sequencing** of each piece

Illumina sequencing

fragmentation

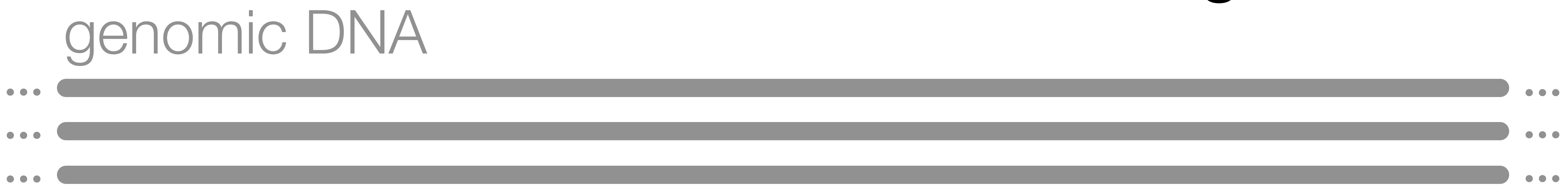

Illumina sequencing

fragmentation

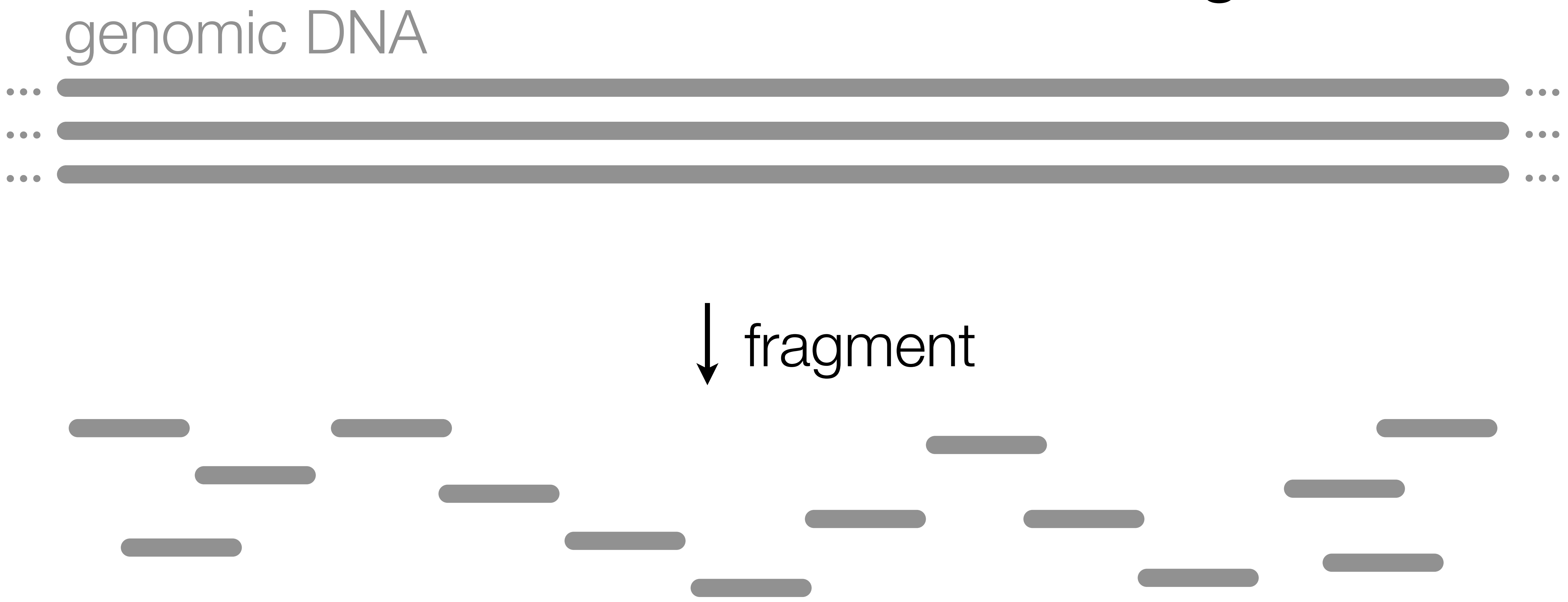

Illumina sequencing

fragmentation

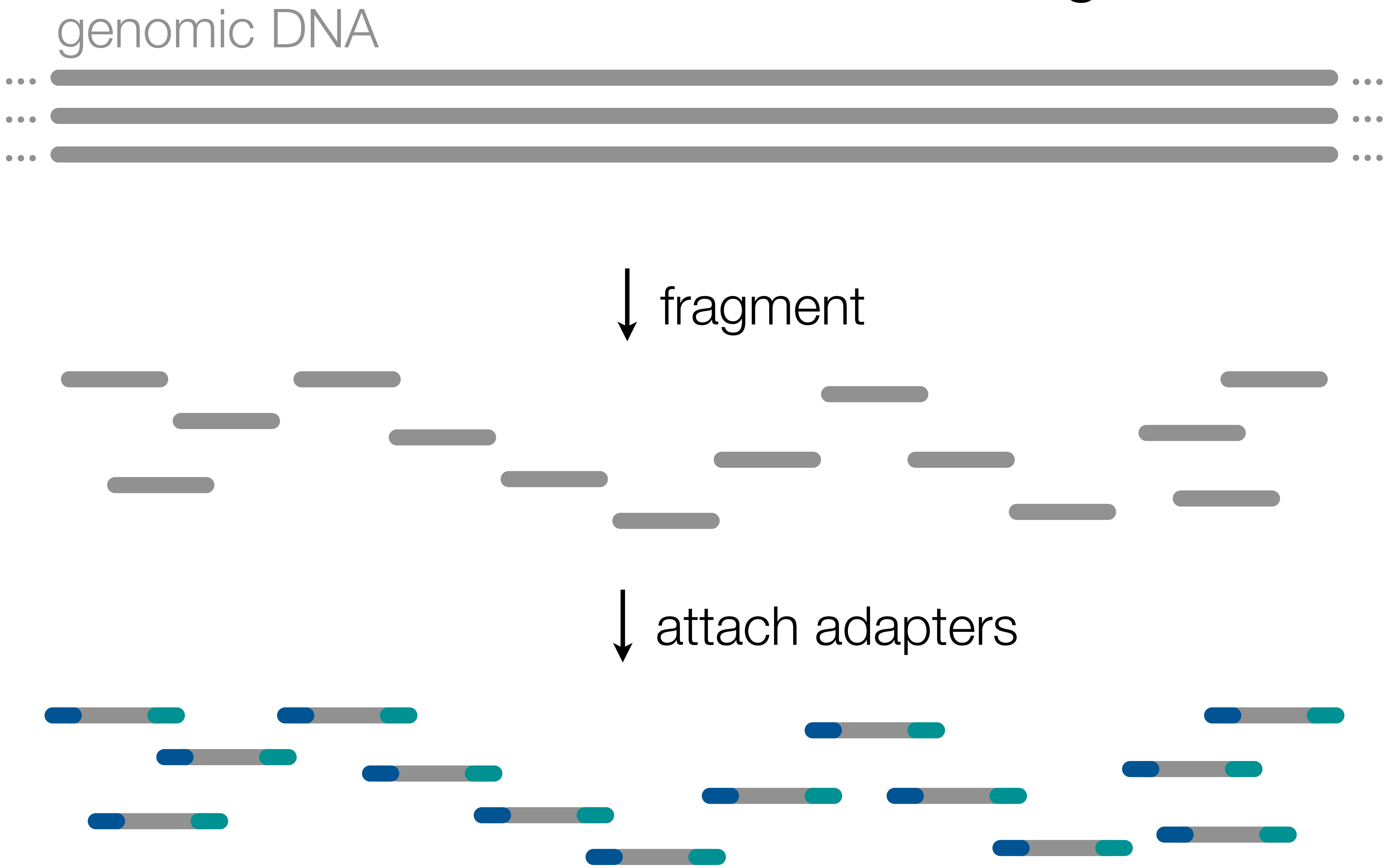

Illumina sequencing

fragmentation

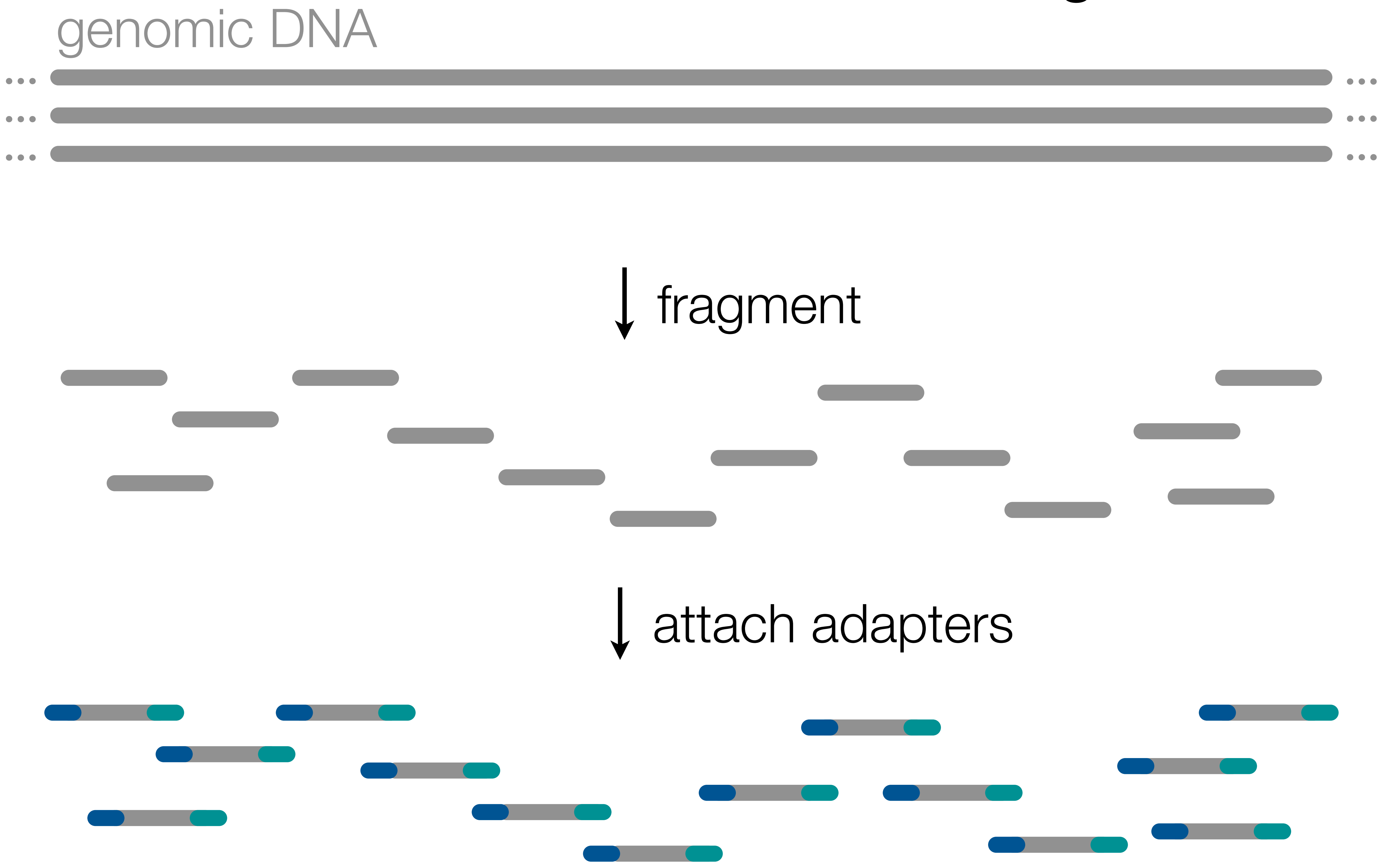

adapters = short pieces of DNA (“oligonucleotides”)

Illumina sequencing

isolating and amplifying clones

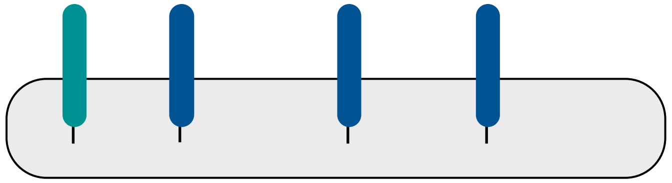

Illumina sequencing

isolating and amplifying clones

adaptors hybridize to
complementary DNA
on lawn

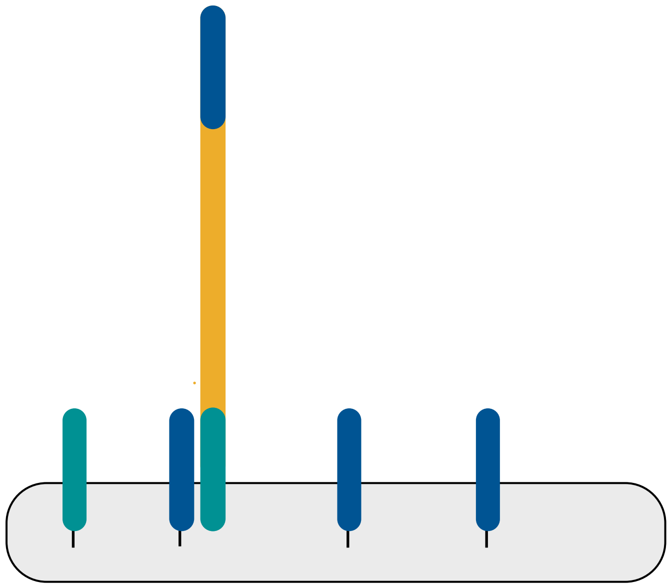

Illumina sequencing

isolating and amplifying clones

cluster generation by
bridge amplification

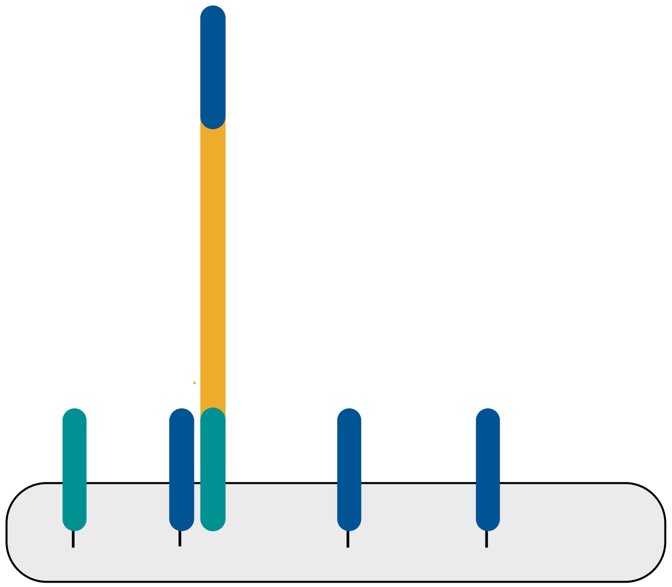

Illumina sequencing

isolating and amplifying clones

cluster generation by
bridge amplification

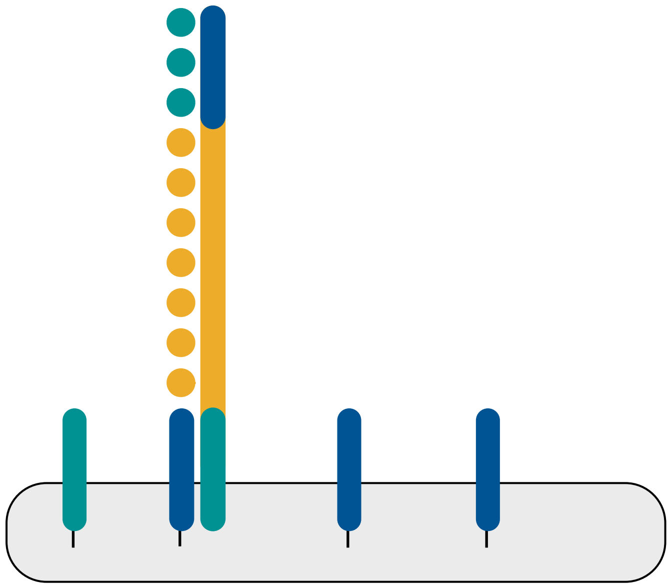

Illumina sequencing

isolating and amplifying clones

cluster generation by
bridge amplification

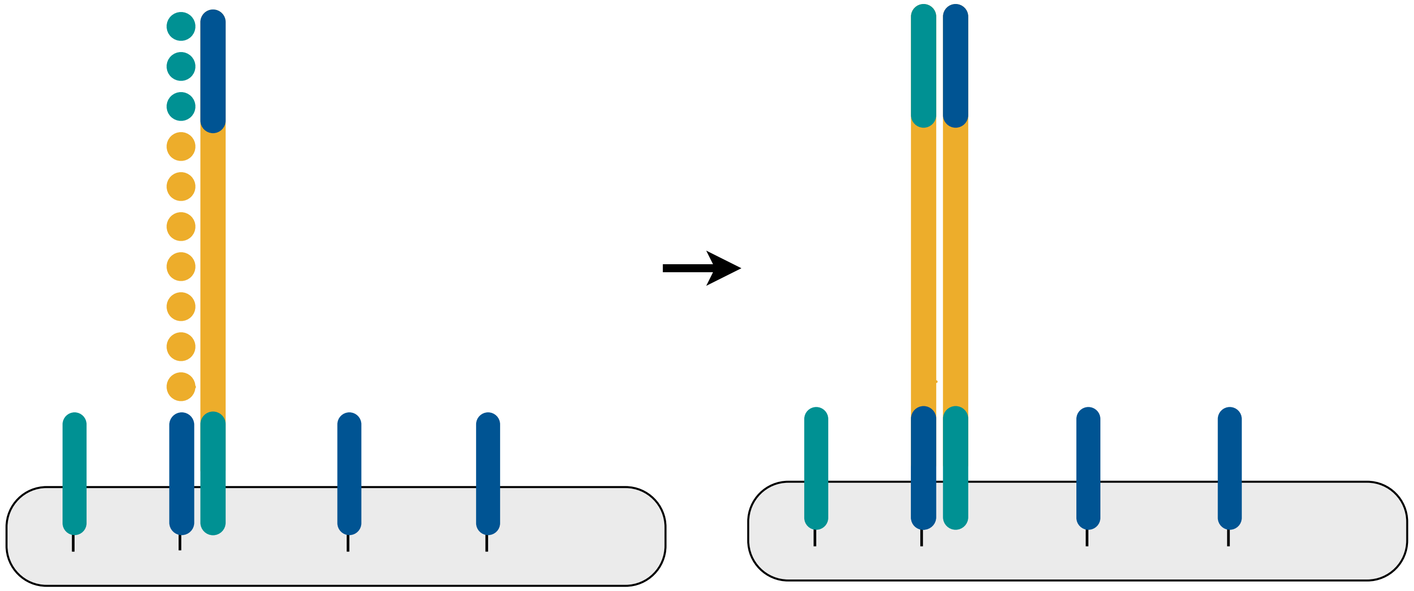

Illumina sequencing

isolating and amplifying clones

cluster generation by
bridge amplification

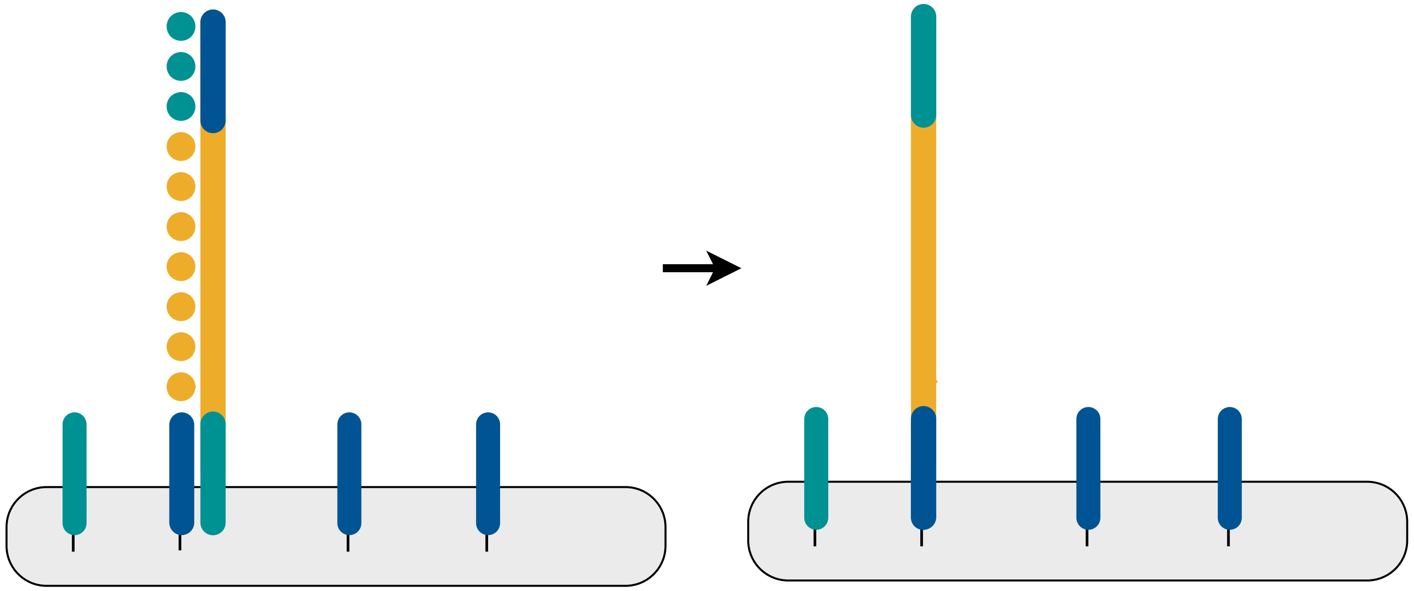

Illumina sequencing

isolating and amplifying clones

cluster generation by
bridge amplification

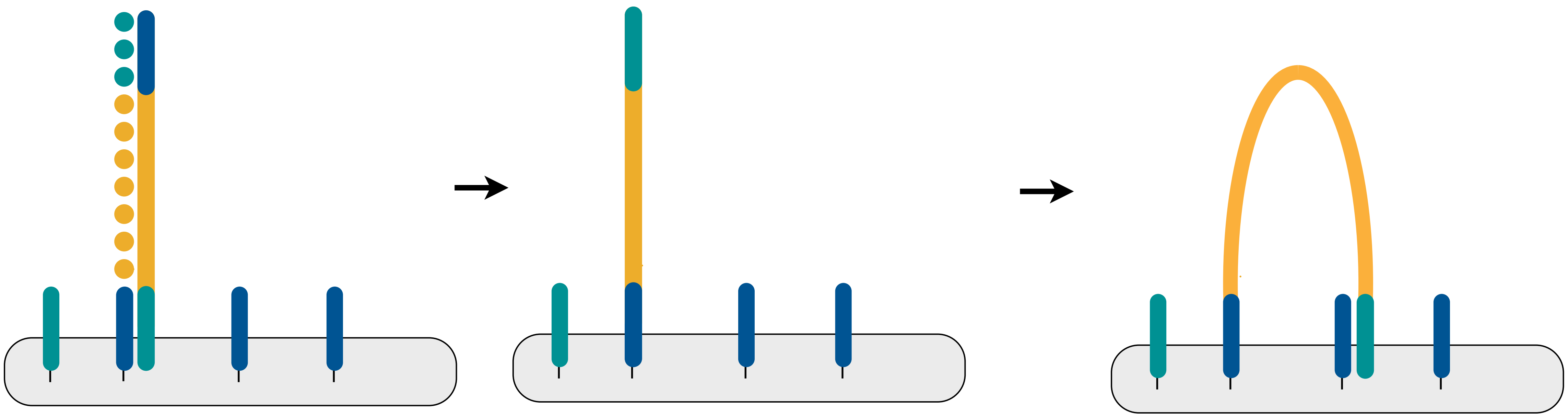

Illumina sequencing

isolating and amplifying clones

cluster generation by
bridge amplification

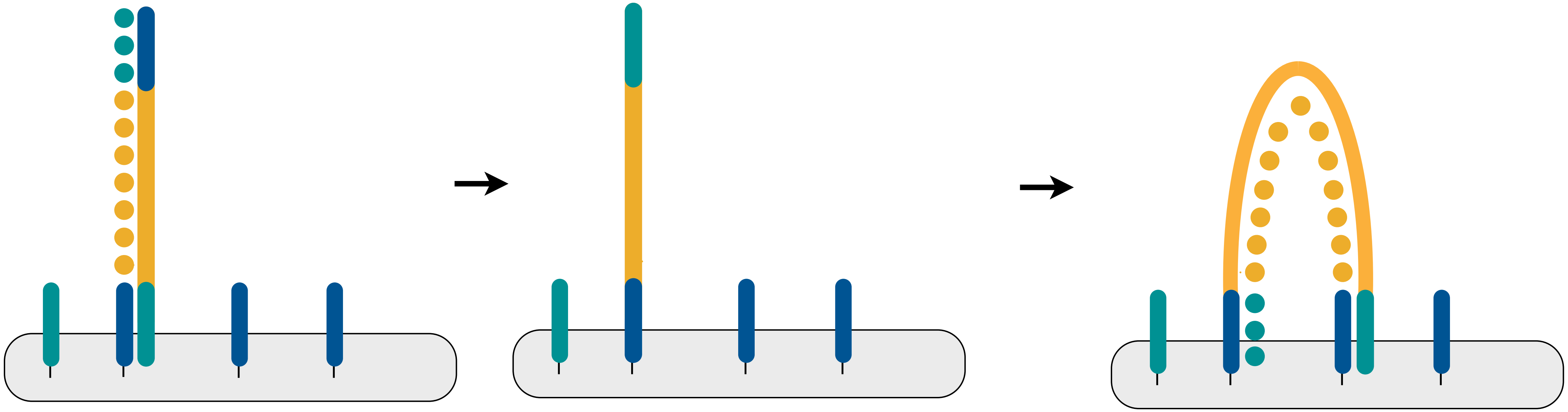

Illumina sequencing

isolating and amplifying clones

cluster generation by
bridge amplification

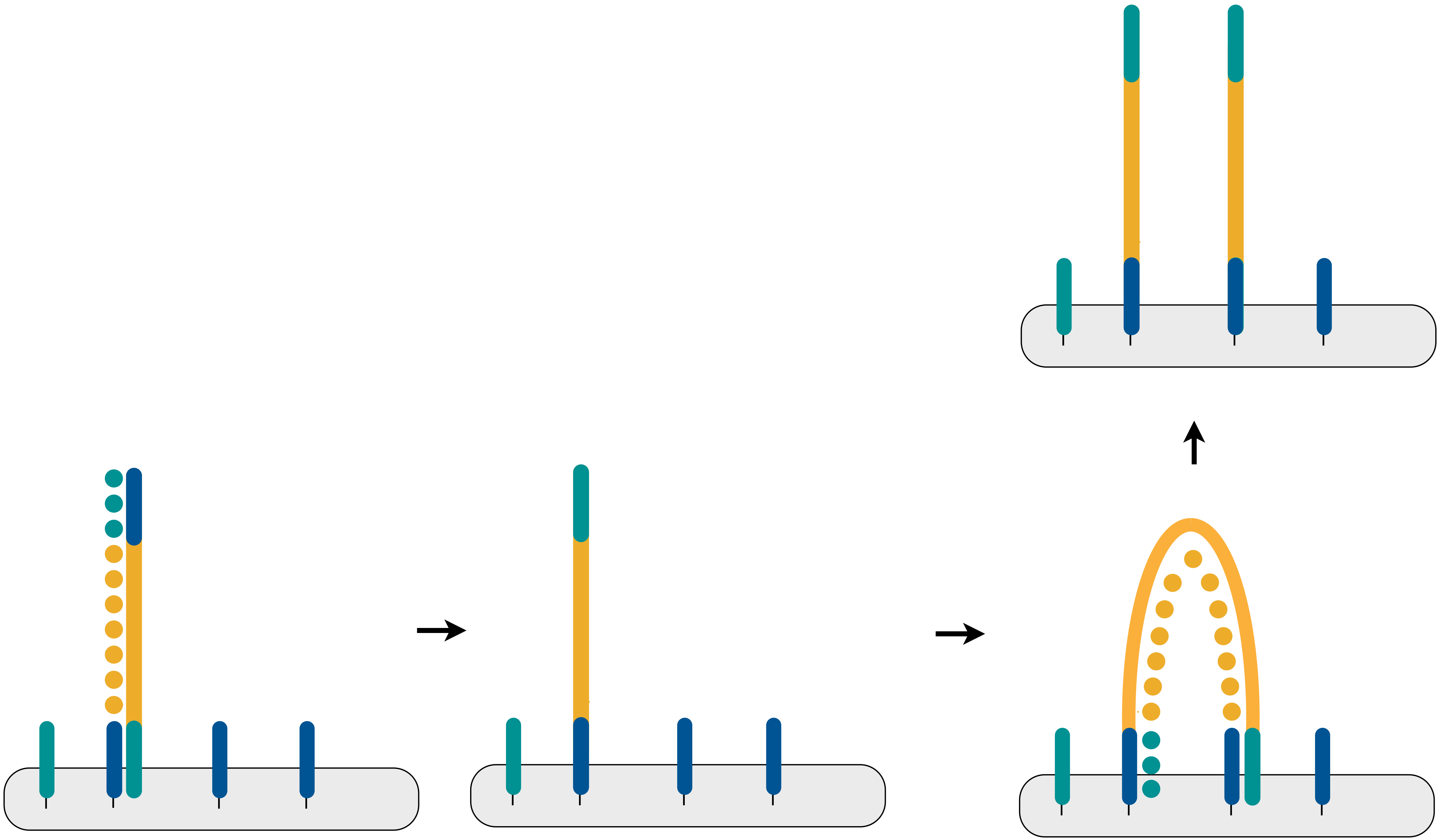

Illumina sequencing

isolating and amplifying clones

cluster generation by
bridge amplification

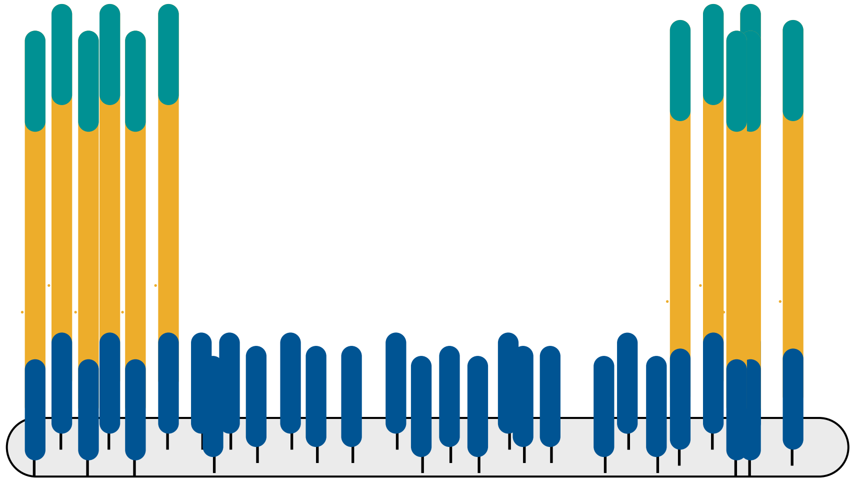

Illumina sequencing

isolating and amplifying clones

cluster generation by
bridge amplification

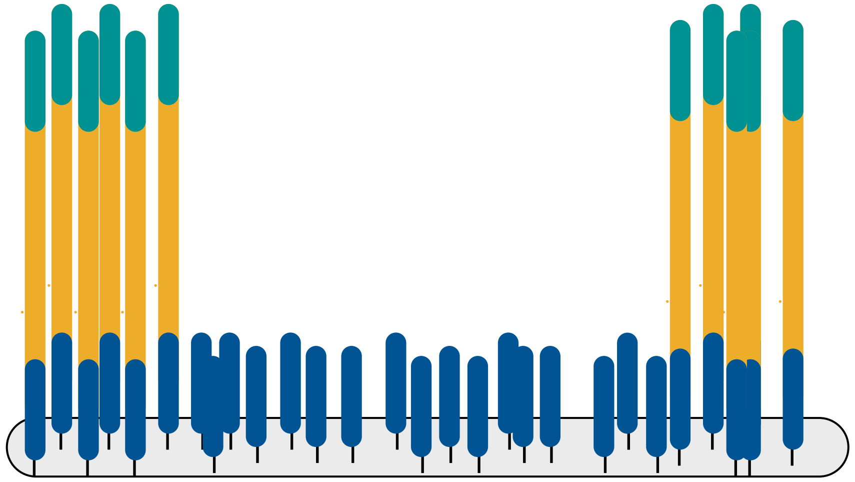

Illumina sequencing

sequencing by synthesis

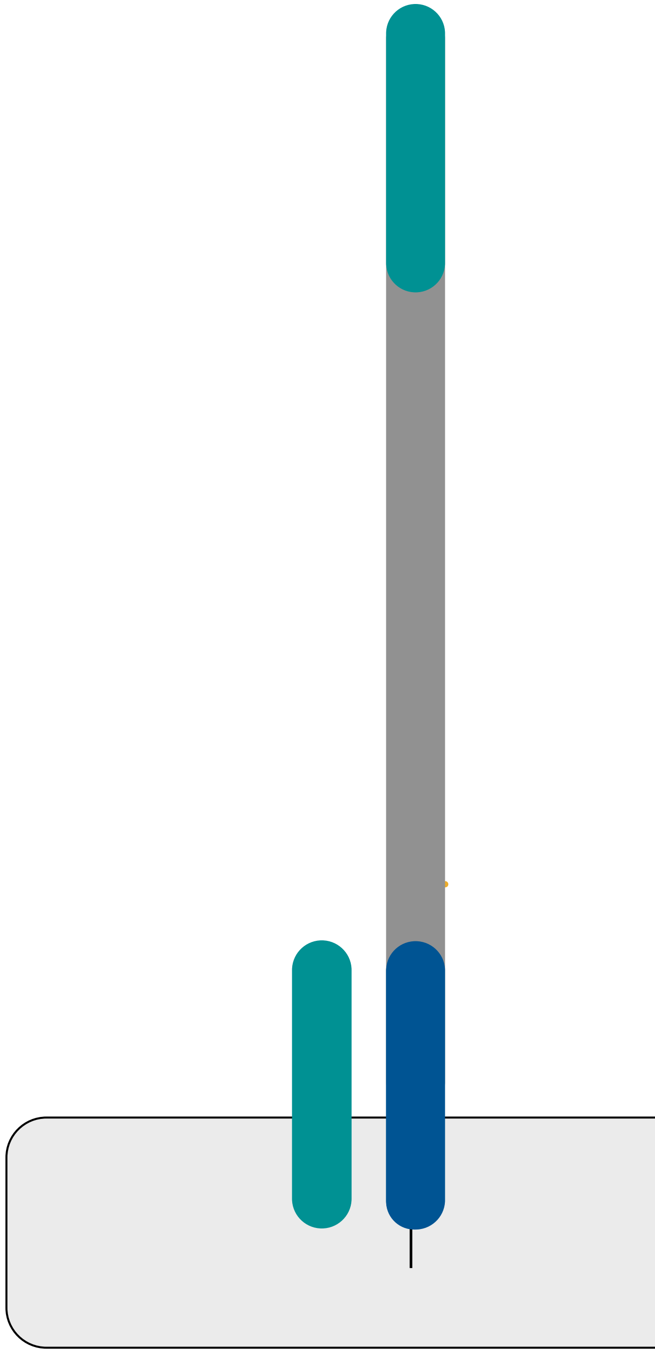

Illumina sequencing

sequencing by synthesis

polymerase
reversible terminator fluorescent dNTPS

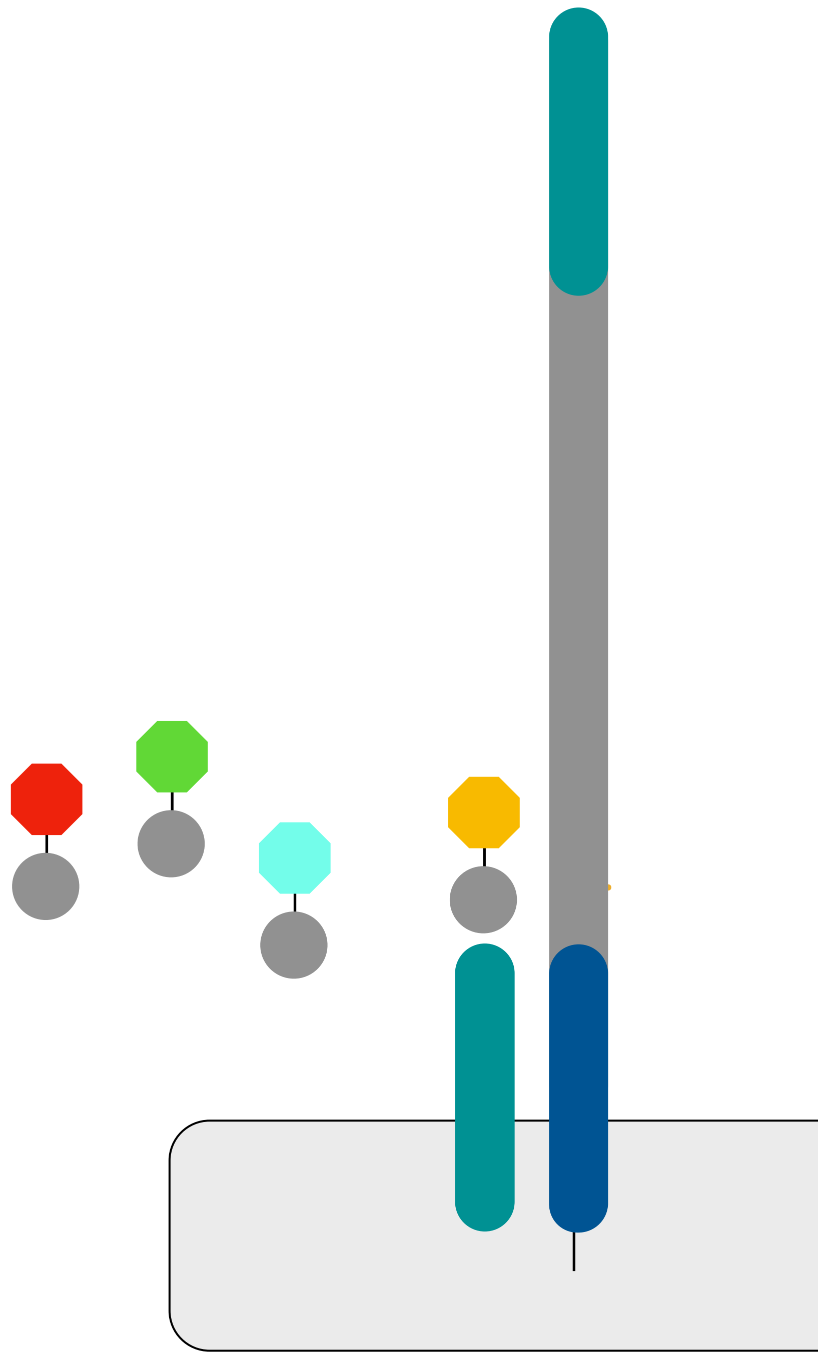

Illumina sequencing

sequencing by synthesis

polymerase
reversible terminator fluorescent dNTPS

if complementary, polymerase adds it

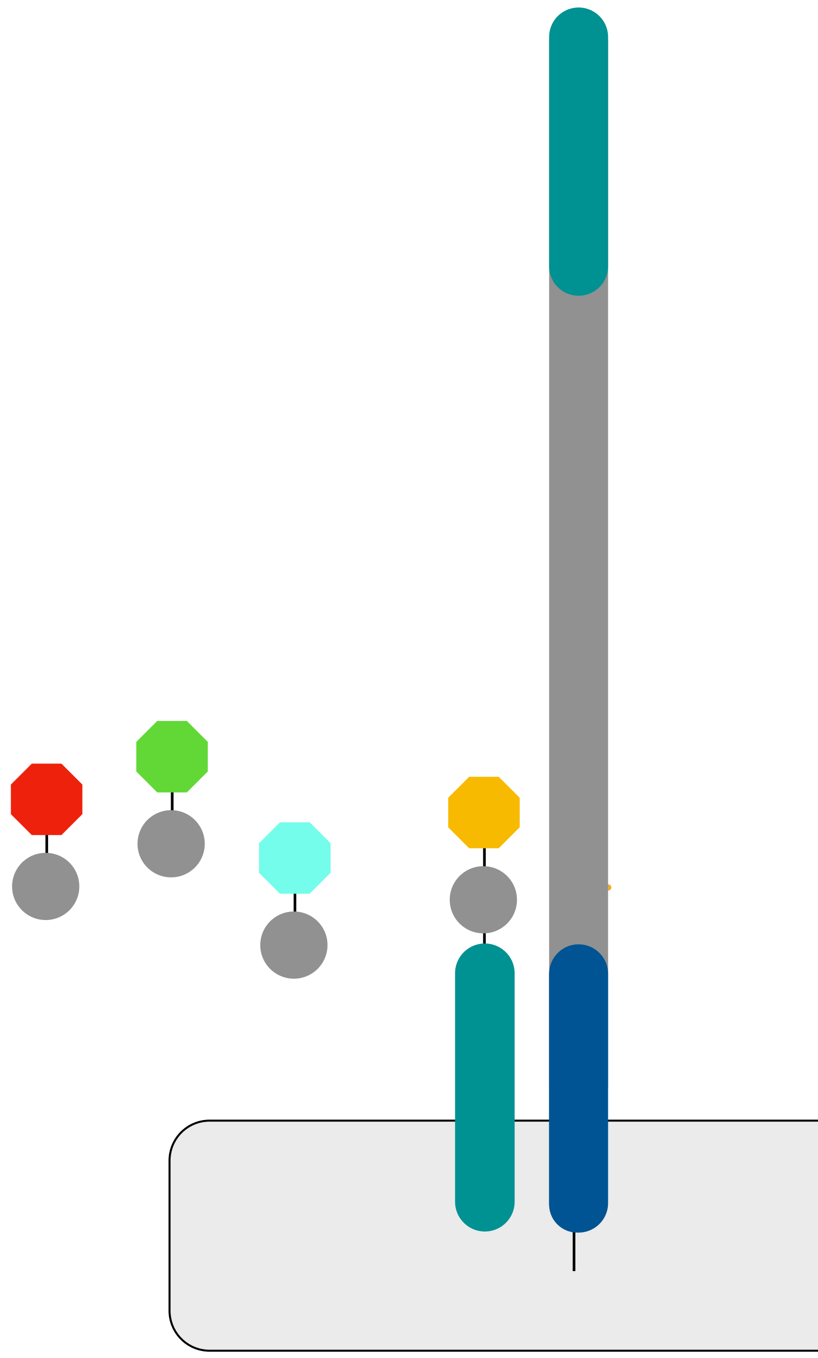

Illumina sequencing

sequencing by synthesis

polymerase
reversible terminator fluorescent dNTPS

if complementary, polymerase adds it

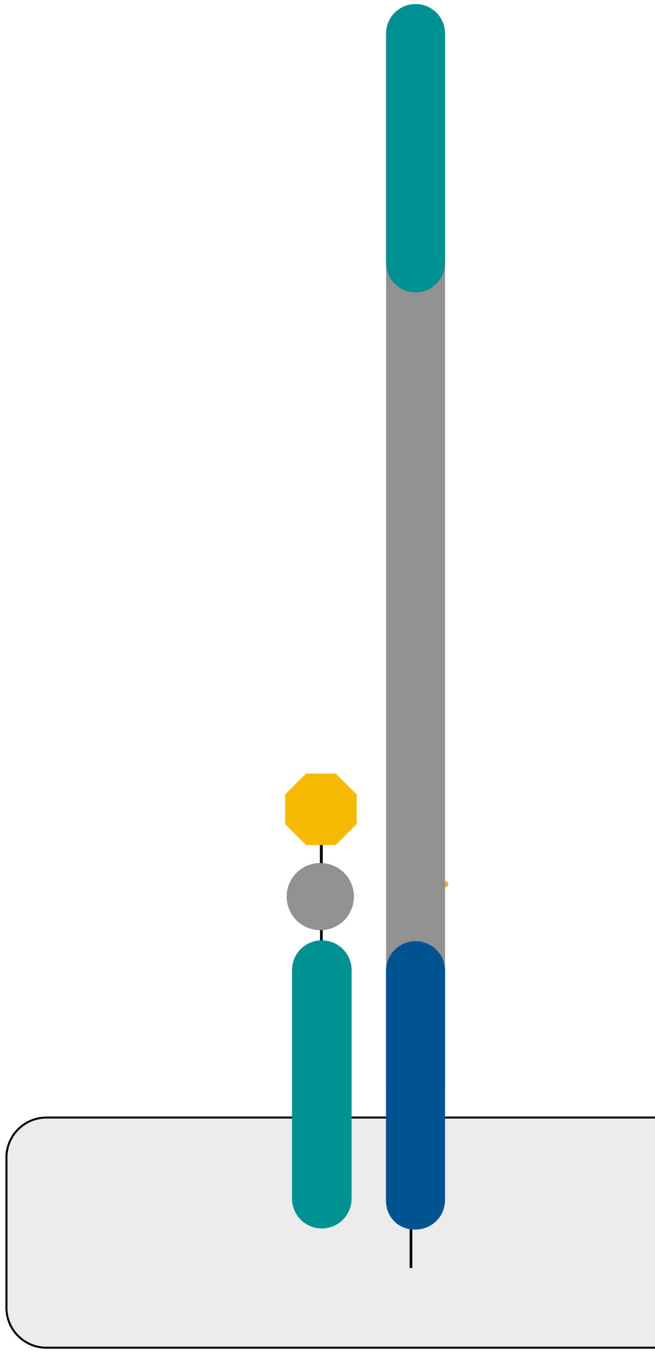

Illumina sequencing

sequencing by synthesis

polymerase
reversible terminator fluorescent dNTPS

if complementary, polymerase adds it

take picture

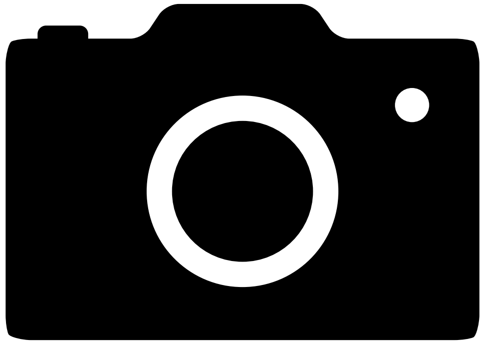

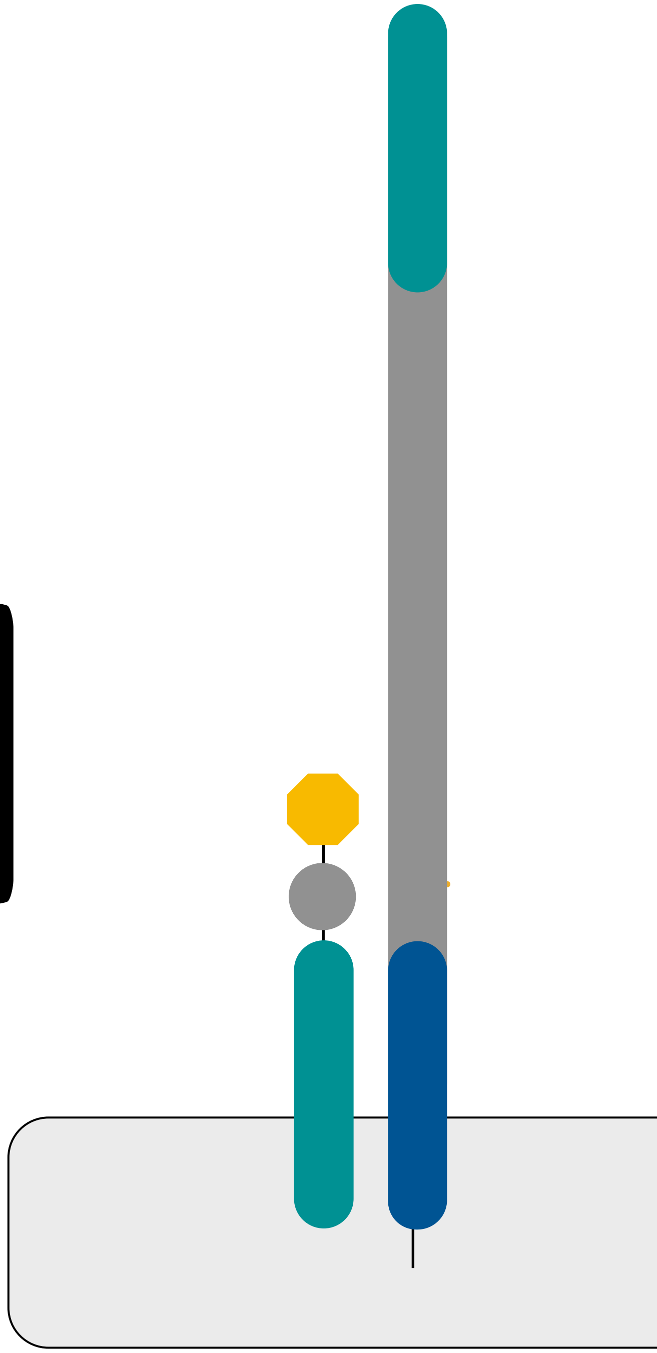

Illumina sequencing

sequencing by synthesis

polymerase
reversible terminator fluorescent dNTPS

if complementary, polymerase adds it

take picture

remove terminator and fluorophore

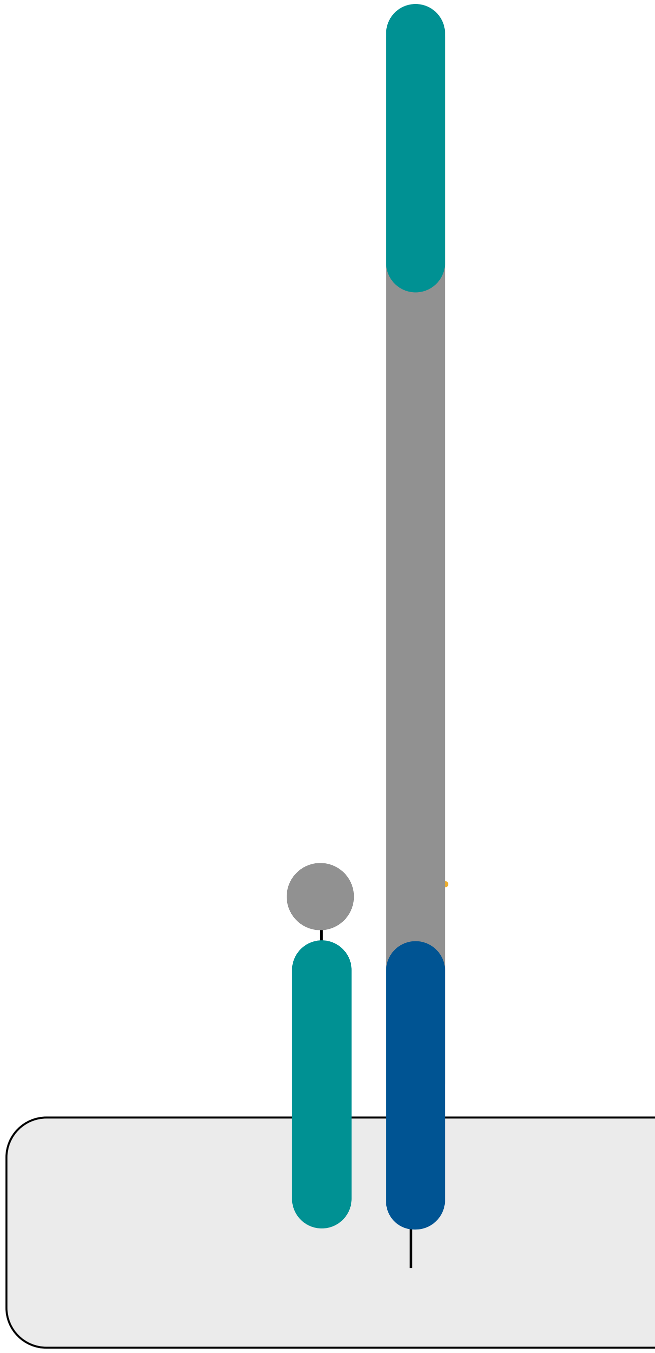

Illumina sequencing

sequencing by synthesis

polymerase
reversible terminator fluorescent dNTPS

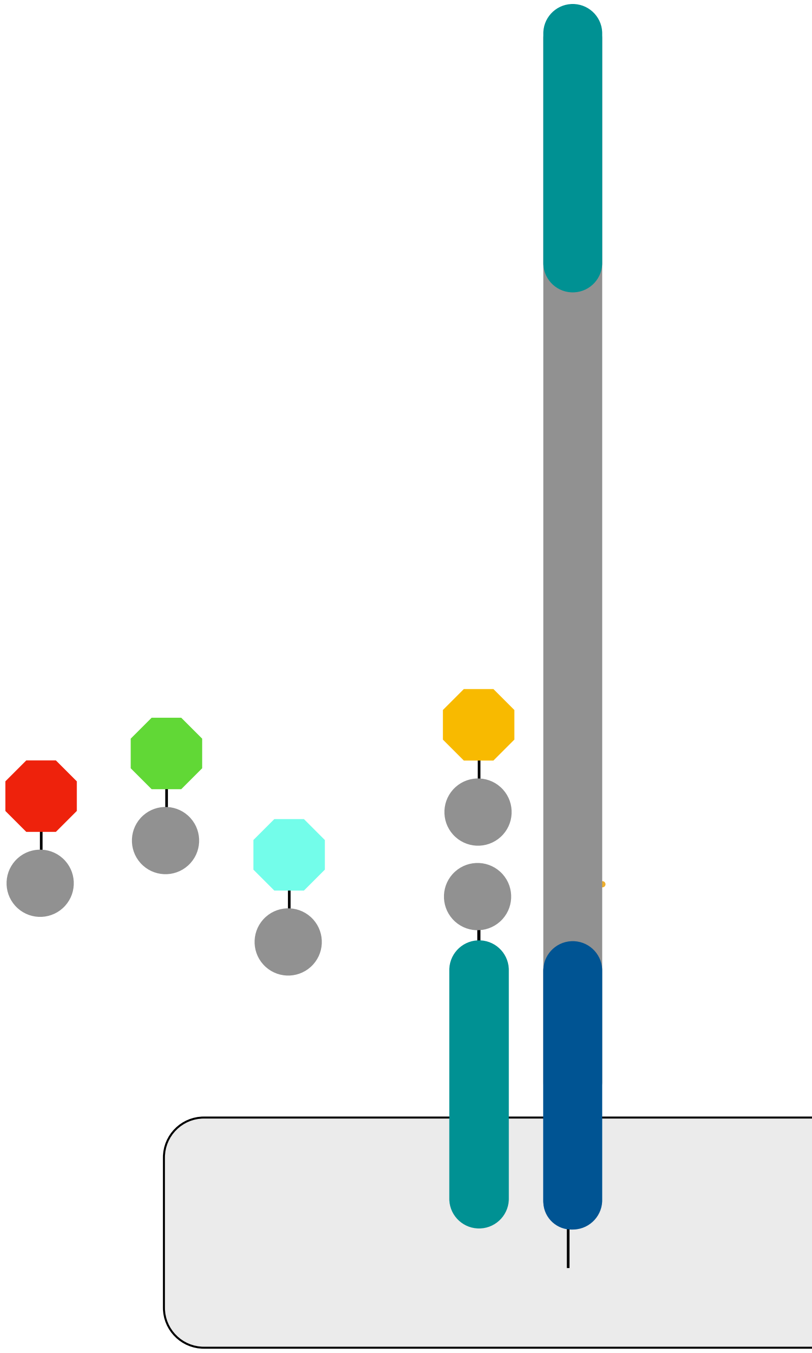

Illumina sequencing

sequencing by synthesis

polymerase
reversible terminator fluorescent dNTPS

if complementary, polymerase adds it

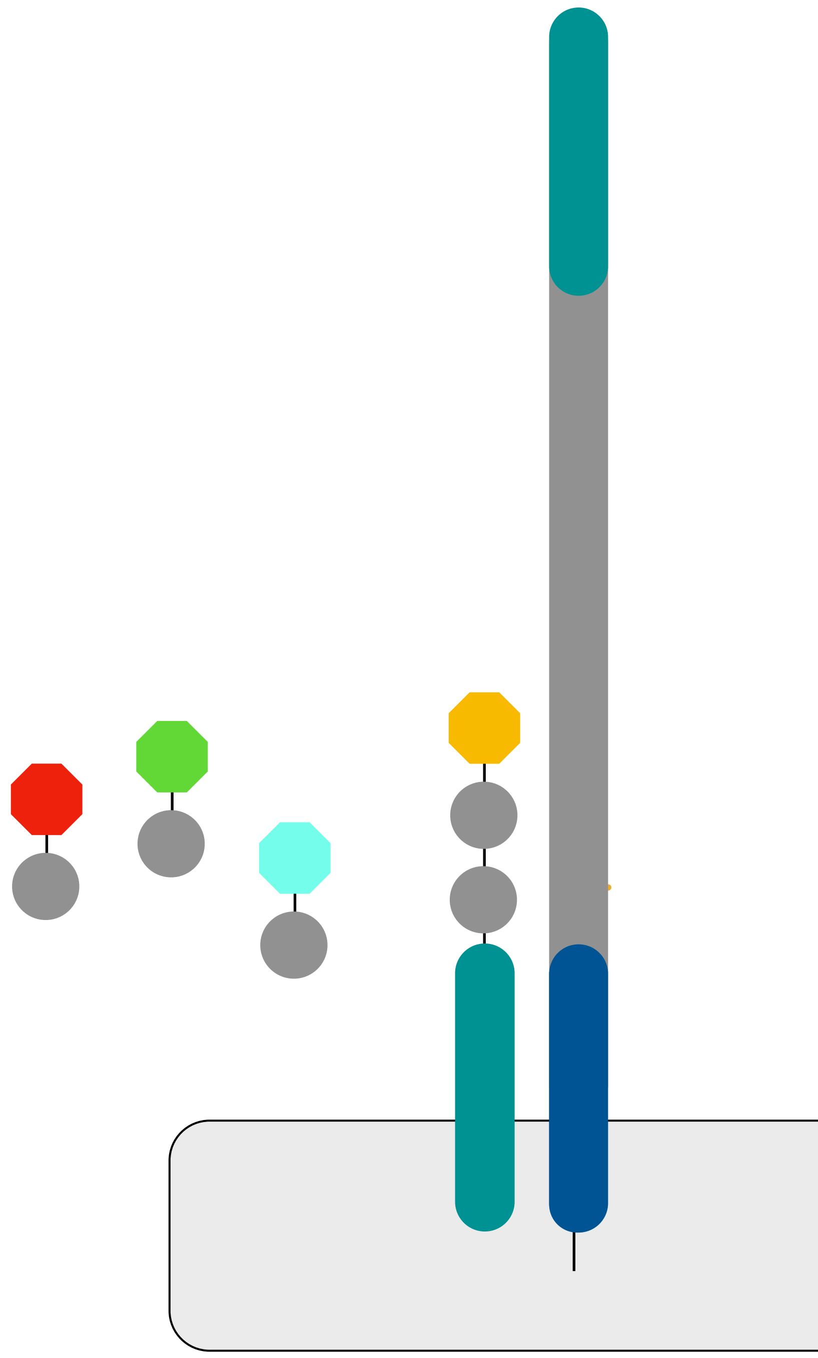

Illumina sequencing

sequencing by synthesis

polymerase
reversible terminator fluorescent dNTPS

if complementary, polymerase adds it

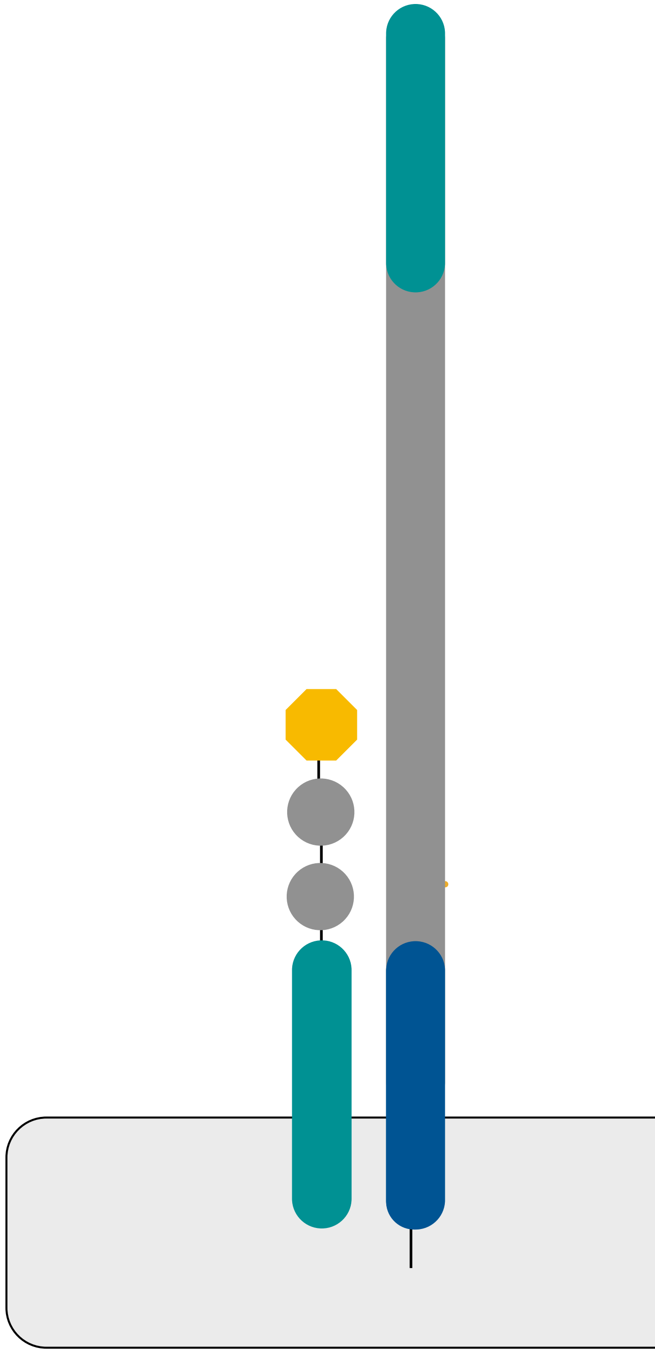

Illumina sequencing

sequencing by synthesis

polymerase
reversible terminator fluorescent dNTPS

if complementary, polymerase adds it

take picture

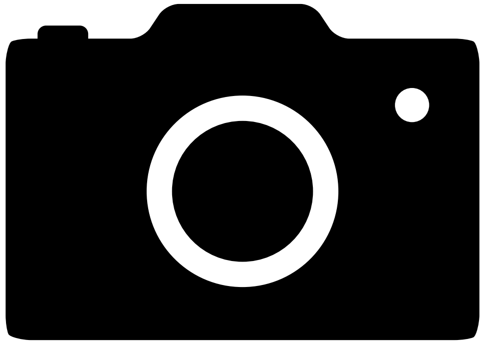

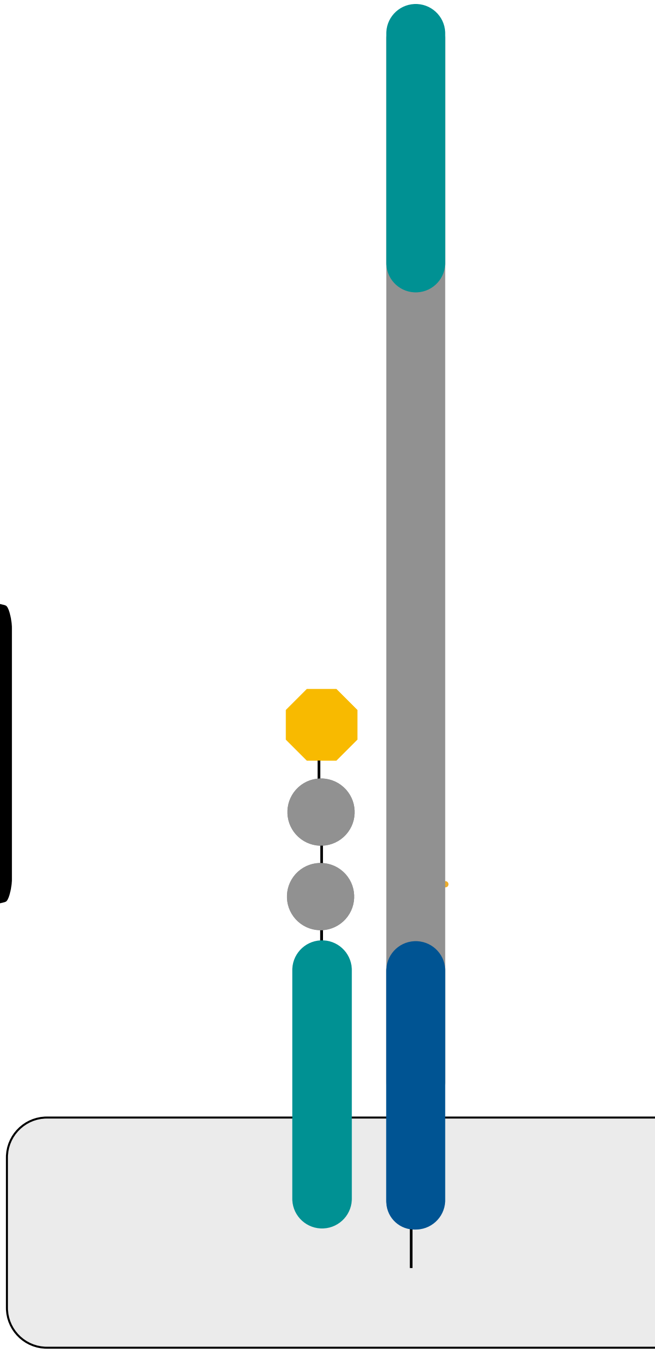

Illumina sequencing

sequencing by synthesis

polymerase
reversible terminator fluorescent dNTPS

if complementary, polymerase adds it

take picture

remove terminator and fluorophore

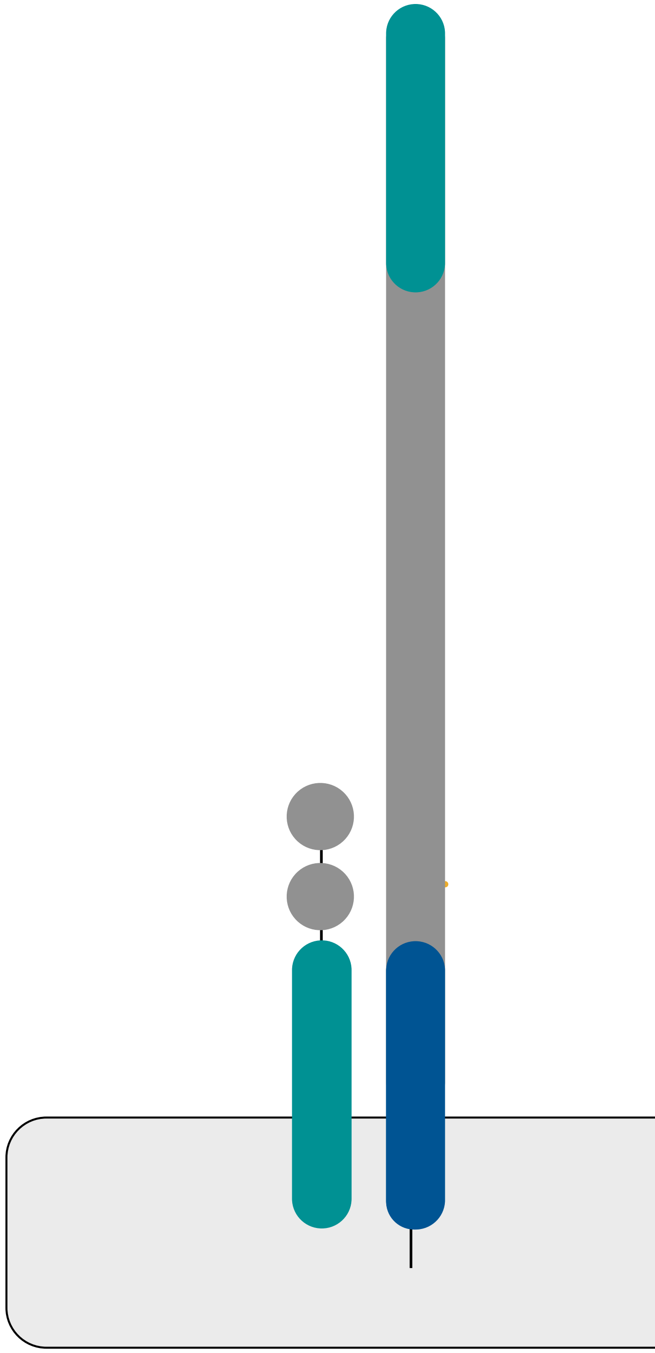

Illumina sequencing

sequencing by synthesis

polymerase
reversible terminator fluorescent dNTPS

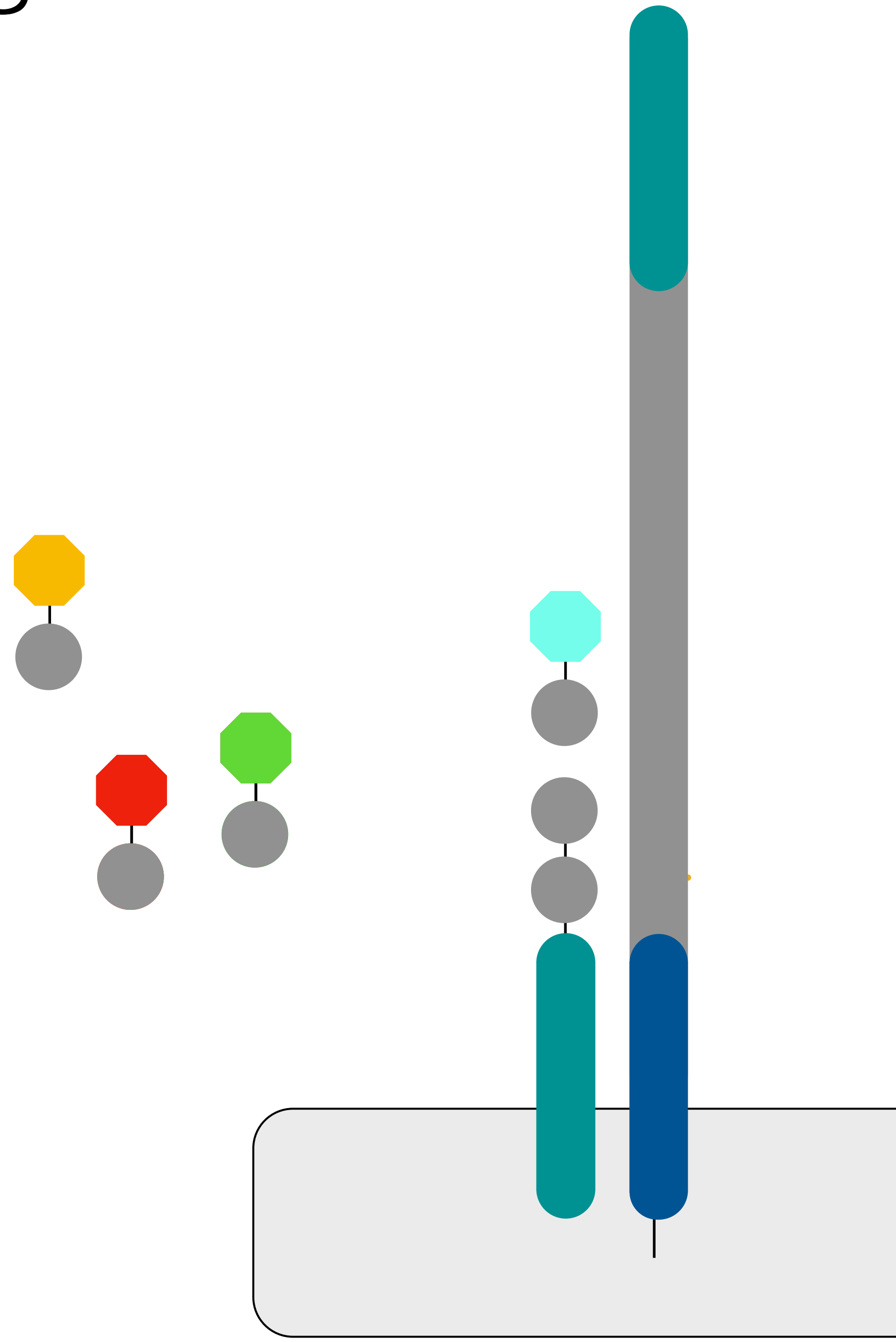

Illumina sequencing

sequencing by synthesis

polymerase
reversible terminator fluorescent dNTPS

if complementary, polymerase adds it

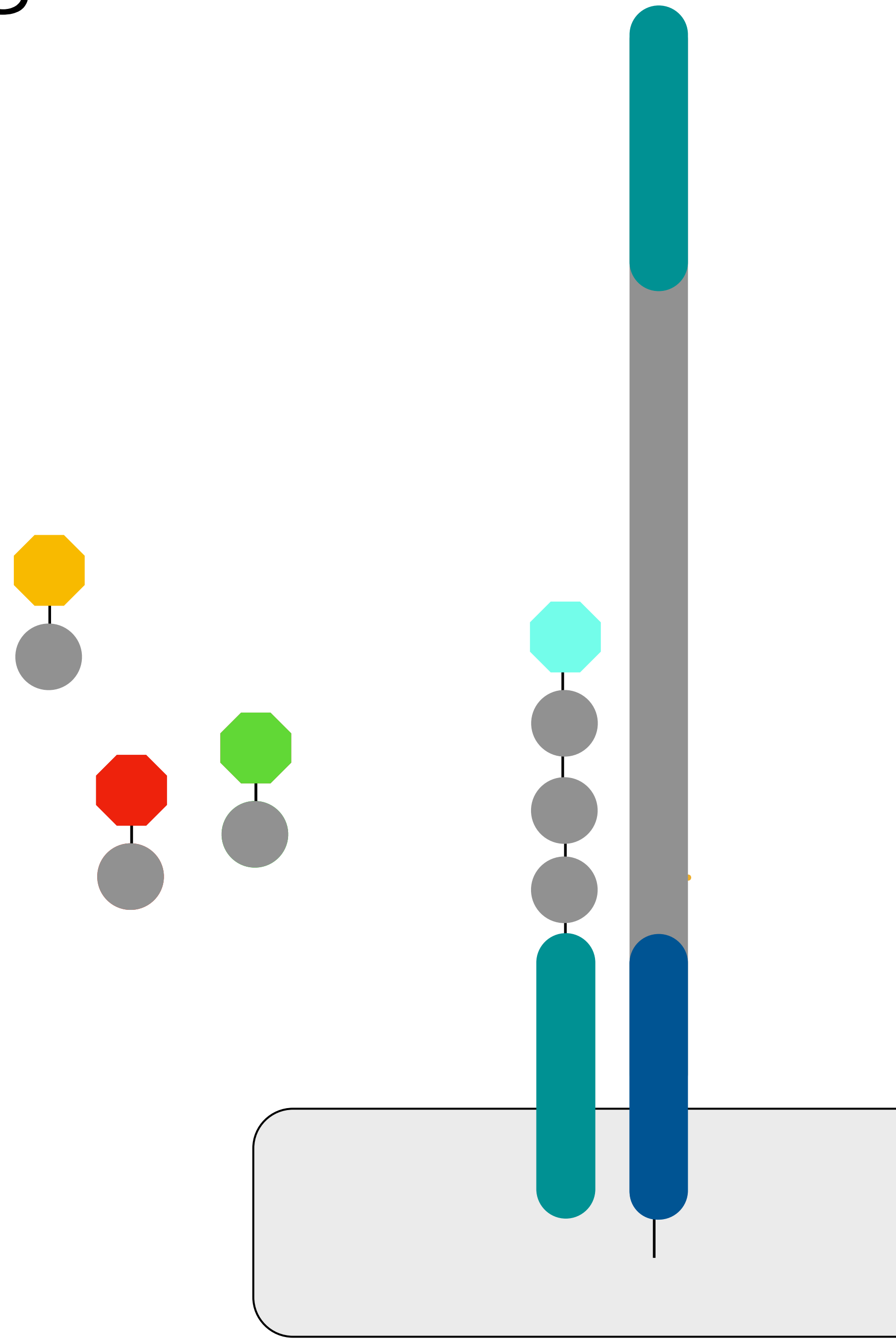

Illumina sequencing

sequencing by synthesis

polymerase
reversible terminator fluorescent dNTPS

if complementary, polymerase adds it

take picture

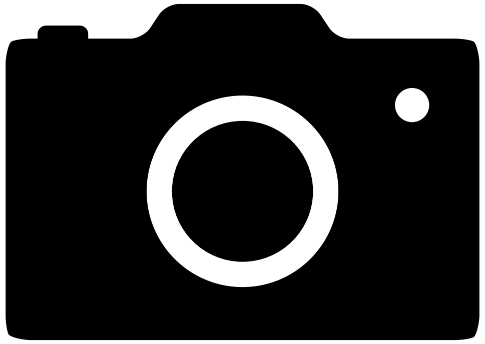

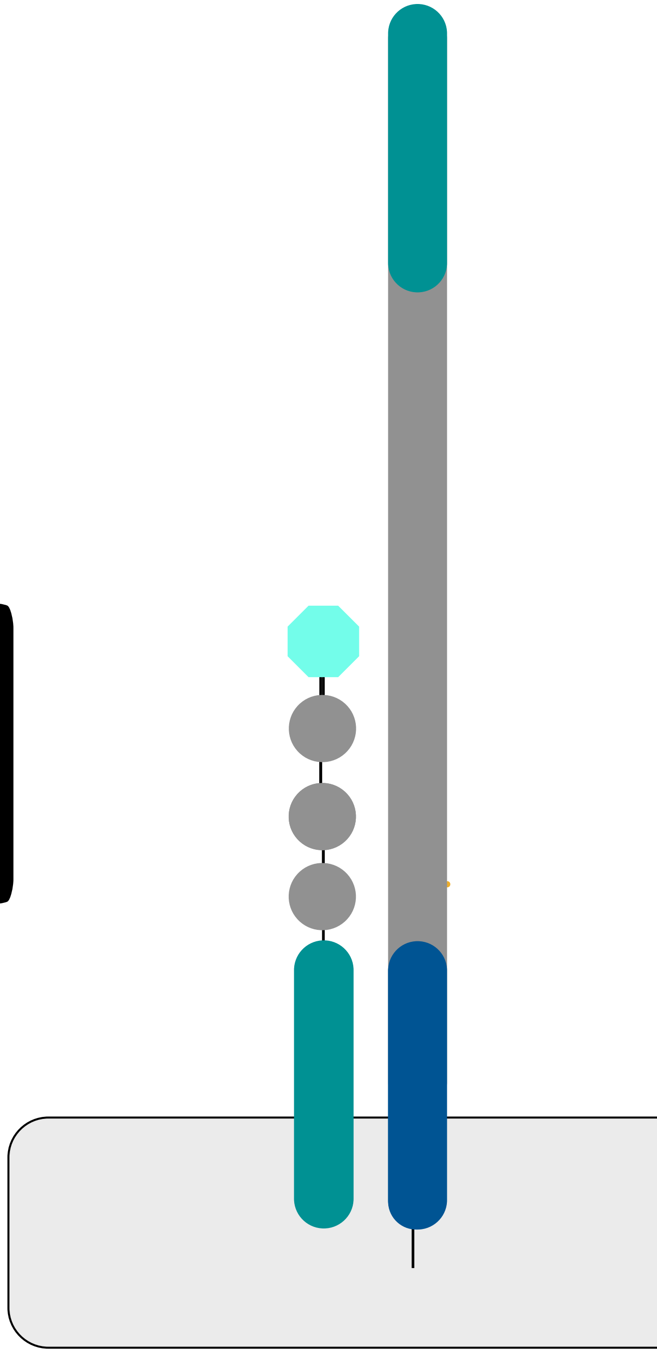

Microbial DNA library prep

By the end of today you will:

- be able to explain the key steps of Illumina sequencing

- be able to read Illumina sequencing output

Microbial DNA library prep

By the end of today you will:

- be able to explain the key steps of Illumina sequencing

- be able to read Illumina sequencing output

Active Learning Question

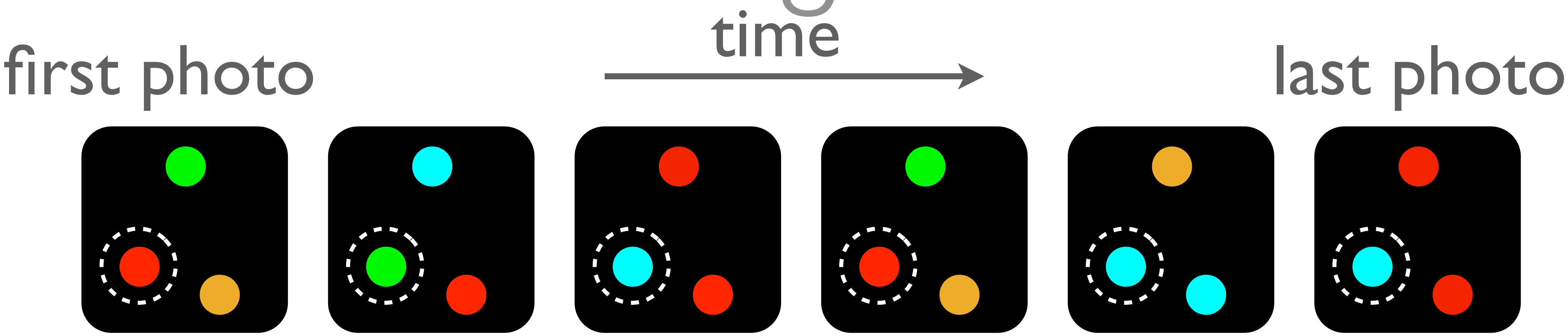

Using the code in the box, and the sequencing photos above (with the circled cluster), what is the sequence of the synthesized DNA?

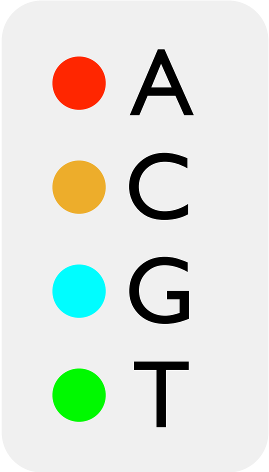

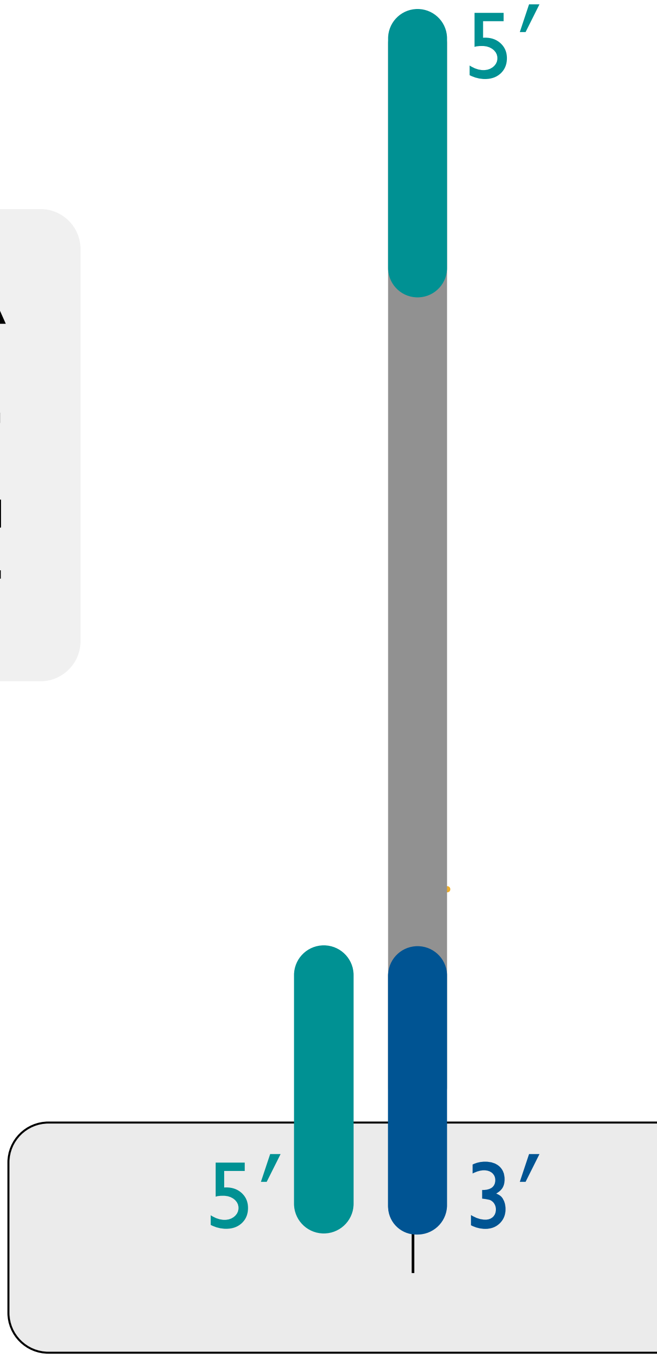

Active Learning Question

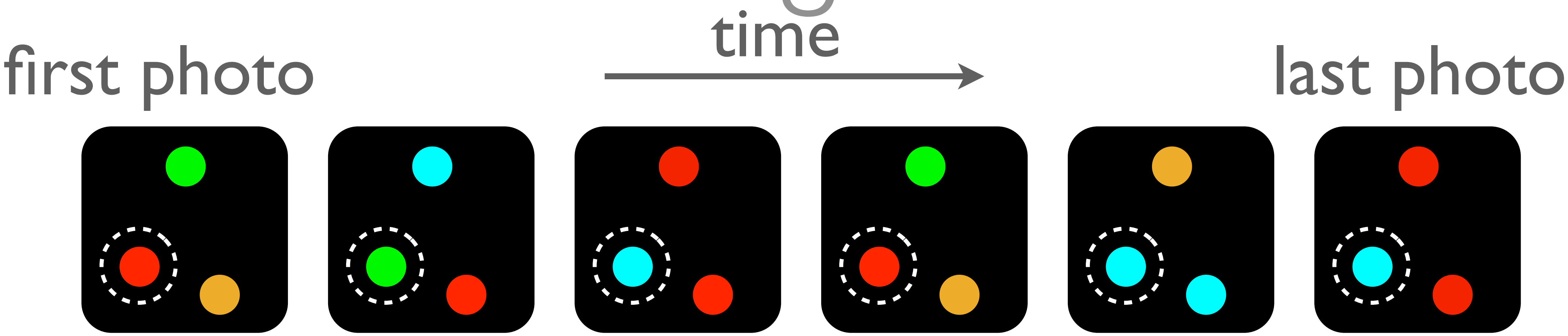

Using the code in the box, and the sequencing photos above (with the circled cluster), what is the sequence of the synthesized DNA?

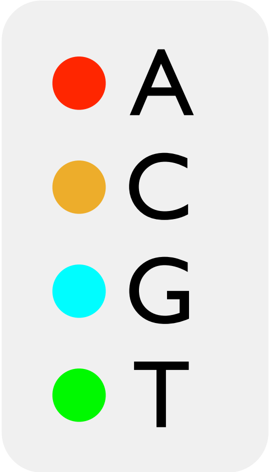

A) 5′GGAGTA

B) 5′ATGAGG

C) 5′CCTCAT

D) 5′TACTCC

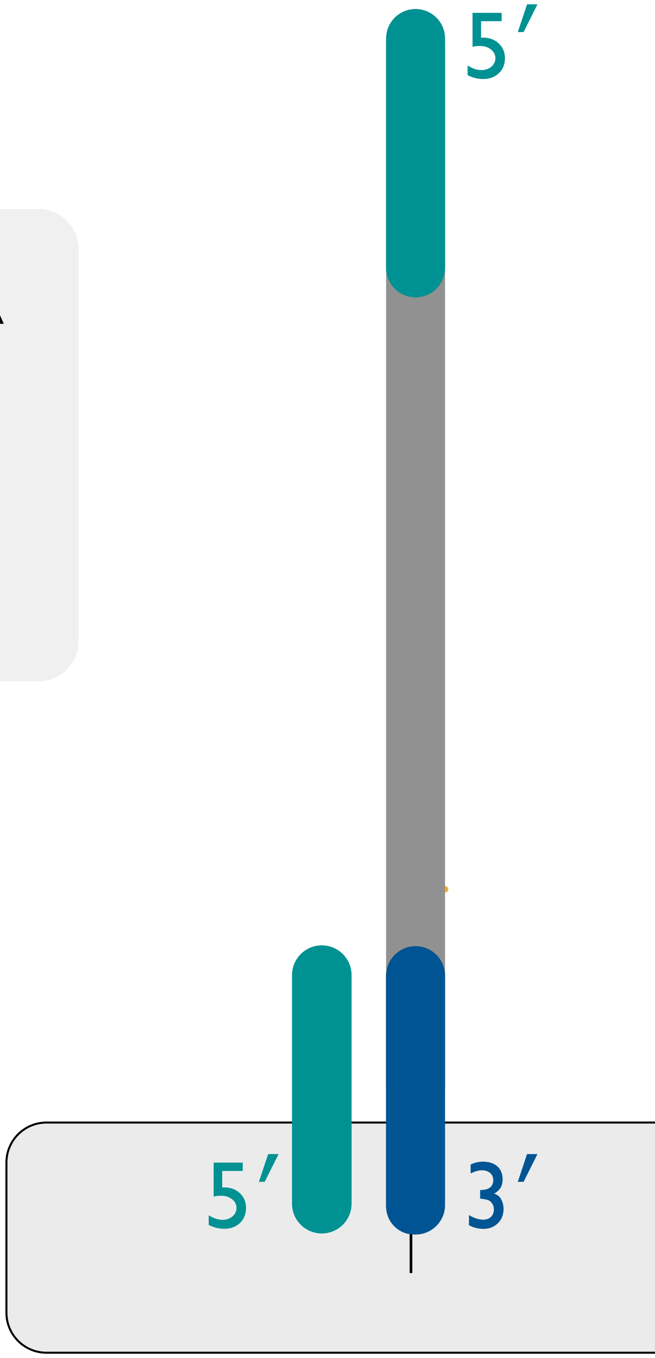

Active Learning Question

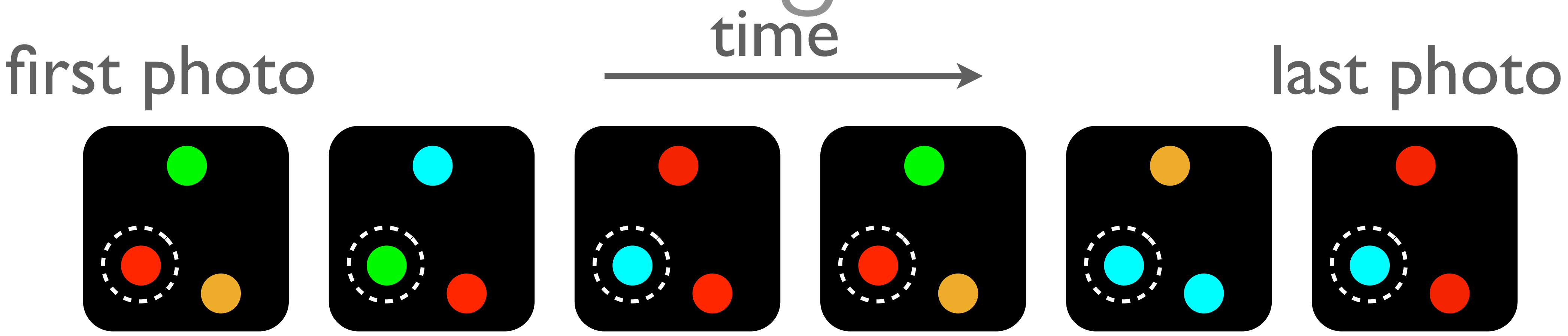

Using the code in the box, and the sequencing photos above (with the circled cluster), what is the sequence of the synthesized DNA?

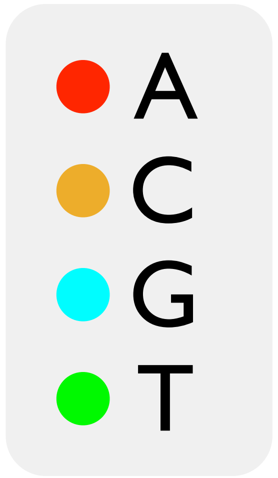

A) 5′GGAGTA

B) 5′ATGAGG

C) 5′CCTCAT

D) 5′TACTCC

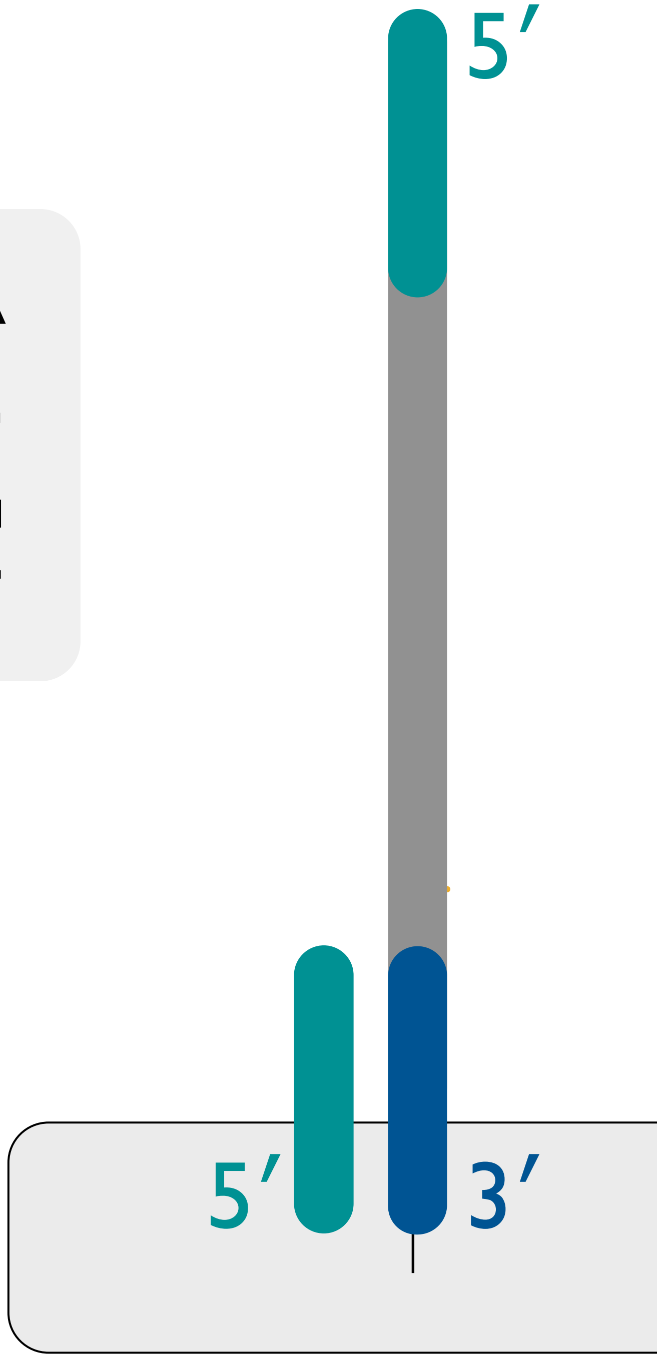

First 2 steps = “library prep”

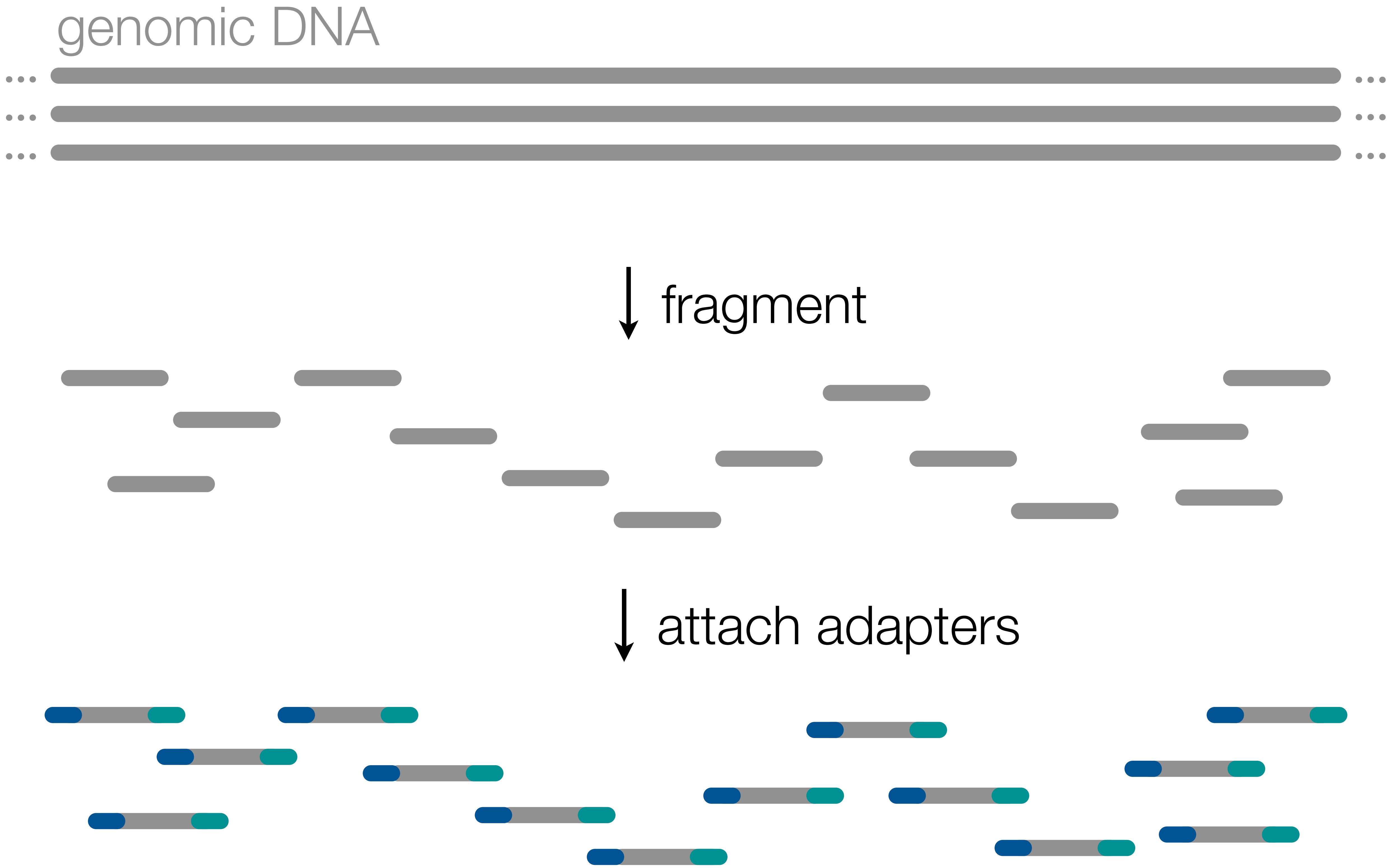

adapters = short pieces of DNA (“oligonucleotides”)
