# Supplementary material for: Resequencing of Microbial Isolates: A Lab Module to Introduce Novices to Command-Line Bioinformatics
Source: Front Microbiol. 2021 Mar 16;12:578859. doi: 10.3389/fmicb.2021.578859 (PMC8008064; doi:10.3389/fmicb.2021.578859)
Supplement: Supplementary file 3 [file Data_Sheet_3.PDF]

Microbial DNA library prep

By the end of today you will:

- be able to calculate the volume of DNA solution you need to add to get a specified mass of DNA

- be able to explain the purpose of each step of DNA library prep for Illumina sequencing

Katherine Petrie

Microbial DNA library prep

By the end of today you will:

- be able to calculate the volume of DNA solution you need to add to get a specified mass of DNA

- be able to explain the purpose of each step of DNA library prep for Illumina sequencing

How much DNA do you have?

mass, volume, and mass per unit volume

How much DNA will you put into library prep?

3. **Determine how much of your DNA to use.** You can add **between 2 µL and 30 µL of volume**, but will have to use the DNA concentration to calculate the best volume to add. The **ideal amount of DNA is 500 ng**, but less is OK. Reminder: (ng/µL) * (µL) = ng. **Show your calculation to your IA before moving to the next step.**

Active Learning Question

You think you have enough DNA to add all 500 ng. According to the qubit, your DNA concentration is 250 ng/uL. What volume should you add in the library prep?

A) 0.5 uL

B) 2 uL

C) 20 uL

D) 50 uL

E) 500 ng

Active Learning Question

You think you have enough DNA to add all 500 ng. According to the qubit, your DNA concentration is 250 ng/uL. What volume should you add in the library prep?

A) 0.5 uL

B) 2 uL

C) 20 uL

D) 50 uL

E) 500 ng

Active Learning Question

You think you have enough DNA to add all 500 ng. According to the qubit, your DNA concentration is 250 ng/uL. What volume should you add in the library prep?

A) 0.5 uL

B) 2 uL

C) 20 uL

D) 50 uL

E) 500 ng

$$\frac{\text{mass}}{\text{volume}} \times \text{volume} = \text{mass}$$

Active Learning Question

You think you have enough DNA to add all 500 ng. According to the qubit, your DNA concentration is 250 ng/uL. What volume should you add in the library prep?

A) 0.5 uL

B) 2 uL

C) 20 uL

D) 50 uL

E) 500 ng

$$\frac{\text{mass}}{\text{volume}} \times \text{volume} = \text{mass}$$

$$\frac{\text{ng}}{\text{uL}} \times \text{uL} = \text{ng}$$

Active Learning Question

You think you have enough DNA to add all 500 ng. According to the qubit, your DNA concentration is 250 ng/uL. What volume should you add in the library prep?

A) 0.5 uL

B) 2 uL

C) 20 uL

D) 50 uL

E) 500 ng

$$\frac{\text{mass}}{\text{volume}} \times \text{volume} = \text{mass}$$

$$\frac{250\ \text{ng}}{\text{uL}} \times ?\ \text{uL} = 500\ \text{ng}$$

Active Learning Question

Your groupmates' DNA is not as concentrated as yours. If their DNA is 10 ng/uL, and they add the 30 uL maximum to the 20 uL of diluted BLT during the first step of library prep, what is the **amount** of DNA they put in to the library prep?

A) 10 ng

B) 30 ng

C) 30 ng/uL

D) 300 ng

E) 6 ng/uL

Active Learning Question

Your groupmates' DNA is not as concentrated as yours. If their DNA is 10 ng/uL, and they add the 30 uL maximum to the 20 uL of diluted BLT during the first step of library prep, what is the **amount** of DNA they put in to the library prep?

A) 10 ng

B) 30 ng

C) 30 ng/uL

D) 300 ng

E) 6 ng/uL

Active Learning Question

Your groupmates' DNA is not as concentrated as yours. If their DNA is 10 ng/uL, and they add the 30 uL maximum to the 20 uL of diluted BLT during the first step of library prep, what is the **amount** of DNA they put in to the library prep?

A) 10 ng

B) 30 ng

C) 30 ng/uL

D) 300 ng

E) 6 ng/uL

$$30 \text{ uL} \times \frac{10 \text{ ng}}{\text{uL}}$$

Active Learning Question

Your groupmates' DNA is not as concentrated as yours. If their DNA is 10 ng/uL, and they add the 30 uL maximum to the 20 uL of diluted BLT during the first step of library prep, what is the **amount** of DNA they put in to the library prep?

A) 10 ng

B) 30 ng

C) 30 ng/uL

D) 300 ng

E) 6 ng/uL

$$30\ \cancel{\text{uL}} \times \frac{10\ \text{ng}}{\cancel{\text{uL}}} =$$

Active Learning Question

Your groupmates' DNA is not as concentrated as yours. If their DNA is 10 ng/uL, and they add the 30 uL maximum to the 20 uL of diluted BLT during the first step of library prep, what is the **amount** of DNA they put in to the library prep?

A) 10 ng

B) 30 ng

C) 30 ng/uL

D) 300 ng

E) 6 ng/uL

$$30\ \cancel{uL} \times \frac{10\ ng}{\cancel{uL}} = 300\ ng$$

Active Learning Question

If your prepared library measured 2 ng/uL, and you have 25 uL of it left, what is the **amount** of library DNA you have?

A) 60 ng

B) 50 ng

C) 2 ng/uL

D) 50 ng/L

E) 12.5 ng

Active Learning Question

If your prepared library measured 2 ng/uL, and you have 25 uL of it left, what is the **amount** of library DNA you have?

A) 60 ng

B) 50 ng

C) 2 ng/uL

D) 50 ng/L

E) 12.5 ng

Active Learning Question

If your prepared library measured 2 ng/uL, and you have 25 uL of it left, what is the **amount** of library DNA you have?

A) 60 ng

B) 50 ng

C) 2 ng/uL

D) 50 ng/L

E) 12.5 ng

$$\frac{2\ \text{ng}}{\text{uL}} \times 25\ \text{uL} =$$

Active Learning Question

If your prepared library measured 2 ng/uL, and you have 25 uL of it left, what is the **amount** of library DNA you have?

A) 60 ng

B) 50 ng

C) 2 ng/uL

D) 50 ng/L

E) 12.5 ng

$$\frac{2\ \text{ng}}{\cancel{\text{uL}}} \times 25\ \cancel{\text{uL}} =$$

Active Learning Question

If your prepared library measured 2 ng/uL, and you have 25 uL of it left, what is the **amount** of library DNA you have?

A) 60 ng

B) 50 ng

C) 2 ng/uL

D) 50 ng/L

E) 12.5 ng

$$\frac{2\ \text{ng}}{\cancel{\text{uL}}} \times 25\ \cancel{\text{uL}} = 50\ \text{ng}$$

Microbial DNA library prep

By the end of today you will:

- be able to calculate the volume of DNA solution you need to add to get a specified mass of DNA

- be able to explain the purpose of each step of DNA library prep for Illumina sequencing

Microbial DNA library prep

By the end of today you will:

- be able to calculate the volume of DNA solution you need to add to get a specified mass of DNA

- **be able to explain the purpose of each step of DNA library prep for Illumina sequencing**

Preparing a sequencing 'library'

fragmenting genomes and attaching adaptors

BLTs make purification easier

"bead linked transposomes"

-transposase enzymes attached to beads snip out fragments of the genome, adding adaptors and affixing them to the beads in the process

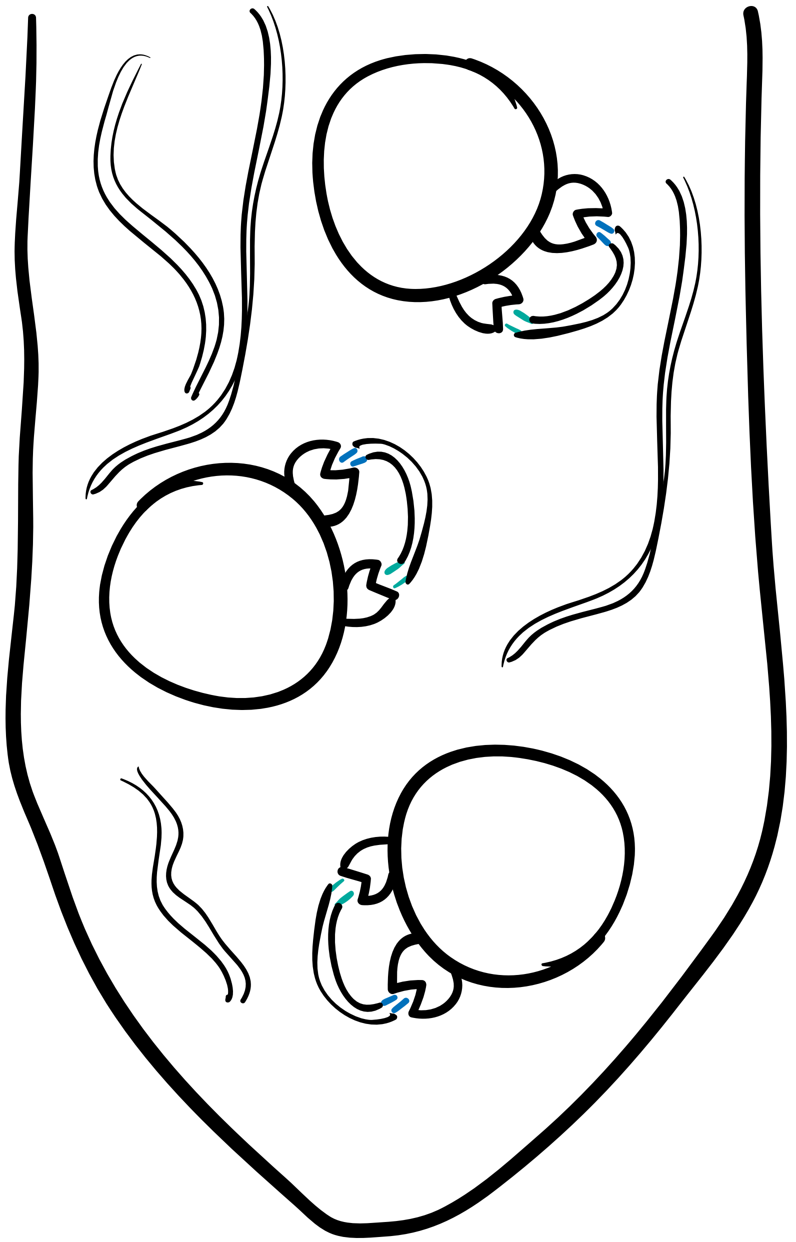

Preparing a sequencing 'library'

fragmenting genomes and attaching adaptors

BLTs make purification easier

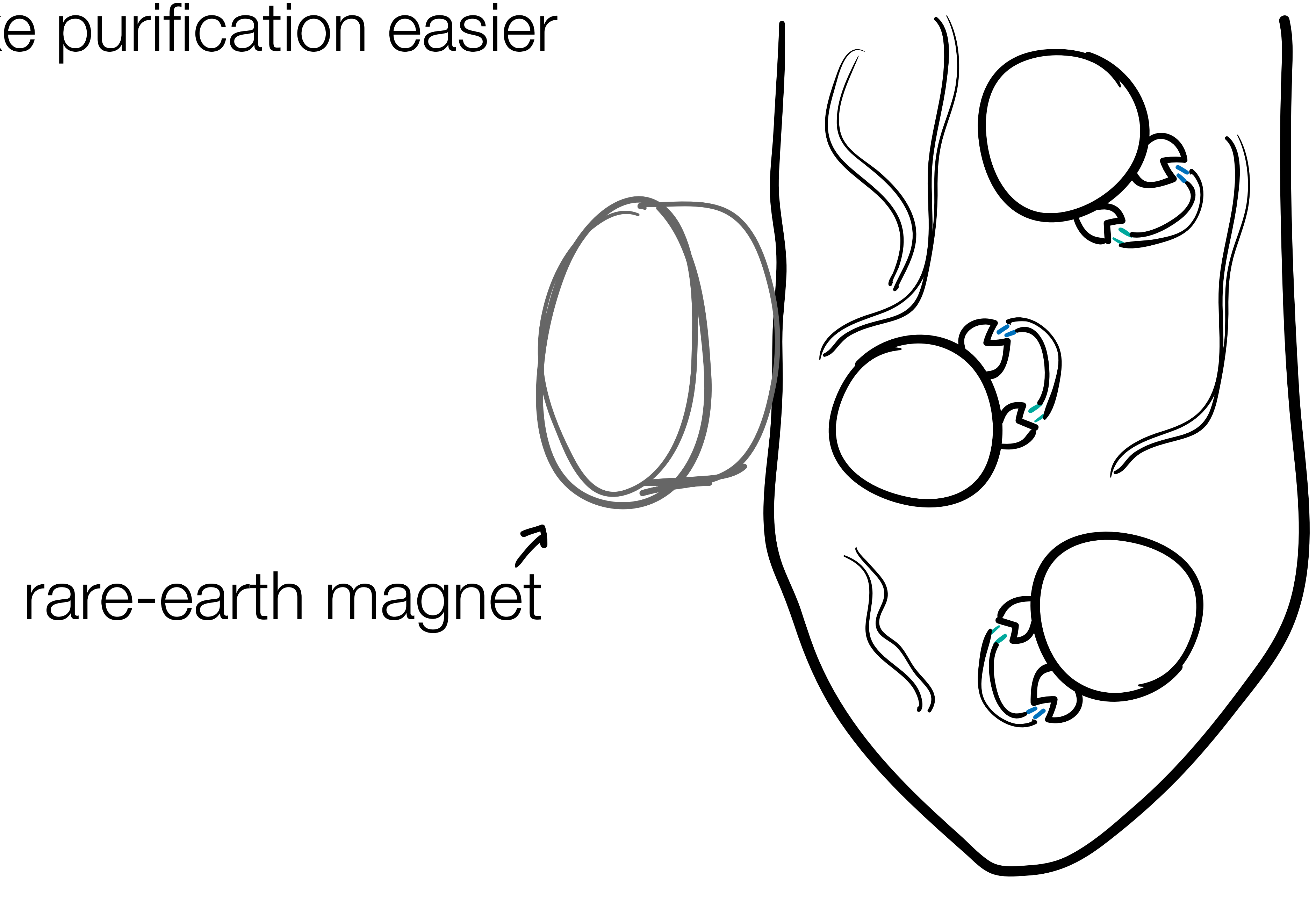

Preparing a sequencing 'library'

fragmenting genomes and attaching adaptors

BLTs make purification easier

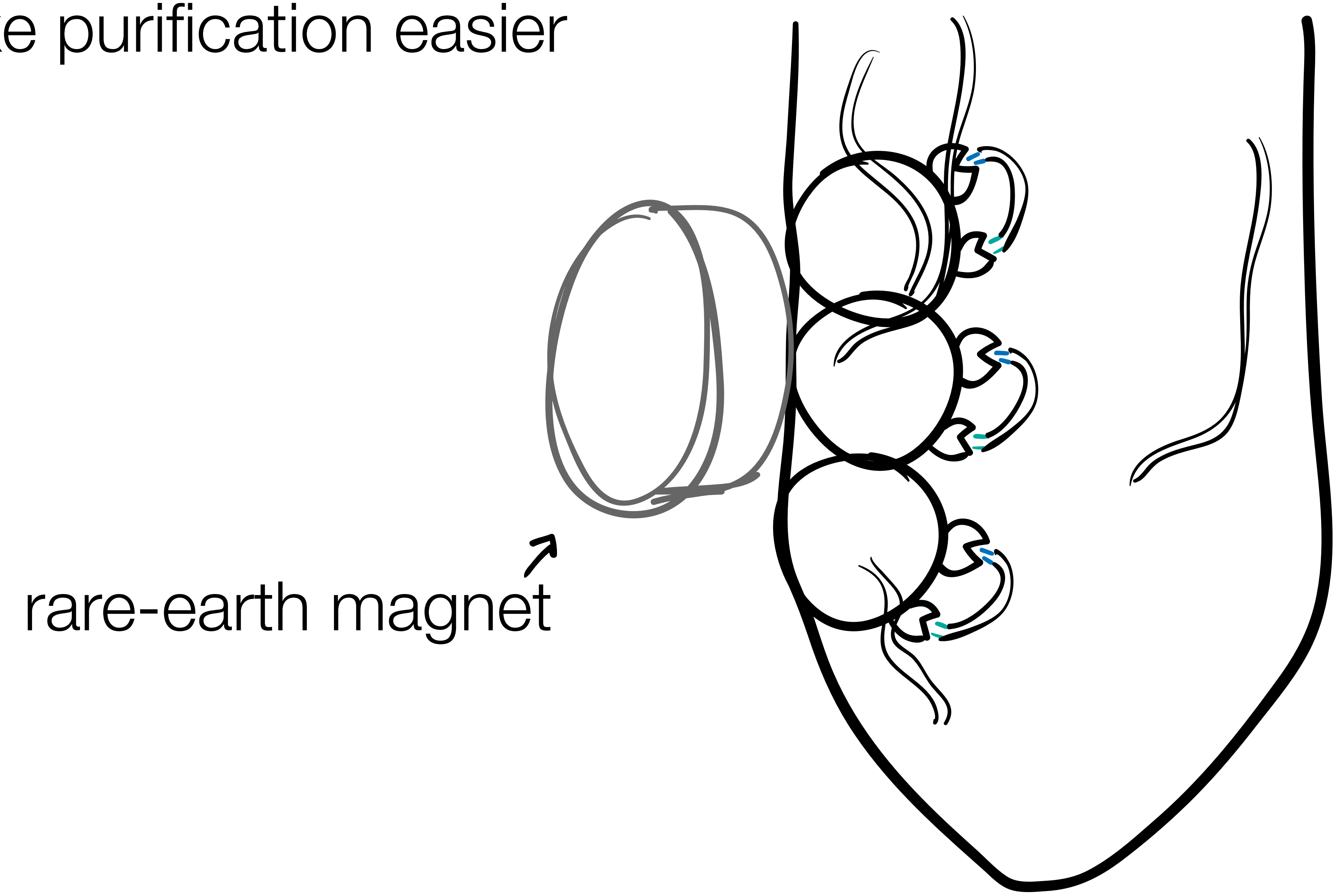

Preparing a sequencing 'library'

fragmenting genomes and attaching adaptors

BLTs make purification easier

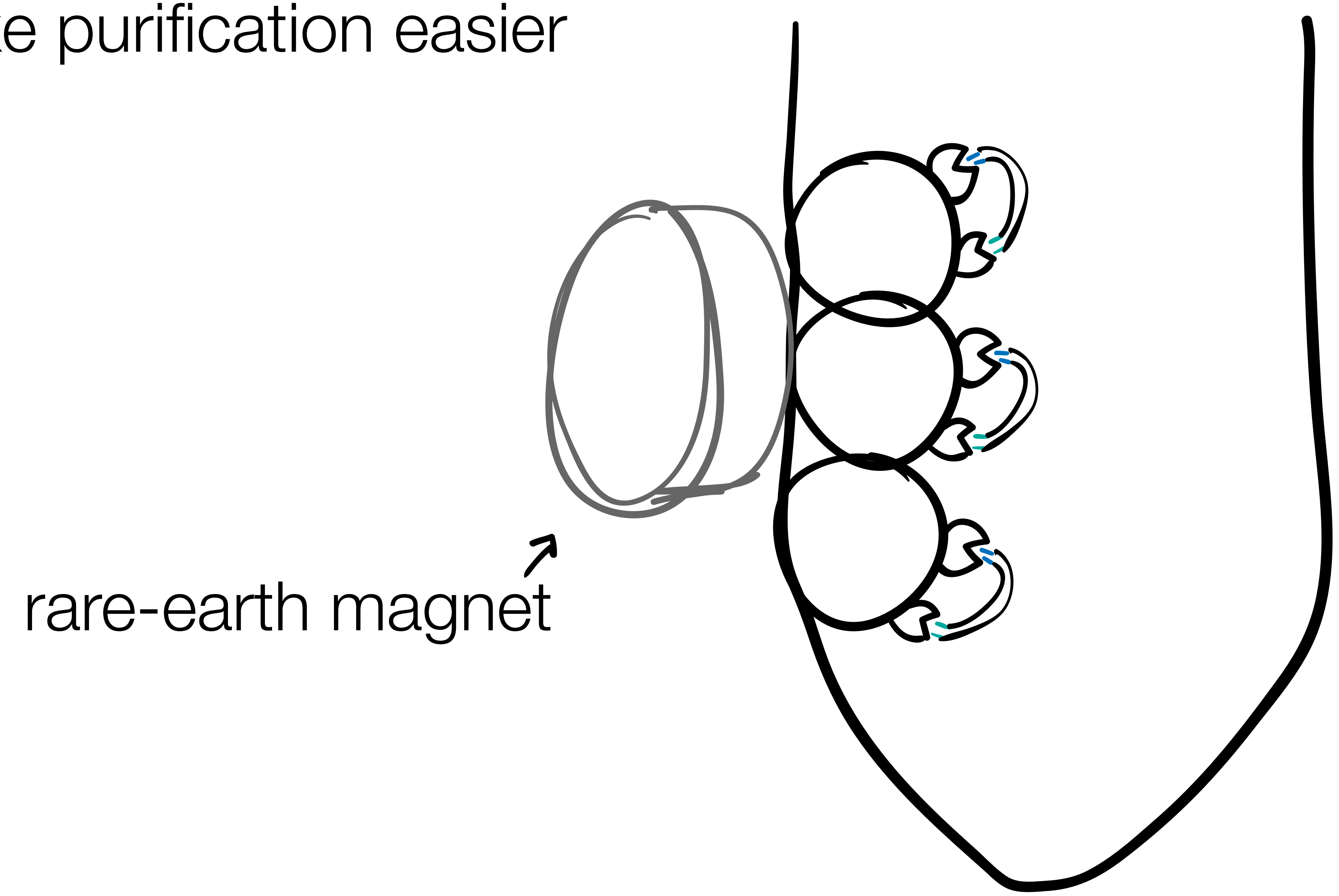

Preparing a sequencing ‘library’

fragmenting genomes and attaching adaptors

BLTs make purification easier

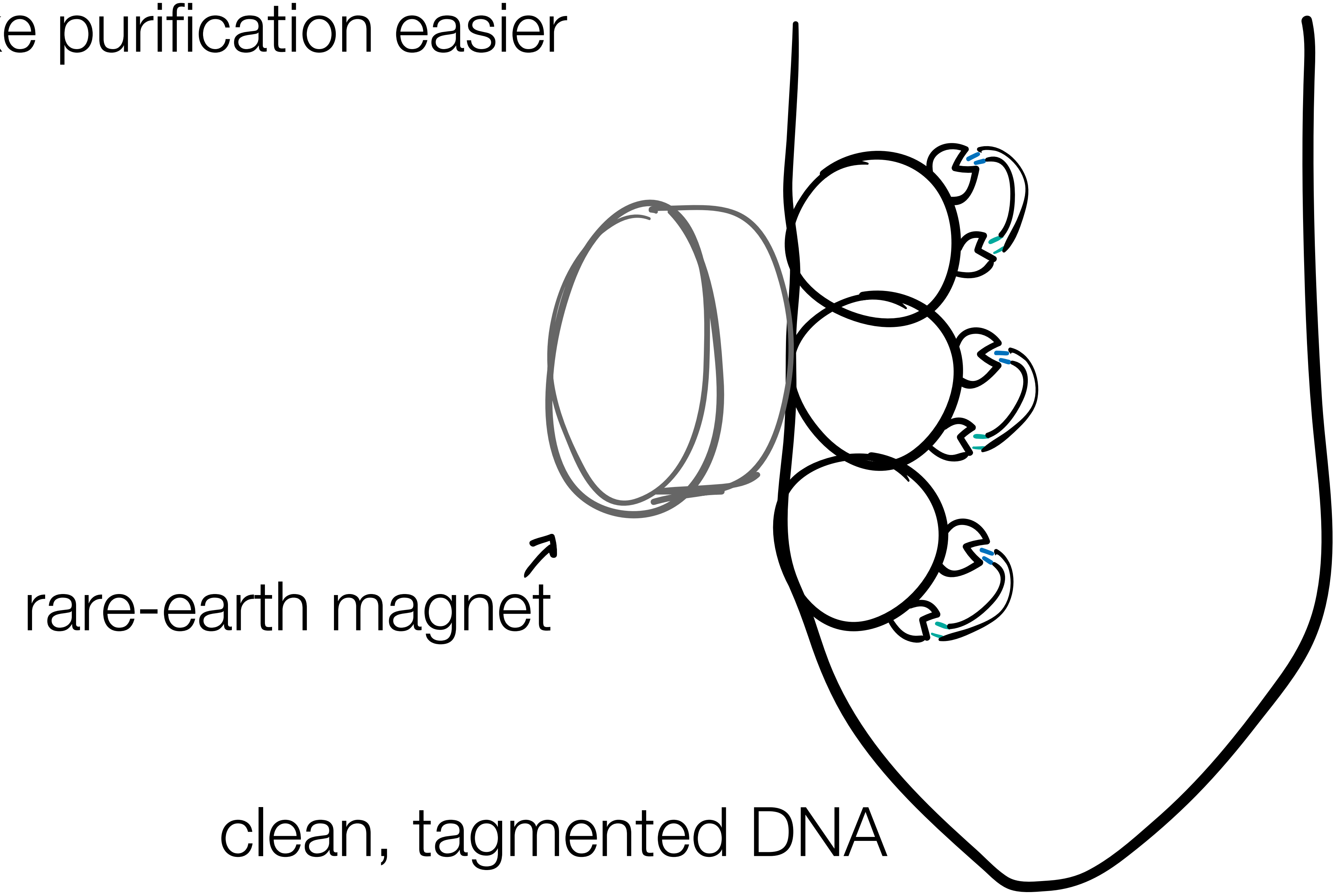

Preparing a sequencing 'library'

fragmenting genomes and attaching adaptors

BLTs make purification easier

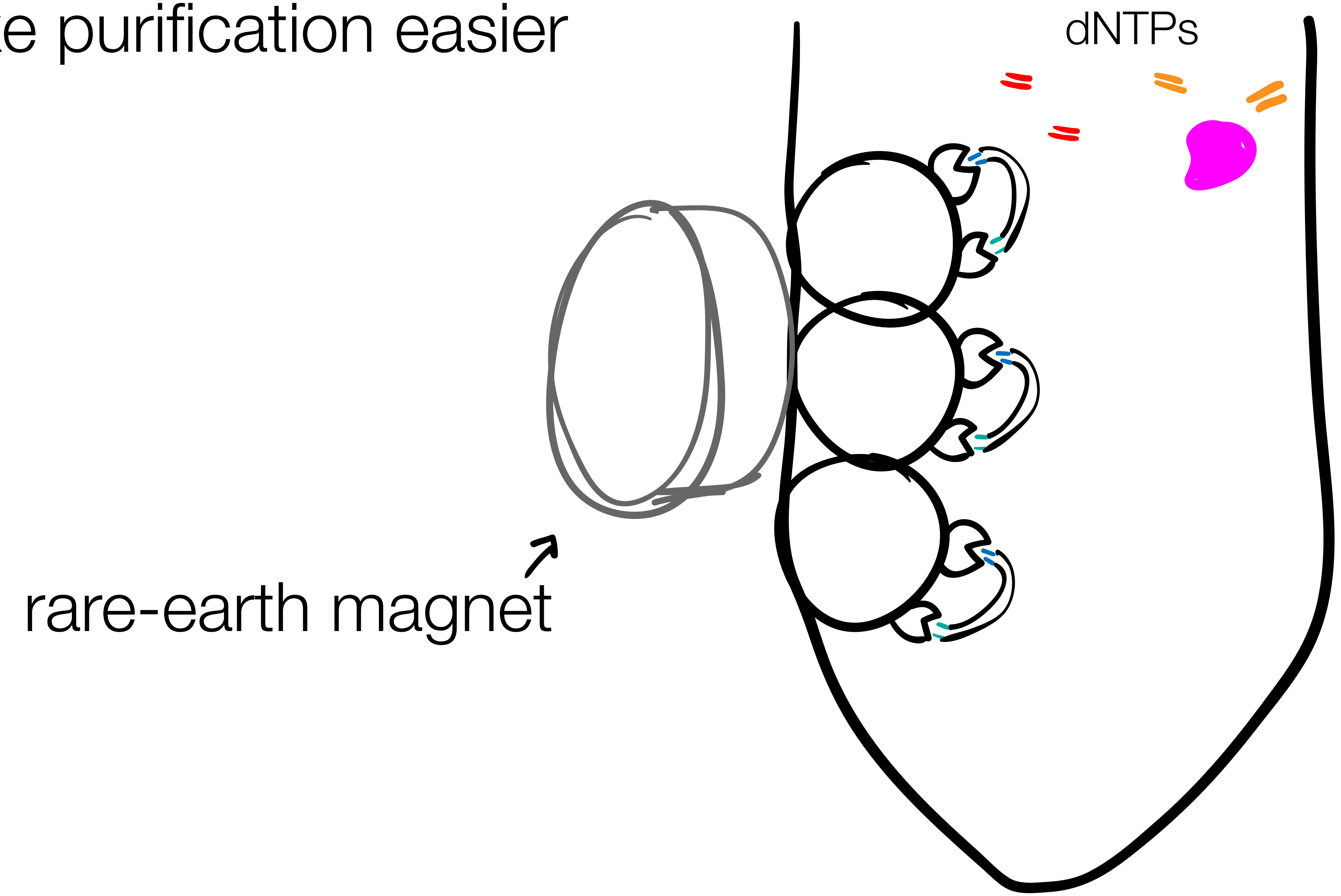

Preparing a sequencing ‘library’

fragmenting genomes and attaching adaptors

BLTs make purification easier

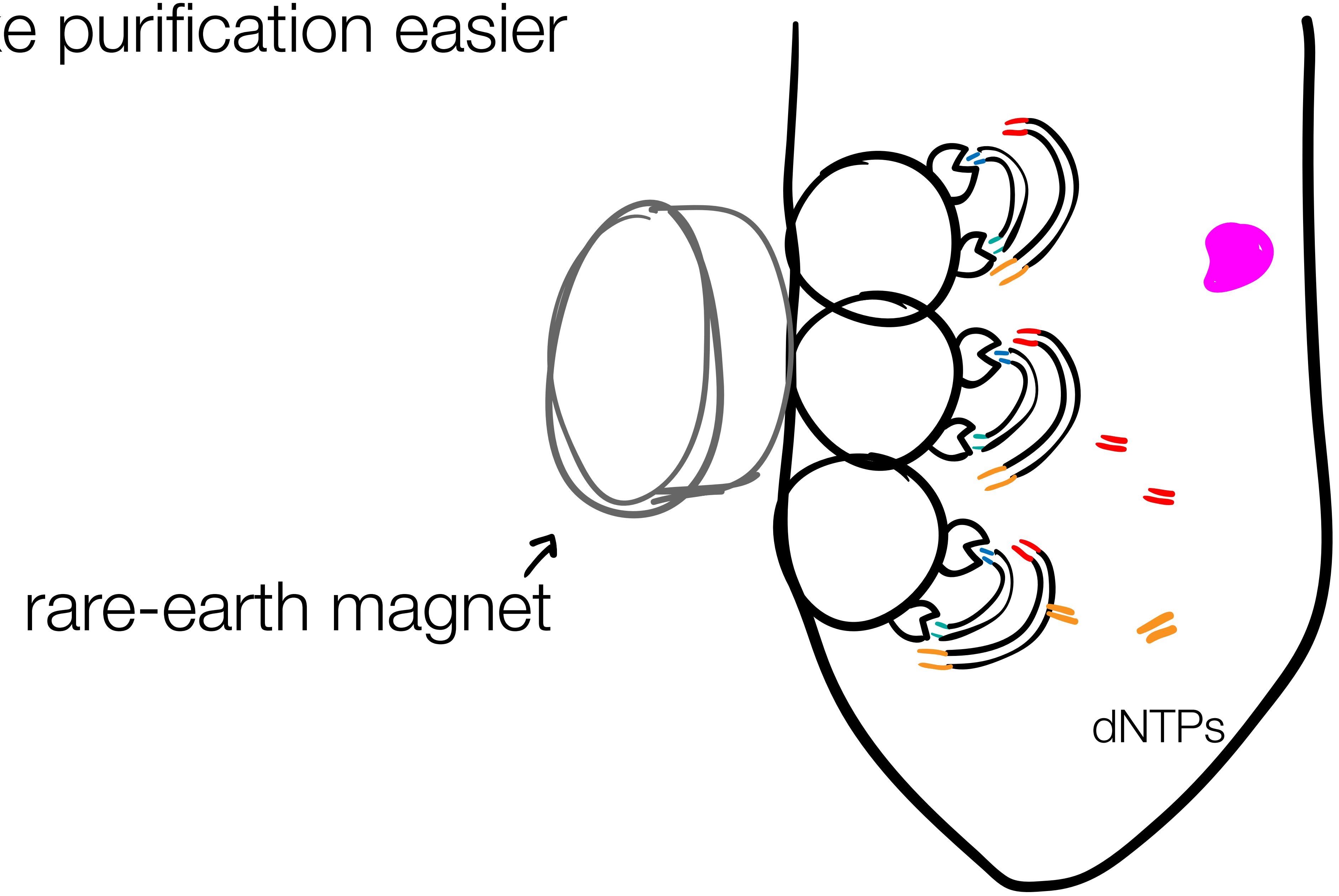

Preparing a sequencing ‘library’

fragmenting genomes and attaching adaptors

BLTs make purification easier

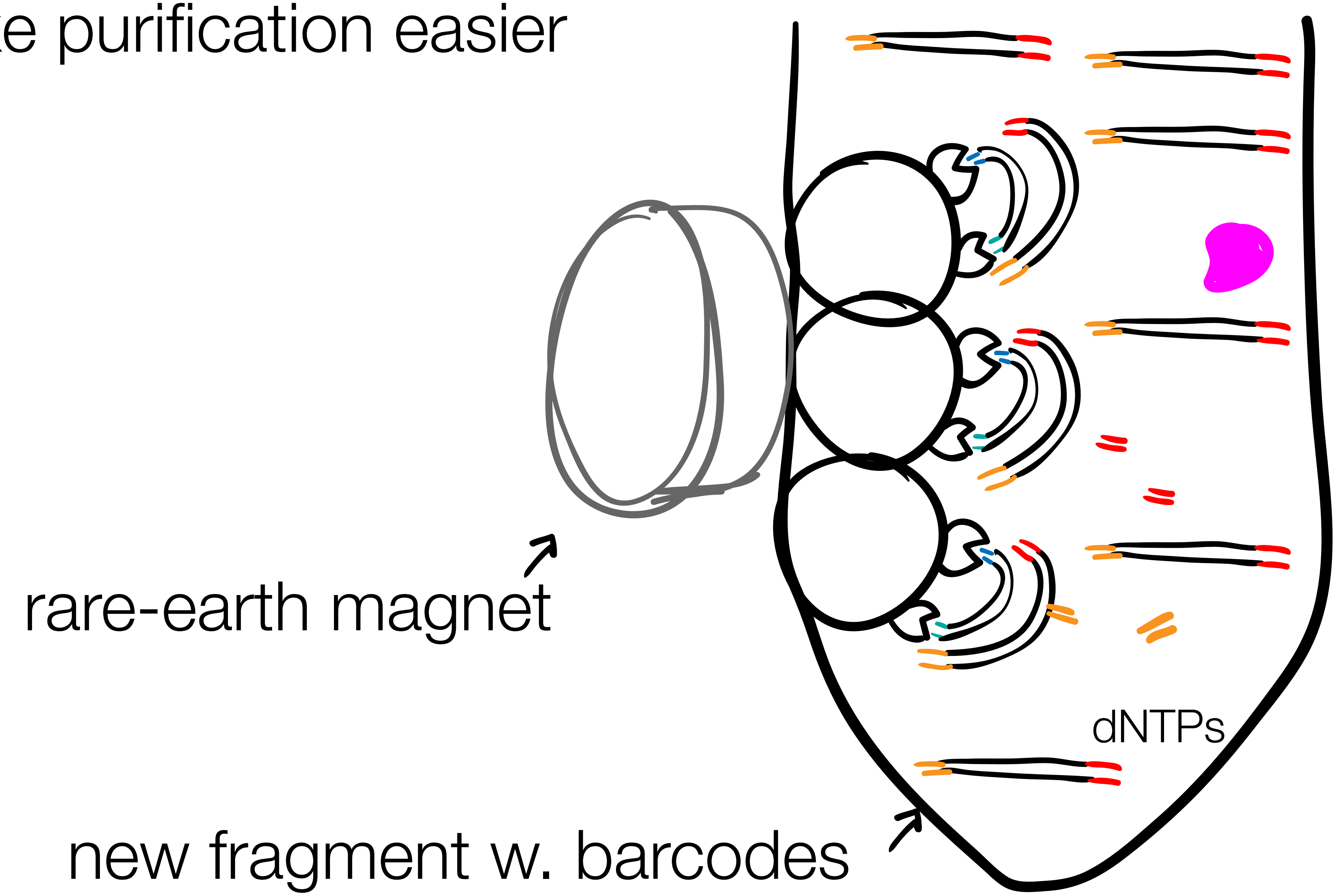

Preparing a sequencing 'library'

fragmenting genomes and attaching adaptors

BLTs make purification easier

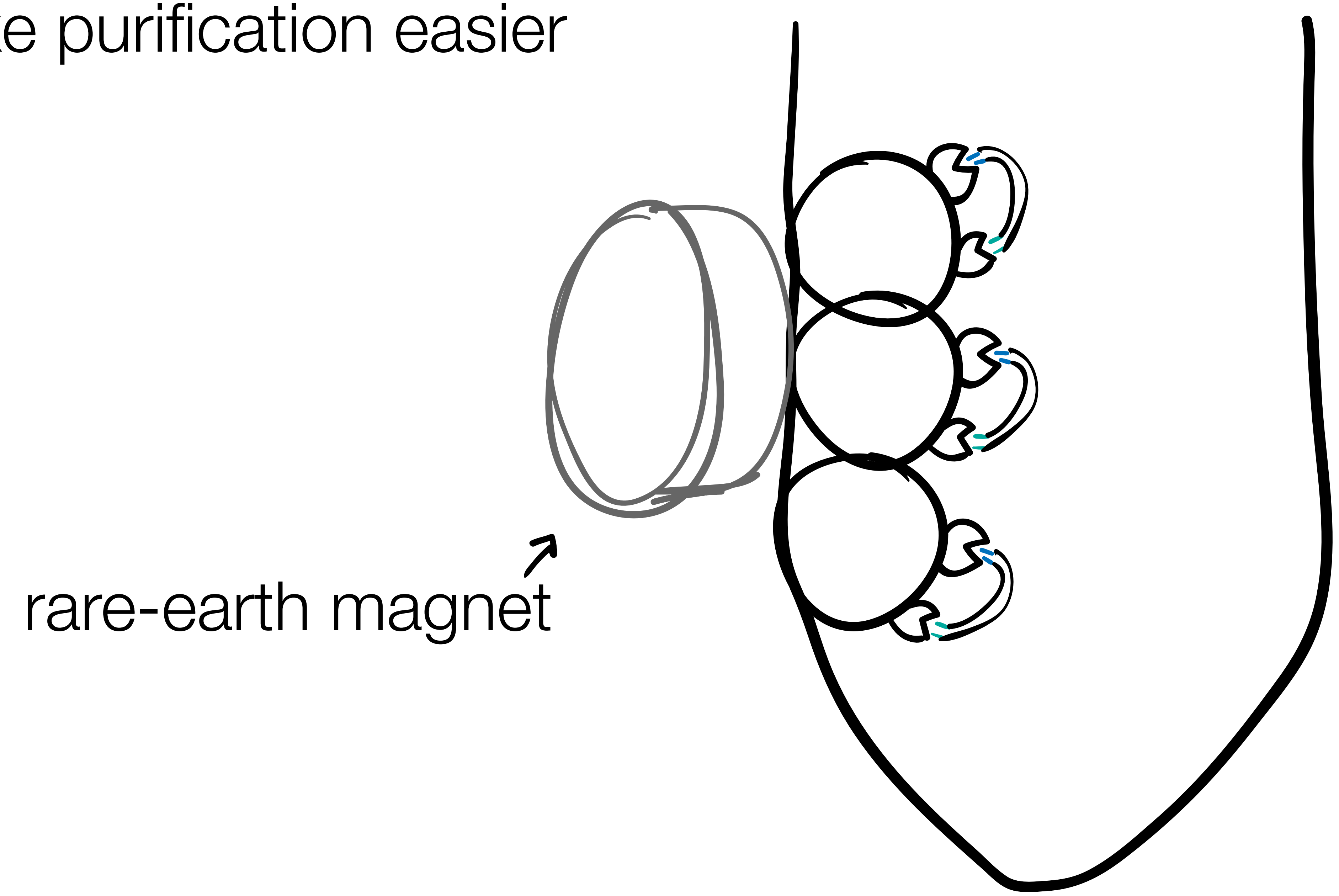

Preparing a sequencing 'library'

fragmenting genomes and attaching adaptors

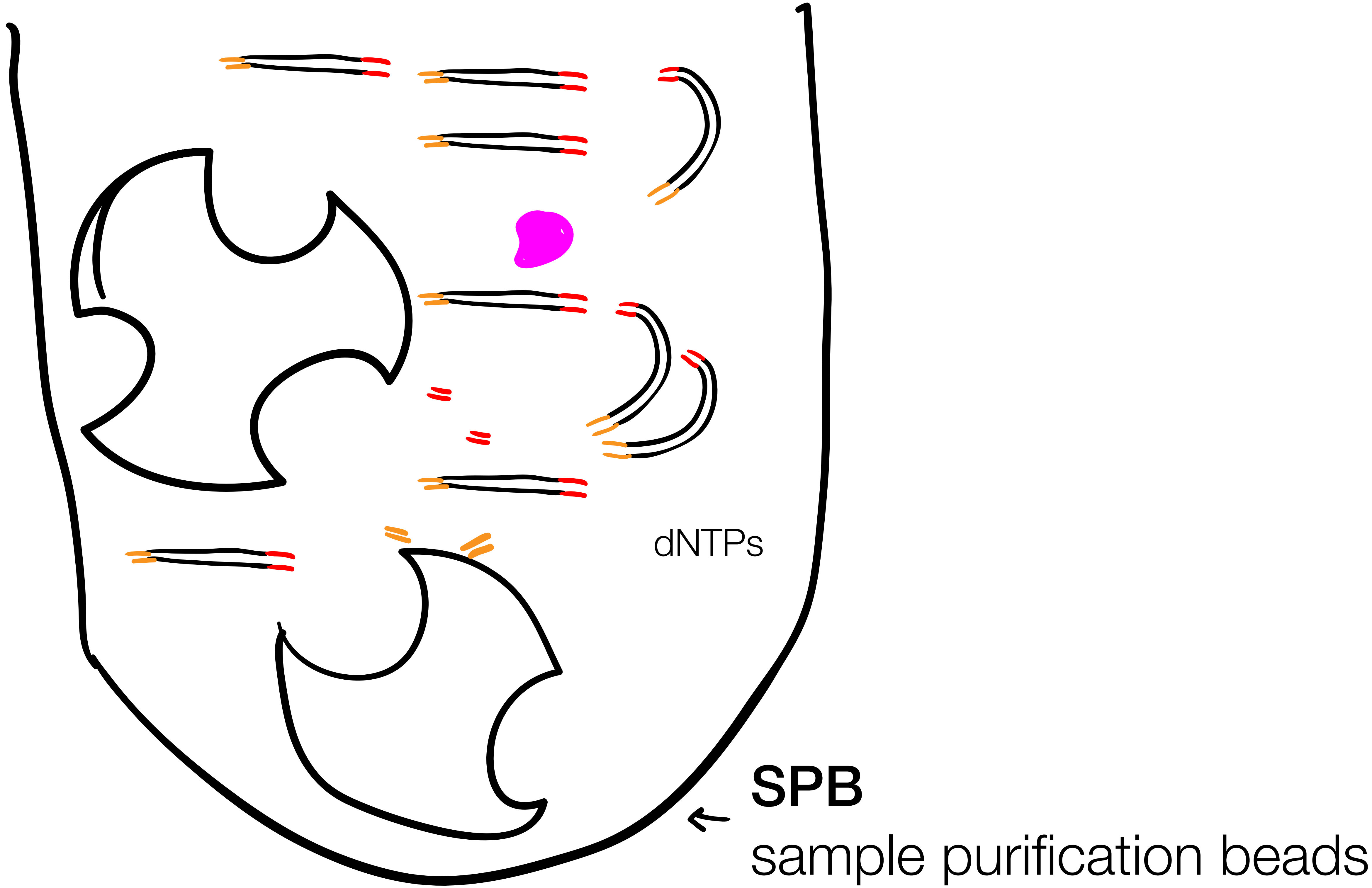

Preparing a sequencing 'library'

fragmenting genomes and attaching adaptors

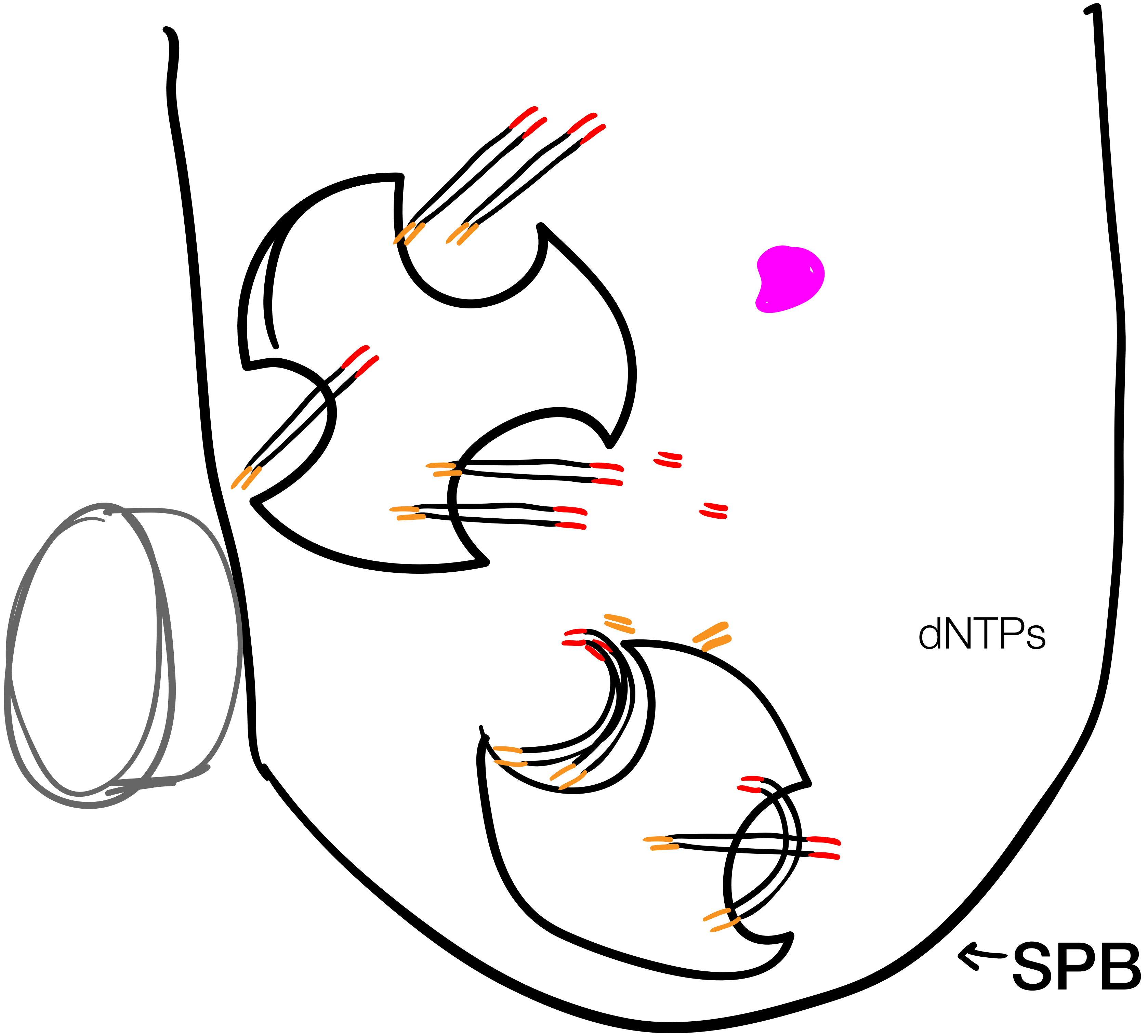

SPBs (sample purification beads) will bind DNA of the correct size until we change the solution to make them release the DNA

Preparing a sequencing ‘library’

fragmenting genomes and attaching adaptors

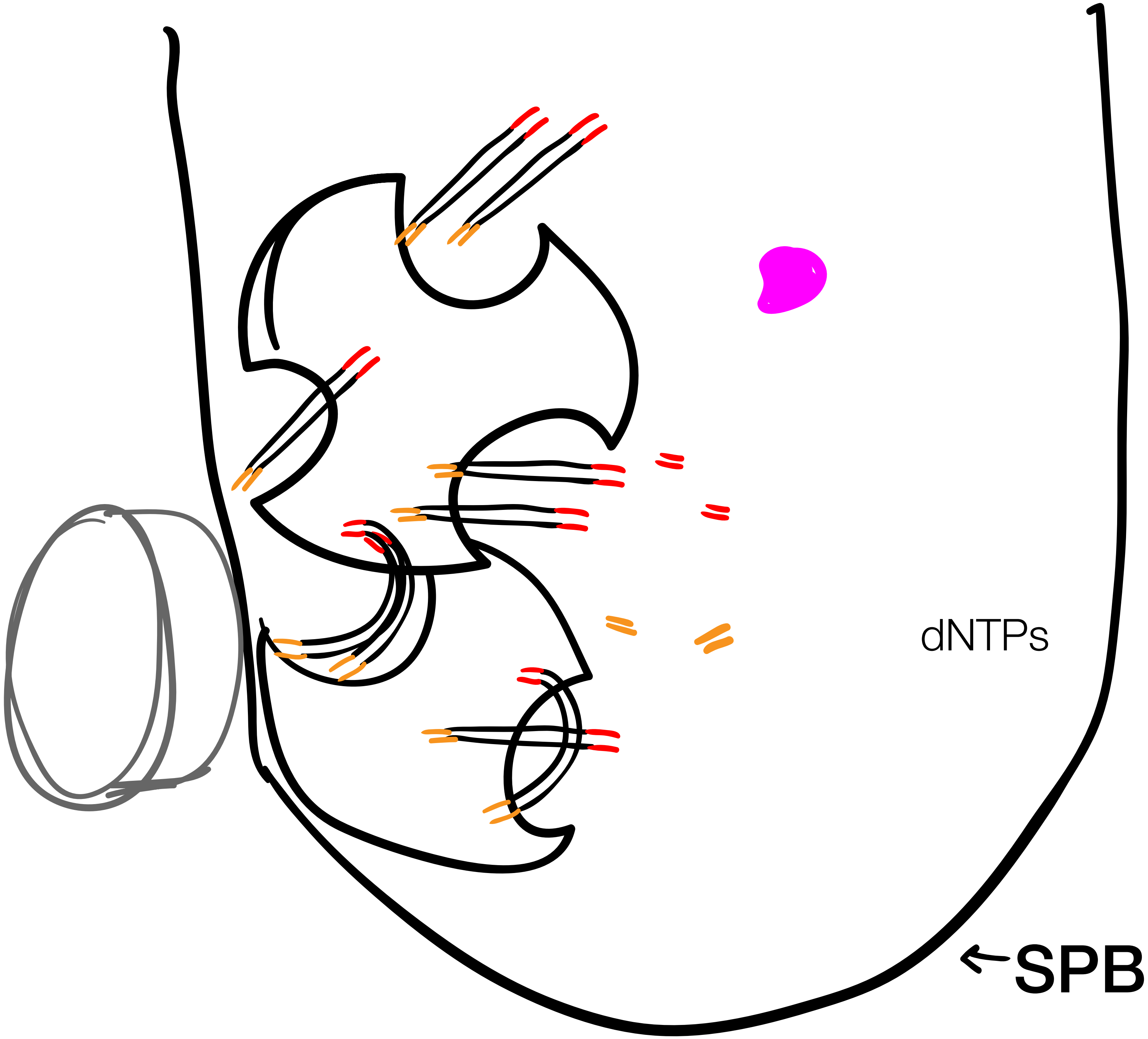

SPBs (sample purification beads) will bind DNA of the correct size until we change the solution to make them release the DNA

Preparing a sequencing ‘library’

fragmenting genomes and attaching adaptors

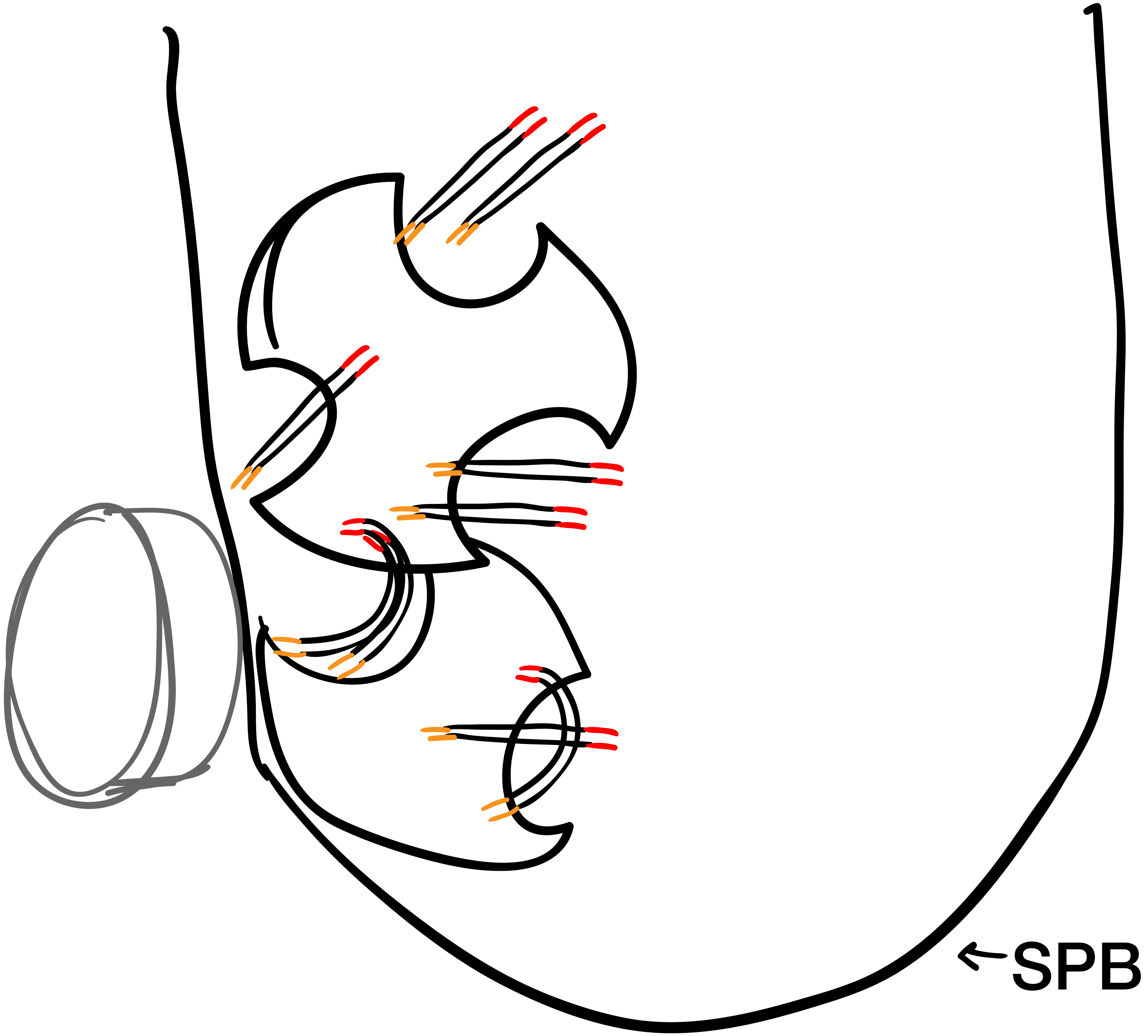

SPBs (sample purification beads) will bind DNA of the correct size until we change the solution to make them release the DNA
