# Supplementary material for: Resequencing of Microbial Isolates: A Lab Module to Introduce Novices to Command-Line Bioinformatics
Source: Front Microbiol. 2021 Mar 16;12:578859. doi: 10.3389/fmicb.2021.578859 (PMC8008064; doi:10.3389/fmicb.2021.578859)
Supplement: Supplementary file 4 [file Data_Sheet_4.PDF]

Command-Line Troubleshooting Guide

While it would be impossible to anticipate all command-line problems or errors that might be encountered in resequencing analysis, there are only a few common errors that make up the majority of issues. Here, we provide examples of those common errors (the messages that pop up on the terminal screen), and a guide for how to go about fixing them.

Note: sometimes these error messages are preceded by additional text or lines of text; make sure to scan through the message, see what it's saying and watch out for the examples below.

'File not found' errors

Sometimes when you type a command that tells the computer to go to a particular location (go to a directory or 'folder') or to do something with a particular file, you will get the following message:

```
No such file or directory
```

This can be especially frustrating if you KNOW the file or directory exists because you can see on your desktop where you just downloaded it or created it. What's happening here has to do with the terminal shell's extreme nearsightedness. As far as the terminal is concerned, very little exists outside the current directory where you are located, and you can only be 'inside' one directory at a time.

When you tell the terminal to do something with a file, it looks around the directory where you are, and if it doesn't see something with the exact name you specified, it gives up, and declares that the thing you are looking for doesn't exist.

How can you fix this problem? First, you need to know where you are. To find out, type the 'print working directory' command ('pwd'), below, and hit enter.

```
pwd
```

The terminal will spit back an answer showing the path - the nested set of folders within folders - where you currently are. This path is like an address for your current location. It will look something like the one below (but the names will reflect the actual directory names on your computer). In the example, the directory 'cool_resequencing_data' is inside of a directory called 'lab_stuff', which is inside the Desktop directory, which is inside the directory for your user account, which is inside the home directory for all users.

```
/Users/yournamehere/Desktop/lab_stuff/cool_resequencing_data
```

Is the thing you are looking for actually in the 'cool_resequencing_data' directory (or wherever you happen to be)? To find out, and to see what your terminal sees, use the 'list' command ('ls'), below, and

hit enter. Whenever you open a terminal window for the first time, or whenever you navigate to a new directory, it is good practice to type the ‘ls’ command to look around and see where you are.

```
ls
```

This will return a list of all the files and directories inside of the ‘cool_resequencing_data’ directory, and you can start to figure out why you are getting the ‘No such file or directory’ error based on what you see. There are a few possible options:

1. The thing you’re looking for isn’t in the list. Ooops. Either move the thing to your current directory, or navigate in terminal to the directory where the thing is, or specify the file with the full path to its correct location.

2. The thing you’re looking for is in the list, but there’s an obvious typo. Maybe when you typed the command you spelled it wrong, or capitalized it when you shouldn’t have. This is one reason ‘tab-complete’ is so helpful - after typing just the first few letters - it will do the rest automatically and accurately. Try the command again with the correct filename.

3. The thing you are looking for is in the list, and for the life of you, you can’t see any differences between the name in the list and the name you typed in your command. What’s going on? Sometimes, especially if you are copying and pasting commands from a pdf or website, the copy/paste function will pick up extra text characters, like spaces, that may be invisible, but make a mismatch between the command and what’s actually in the directory. Or sometimes they’ll change symbols, like dashes, to some other similar-looking symbol that also results in a mismatch. Try typing the command out manually instead of copy/pasting.

4. (Rare). The thing you are looking for isn’t in the list you see with ls, but it is there when you look at the folders normally, in your desktop window. You might be in a folder that has restrictions on what terminal can see and do (some ‘Applications’ folders that come with your computer are like this). Try moving the file to a different location; you may have to hold command while you are dragging it to prevent it from turning into a shortcut (aka an ‘alias’).

‘Command not found’ errors

Sometimes when you type a command to run a particular piece of software, you will get the following message:

```
command not found
```

There are a couple of possible explanations for this.

1. Is the software installed? Often, especially with bioinformatics software, simply downloading the software is not sufficient, and it actually has to be installed. Double check the installation

© Katherine Petrie, 2020 | Do not copy or distribute without permission

instructions from the developer for each software tool that you install and make sure to follow the directions.

2. Is the software program in your current location? Just like files, terminal only sees the software in your current location, unless you do something special (see 'creating a shortcut for software commands', below). The software file is a special type of file called a 'binary' which is the actual executable software program. When you type a command, it starts running the binary file. Is the binary file in your current location? Use 'ls' to find out. If it's there, you should see a filename matching the command itself.

If it's not there, you can try to locate it, and run the command from the folder where you find it. You can then carry out your analysis in that location. Alternatively, you can try creating a 'shortcut' for it. Most installation methods (including package managers) create the shortcut automatically, though a few may provide explicit directions for how to do so. The basics of creating the shortcut are described below, though you should consult the installation instructions for individual software programs carefully before proceeding, and please note that in most cases, you will not need to do this manually at all.

> *Creating a shortcut for software commands:* You may have noticed that all the common Unix commands (ls, pwd, cd, etc.) work perfectly fine no matter where you are, and there aren't copies of the binaries for those commands in every folder. So what's the secret?
>
> When you start terminal, your terminal session automatically creates a variable, called the $PATH, which is a list of locations where terminal should look for binaries whenever a command is typed. These locations are specified by addresses called 'paths' that describe nested sets of directories leading to the binaries.
>
> By default, the $PATH variable only specifies the location of the commonly used commands available to all users (this directory is actually located 'above' the 'Users' directory). It's generally good practice not to mess with that high-level universal directory, so when people install new software, they will usually instead add the directory where the software is to the $PATH.
>
> How do you do that? If you've already followed the installation instructions carefully, or if you used a package manager, you can check to see if the folder containing your binary is already part of your $PATH variable with the command below

```
echo $PATH
```

> This will return a list of paths (nested folders separated by forward slashes '/') which are themselves separated by colons ':'. Is the path to the folder containing the binary for your software on this list?

© Katherine Petrie, 2020 | Do not copy or distribute without permission

If it's not, you can temporarily add the directory where the binary is to your $PATH, using the command below (replace the directory names with the actual directory names for where your binary is located).

```
export PATH="$PATH:/Users/yourname/path/to/your/binary"
```

This will add your directory to the end of the list of directories in the $PATH. Run the echo $PATH command again to verify that the new directory was added. You will have to run this again every time you open a new terminal window.

To permanently add a directory to the $PATH, you have to edit a profile file that contains commands that are run, invisibly and automatically, every time you start a new, interactive terminal window on your computer. This profile also tells terminal where all of the basic system commands are, so it is important not to accidentally change it, or you could end up with a situation where none of your commands work (see below). Additionally, different operating systems use different types of profile files. As such, specific instructions for how to permanently edit your path are beyond the scope of this tutorial, and we encourage you to work with IT support at your institution if is necessary.

'Command not found' errors for common, simple shell commands

Sometimes you'll find yourself in a situation where you get the 'command not found' error message for even the simple Unix shell commands like 'ls' or 'cd'. What's going on?

```
command not found
```

There are two possible explanations for this.

1. Some operating systems (for example, MacOS 10.15 'Catalina' and later), use a slightly different shell languages in terminal (zsh instead of bash). You can find out which shell you have by typing the command below.

```
echo $0
```

In most cases, the newer shell uses the same commands and offers helpful features (i.e., it will suggest the correct command if you made a typo instead of telling you 'command not found'). However, the newer shells are sometimes more overprotective by default about what commands users have access to. If you are encountering this problem, you can check system preferences to see if this overprotectiveness is the source of the problem.

Go to System Preferences > Security & Privacy > Privacy tab > select 'Full Disk Access' > make sure the '+' next to terminal is checked. You may need administrator privileges on your computer to make this changes, so work with your institution's IT support if you are using an IT-supported computer lab.

© Katherine Petrie, 2020 | Do not copy or distribute without permission

2. If terminal already had Full Disk Access, or if you operating system doesn't have that as an option under the Privacy tab, there is probably something else going on. The most likely explanation is that your $PATH variable (mentioned above) was accidentally changed so that it no longer includes the location of all the standard shell command files. While this is unlikely to happen on IT-supported institutional computers, if it does, it is a fixable problem.

First, double check that this is what is going with the echo $PATH command. You should see the list of standard binary (/bin) directories for your computer. If you don't, it means the profile file (mentioned above) is not working or somehow needs edited, so that it properly sets the $PATH variable.

Since all operating systems may have different standard directories on the $PATH, we cannot specify all exact directories that should be included, nor can we specify exactly how to edit the profile file. However, it should be possible to find what should be on the $PATH, and what the profile should look like for your operating system with an internet search, or by consulting your IT-support.

© Katherine Petrie, 2020 | Do not copy or distribute without permission
