# Supplementary material for: Resequencing of Microbial Isolates: A Lab Module to Introduce Novices to Command-Line Bioinformatics
Source: Front Microbiol. 2021 Mar 16;12:578859. doi: 10.3389/fmicb.2021.578859 (PMC8008064; doi:10.3389/fmicb.2021.578859)
Supplement: Supplementary file 5 [file Data_Sheet_5.PDF]

Guide to Sample Dataset

Overview

If you would like to test out breseq before using it with your own data, or if you would like to incorporate only the bioinformatics portion of the module, we have provided a test dataset, based on real data we collected in our class.

The data comes from microcosm evolution experiments where glass vials (Fisher 03-339-22K, 70mm height x 28 mm diameter) containing 6 mL King's B media were inoculated with 10 µL of an overnight culture of *Pseudomonas fluorescens* strain SBW25, and left at room temperature, with loose caps, but no shaking or aeration, for 5 days. These conditions facilitate rapid diversification, as an oxygen gradient develops that gives a fitness advantage to microbes with mutations that enable them to live in a biofilm at the oxygen-rich air-liquid interface. Additionally, the biofilm-forming variants that colonize the surface niche produce a unique 'wrinkly spreader' colony morphotype when plated on King's B agar plates, while the ancestral planktonic variants form smooth, circular colonies (for further discussion, see Spiers, 2014, Green *et al.,* 2011, referenced in the main text).

After the static incubation, samples were homogenized, diluted, and plated to isolate individual colonies. Several of these (with the morphotypes noted below) were used to grow up overnight cultures, which were processed according to the methods described in this manuscript. Additionally, the ancestor was sequenced. Many lab strains are not perfect matches for the publicly available references used in analyzing the sequencing data, so data from the ancestor is needed to differentiate mutations that actually evolved in the microcosms from 'mutations' which were already present in the ancestor. The sample dataset consists of nine files. There are four samples (three evolved and the ancestor) with two fastq files each (the forward and reverse reads). There is also an annotated SBW25 reference sequence (SBW25reference.gbk).

Expected Results

The tables on the next pages shows each sample, the morphotype of the colony it came from, and the expected mutations when breseq is run (we used breseq version 0.35.4). After running breseq, students must first identify the mutations that occurred in the microcosm (i.e. those that are not also present in the ancestor). Here, the 'new' mutations are shaded in gray. Next, students are challenged to interpret the data, using information about each mutation's location (coding or intergenic), effect (synonymous or non-synonymous), and the description of the gene (or nearby genes) potentially affected. Instructors can ask students to research independently, or guide them through likely interpretations, using what is known about the wrinkly spreader morphotype (see Spiers, 2014, Green *et al.,* 2011, referenced in the main text).

One issue that students may encounter is that breseq doesn't always output the common names for most genes (it instead uses their locus tag as the 'gene name'). Students may need to use organism-specific databases (like the Pseudomonas Genome DB, https://www.pseudomonas.com, for this dataset) to find additional information. Additionally, NCBI occasionally changes the numbering scheme for loci annotation for a given

organism, and outside databases may not always reflect the most updated scheme. However, it should be possible to find the 'old' locus numbers within the GenBank reference file (in our case, we have students open the reference file with a text editor and use the 'find' function to look up each locus in the breseq output to find the corresponding old locus ID). Here, the gene found mutated in all three evolved samples (chemotaxis response regulator protein-glutamate methyltransferase), goes by the common name wspF, and is a negative regulator of diguanylate cyclase. Diguanylate cyclase produces cyclic-di-GMP, a signaling molecule which promotes cellulose production. When WspF function is impaired, more cyclic-di-GMP is produced, and this results in production of more cellulose, which holds the biofilm together as an extracellular polymer (see Lind et. al, 2015 for a further discussion of this and other genes that may contribute to the wrinkly spreader morphotype).

Expected Results (samples 19002 and 19008)

sample code	morpho-type	position	mutation	annotation	gene name	gene function
19002 (evolved)	wrinkly spreader	45,881	+G	intergenic (+65/+20)	PFLU_RS00225 → / ← PFLU_RS00230	aminopeptidase/HAD family hydrolase
		146,478	C→A	D172Y (GAC→TAC)	PFLU_RS00685 ←	hypothetical protein
		985,333	+C	intergenic (+17/-136)	pmbA → / → PFLU_RS04355	metalloprotease PmbA/hypothetical protein
		1,359,370	C→T	S252L (TCG→TTG)	PFLU_RS06055 →	chemotaxis response regulator protein-glutamate methylesterase
		3,447,984	(C)$_{5\rightarrow3}$	intergenic (+55/+23)	PFLU_RS15380 → / ← PFLU_RS15385	DUF1652 domain-containing protein/ UvrD-helicase domain-containing protein
		6,223,020	Δ132 bp	intergenic (-391/+10)	PFLU_RS27955 ← / ← PFLU_RS27960	GlxA family transcriptional regulator/ L-serine ammonia-lyase
19008 (evolved)	wrinkly spreader	45,881	+G	intergenic (+65/+20)	PFLU_RS00225 → / ← PFLU_RS00230	aminopeptidase/HAD family hydrolase
		146,478	C→A	D172Y (GAC→TAC)	PFLU_RS00685 ←	hypothetical protein
		985,333	+C	intergenic (+17/-136)	pmbA → / → PFLU_RS04355	metalloprotease PmbA/hypothetical protein
		1,359,517	G→T	S301I (AGC→ATC)	PFLU_RS06055 →	chemotaxis response regulator protein-glutamate methylesterase
		3,447,984	(C)$_{5\rightarrow3}$	intergenic (+55/+23)	PFLU_RS15380 → / ← PFLU_RS15385	DUF1652 domain-containing protein/UvrD-helicase domain-containing protein
		6,223,020	Δ132 bp	intergenic (-391/+10)	PFLU_RS27955 ← / ← PFLU_RS27960	GlxA family transcriptional regulator/ L-serine ammonia-lyase
		6,396,435	Δ145 bp	intergenic (+41/+81)	PFLU_RS28750 → / ← PFLU_RS28755	HD domain-containing protein/ ABC transporter permease subunit

Expected Results (samples 19010 and 19013)

sample code	morpho-type	position	mutation	annotation	gene name	gene function
19010 (evolved)	wrinkly spreader	45,881	+G	intergenic (+65/+20)	PFLU_RS00225 → / ← PFLU_RS00230	aminopeptidase/HAD family hydrolase
		146,478	C→A	D172Y (GAC→TAC)	PFLU_RS00685 ←	hypothetical protein
		985,333	+C	intergenic (+17/-136)	pmbA → / → PFLU_RS04355	metalloprotease PmbA/hypothetical protein
		1,358,834	(AGACGCCG)$_{1\rightarrow 2}$	coding (219/1011 nt)	PFLU_RS06055 →	chemotaxis response regulator protein-glutamate methylesterase
		3,447,984	(C)$_{5\rightarrow 3}$	intergenic (+55/+23)	PFLU_RS15380 → / ← PFLU_RS15385	DUF1652 domain-containing protein/ UvrD-helicase domain-containing protein
		6,223,020	Δ132 bp	intergenic (-391/+10)	PFLU_RS27955 ← / ← PFLU_RS27960	GlxA family transcriptional regulator/ L-serine ammonia-lyase
19013 (ancestor)	smooth	45,881	+G	intergenic (+65/+20)	PFLU_RS00225 → / ← PFLU_RS00230	aminopeptidase/HAD family hydrolase
		146,478	C→A	D172Y (GAC→TAC)	PFLU_RS00685 ←	hypothetical protein
		985,333	+C	intergenic (+17/-136)	pmbA → / → PFLU_RS04355	metalloprotease PmbA/hypothetical protein
		3,447,984	(C)$_{5\rightarrow 3}$	intergenic (+55/+23)	PFLU_RS15380 → / ← PFLU_RS15385	DUF1652 domain-containing protein/ UvrD-helicase domain-containing protein
		6,223,020	Δ132 bp	intergenic (-391/+10)	PFLU_RS27955 ← / ← PFLU_RS27960	GlxA family transcriptional regulator/ L-serine ammonia-lyase

A note on software installation before trying out the test dataset

As mentioned in the main text, you need to install breseq, fastqc, and the fastx_toolkit in order to complete the bioinformatics analysis. Software installation can be challenging and we recommend utilizing IT support at your institution, if possible. If you are using a package manager for the installation (like conda), carefully follow the download, installation and channel setup instructions specified in the 'Getting Started' section of the bioconda user documents found at https://bioconda.github.io/. We recommend using conda to create a specific environment (a workspace with a defined set of software) for your project; this way, you can be sure the software will work together with all of their dependencies, even if something gets updated in your regular computer environment. We also recommend that you create this environment with a fresh installation of R, in addition to the bioinformatics software that depend on it. We tested the data set in an environment set up with the following command:

conda create -n sampledataset r fastx_toolkit fastqc breseq

Lind, P. A., Farr, A.D., and Rainey, P.B. (2015). Experimental evolution reveals hidden diversity in evolutionary pathways. *eLife*. doi: 10.7554/eLife.07074
